# A revision of the genus *Muricea* Lamouroux, 1821 (Anthozoa, Octocorallia) in the eastern Pacific. Part II

**DOI:** 10.3897/zookeys.581.7910

**Published:** 2016-04-14

**Authors:** Odalisca Breedy, Hector M. Guzman

**Affiliations:** 1 Centro de Investigación en Estructuras Microscópicas, Centro de Investigación en Ciencias del Mar y Limnología, Escuela de Biología, Universidad de Costa Rica. P.O. Box 11501-2060, Universidad de Costa Rica, San José, Costa Rica; 2 Smithsonian Tropical Research Institute, P.O. Box 0843-03092, Panama, Republic of Panama

**Keywords:** Alcyonacea, Cnidaria, eastern Pacific, Muricea, plexaurid gorgonian, soft corals, taxonomy

## Abstract

The species of the genus *Muricea* were mainly described from 1846 to 1870. After that very few contributions were published. Although the highest richness of *Muricea* species is in the eastern Pacific shallow waters, a comprehensive systematic study of the genus does not exist. Recently we started a taxonomic review of the genus in order to validate the status of four species previously included in the genus *Eumuricea*. Herein we present the second part of the *Muricea* revision dealing with the species-group characterised by shelf-like calyces instead of tubular-like calyces (the *Muricea squarrosa*-group). Original type material was morphologically analysed and illustrated using optical and scanning electron microscopy. Comparative character tables are provided for the genus. The taxonomic status of the species was analysed and established by designating lectotypes, alternatively by recognising a holotype by monotypy. We conclude that the genus *Muricea* comprises 20 valid species, including the previous four in the *Muricea squarrosa*-group. We propose 10 lectotypes, a new combination and three more species groups for the genus based on morphology: the *Muricea fruticosa*-group, *Muricea plantaginea*-group and *Muricea austera*-group.

## Introduction

The genus *Muricea* Lamouroux, 1821 is widely distributed along the eastern Pacific shallow waters (less than 40 m deep), and although it has representatives in the western Atlantic, the highest species richness is attained in the eastern Pacific. We deal with the eastern Pacific *Muricea* species in the present taxonomic review. The first record appeared as *Gorgonia plantaginea* Valenciennes, 1846 (Plate 15, Fig. 14A, herein) from Mazatlan, México, collected during the French expedition, “Voyage autour du monde sur la frégate la Vénus” (Valenciennes 1846). The major contribution to the knowledge of this taxon was made by Verrill (1868–1870) in his paper “Notes on Radiata”, where he described 16 new species and revised the former records. Other contributions were made in the XX century: Kükenthal (1919, 1924) published a key and a short review of the described species; Hickson (1928) described two *Muricea* species from the Pacific coast of Panamá; Aurivillius (1931) described three more species, pointing out that he did not have access to the type material of the former described species so his new species were uncertain to some extent; Stiasny (1941, 1943) also wrote about the octocorals of Panamá and reviewed the species previously described by Hickson (1928); and finally Deichmann (1941) described a new species from the Galápagos Islands, Ecuador. After this, no taxonomic research was conducted dealing with the genus. Hardee and Wicksten (1996) re-described and compared three *Muricea* species from California, USA. The status of most species is uncertain because previous authors did not designate holotypes and the specimen and sclerite illustrations are poor in most cases. Some species have been described from a few specimens or from fragments, and type material was lost or misplaced. Therefore, a comprehensive review of this genus is inexorable for the advancement of ecological and evolutionary research in soft corals.

Recently, we published the first part of the review of *Muricea* (Breedy and Guzman 2015) dealing with the species assigned by Verrill (1869, p. 449) to the genus *Eumuricea* Verrill, 1869 and concluded that there is not enough support to keep these genera apart. Therefore *Eumuricea* is treated as a synonym of *Muricea*, comprised of four valid and one dubious species: *Muricea acervata* Verrill, 1866; *Muricea hispida* Verrill, 1866; *Muricea squarrosa* Verrill, 1869; *Muricea tubigera* Verrill, 1869; and *Muricea horrida* Möbius, 1861 (sp. dubia).

The present research represents the second part of the fifth review, in a series of six proposed, aiming to evaluate the genera of gorgonians historically reported for the shallow eastern Pacific waters. Previous reviews dealt with *Pacifigorgia* Bayer, 1951 (Breedy and Guzman 2002), *Leptogorgia* Milne, Edwards & Haime, 1857 (Breedy and Guzman 2007) and *Eugorgia* Verrill, 1868a (Breedy et al. 2009), in the family Gorgoniidae; *Heterogorgia* Verrill, 1868b (Breedy and Guzman 2011) and *Muricea* Lamouroux, 1821 (Breedy and Guzman 2015), in the family Plexauridae.

## Material and methods

### Acronyms

CASIZ
 California Academy of Science, Invertebrate Zoology, San Francisco, USA 


CDRS
Charles Darwin Research Station, Galápagos, Ecuador 


CIMAR
 Centro de Investigación en Ciencias del Mar y Limnología, Universidad de Costa Rica, San José, Costa Rica 


CIEMIC
 Centro de Investigación en Estructuras Microscópicas, Universidad de Costa Rica 


CRBMco
 Colección de referencia de Biología Marina Universidad Del Valle, Cali, Colombia 


ICZN
 International Code of Zoological Nomenclature 


IMARPE
 Instituto del Mar de Perú, Lima, Perú 


INN
 NAZCA Instituto de Investigaciones Marinas, Salinas, Ecuador 


MCZ
Museum of Comparative Zoology, Harvard University, Boston, USA 


MNHN
Muséum National d’Histoire Naturelle, Paris, France 


MNHUK
Museum of Natural History (former BM, British Museum), London, UK 


MZUF
 Museo Zoologico dell’Università di Firenze, Firenze, Italia 


MZUT
Museo Regionale di Scienze Naturali, Torino, Italia 


RMNH
 Netherlands Centre for Biodiversity Naturalis, Leiden, (former National Museum of Natural History Naturalis) 


SEM
 Scanning Electron Microscopy 


SMNH
Swedish Museum of Natural History, Stockholm, Sweden 


STRI
Smithsonian Tropical Research Institute, Panamá 


UCR / MZUCR
 Museo de Zoología, Universidad de Costa Rica, Costa Rica 


UNAM
Universidad Nacional Autónoma de México, México 


UNIANDES-BIOMMAR
Universidad de Los Andes, Laboratorio de Biología Molecular Marina, Bogotá, Colombia 


UPCH
 Colecciones Biológicas, Universidad Peruana Cayetano Heredia, Lima, Perú 


NMNM
 National Museum of Natural History, Smithsonian Institution, Washington, USA 


YPM
 Yale Peabody Museum of Natural History, New Haven, USA 


ZMHC
Zoologisches Institut und Zoologisches Museum der Universität Hamburg, Germany 


ZMUC
Zoologisk Museum Kobenhavn, Danmark 


ZSM
Zoologische Staatssammlung München, Deutschland 


The type specimens and comparative reference material used in this study were analysed during visits to museums or acquired on loan from the CASIZ, MCZ, MNHN, MNHUK, NMNM and YPM. Other collections that house type material MZUF, MZUT, ZSM were revised. In addition, specimens recently collected along the Pacific coast of Costa Rica, Ecuador, Mexico, Panamá and Perú deposited in the UCR, STRI and other institutions (CDRS, CRBMco, IMARPE, INN, UNAM, UNIANDES-BIOMMAR, UPCH) were studied. The material was collected by scuba diving, down to 45 m in depth, and some specimens were obtained by dredging down to 60 m.

### Morphological study

The microscopic study was done at the CIEMIC, the specimens were prepared for SEM following the protocol described in Breedy and Guzman (2002) and the pictures were obtained using Hitachi SEMs: S-570, N-S2370 and S-3700N. For optic microscopy, sclerites were mounted in water or glycerin and photographed with an Olympus LX 51 inverted microscope. Sclerites of the coenenchyme and calyces are very varied in size and form; the prevailing types are illustrated and described here. Measurements of the sclerites were obtained from pictures and directly from the microscope using an optical micrometer. The length of the sclerites was measured from one tip to the other and the width was taken from the most distant points across the sclerites, in some cases including the length of the longest spine, reporting the largest sizes found in the samples and in some cases, a range of variation. In case of curved sclerites, the length reported was without taking in account the curvature. The sclerites were illustrated by SEM micrographs. We also present optic microscope micrographs for colour details and sclerite composition. The diameter of the branches, branchlets, and stems are given taking in account the length of the calyces. Most of the analysed type material is dry and old. The drying or preservation process can affect the diameter of branches in general, and especially the calyces, however, we have observed that the tendency of the calyces (slightly raised, prominent) is kept after preservation and is consistent with living colonies (Breedy and Guzman 2015). In general, the colours of the colonies and sclerites are stable, and persist after fixation. Some species tint the alcohol when preserved, but keep their colours. Some fading is observed in dry specimens. When possible we mention the colour of the colonies alive, preserved and dry.

Data on geographical distribution are from our personal collections, museum catalogues and published monographs. Verrill and the other authors who described *Muricea* species (Aurivillius, Hickson Deichmann and Valenciennes) did not designate holotypes and in cases, the descriptions could often fit several species. In some cases, only one specimen in the collection was under a species name that automatically constitutes the holotype by monotypy. When the status was unclear we designated lectotypes to establish the identity of the poorly defined species and to avoid future confusion.

The terminology is according to Bayer et al. (1983), modifications made by Breedy and Guzman (2015) and others defined herein.

Comparative character tables are provided for the *Muricea* species (Tables 1–2).

**Table 1.** Sclerite comparison for the eastern Pacific *Muricea* species. Measurements given are from the holotypes and paratypes, in mm.

Species	Sclerite colours	Anthocodial sclerite colours	Dominant type of coenenchymal and calycular sclerites	Coenenchymal and calycular spindles maximum size	Anthocodial maximum size
*Muricea fruticosa*	w, rb, py	yellow, w	uss	2.0×0.5	0.64×0.1
*Muricea formosa*	w	w	uss	1.5×0.25	0.175×0.075
*Muricea aspera*	pb, w	w	uss	1.35×0.35	0.25×0.04
*Muricea echinata*	o, lb	lo, w	uss	2.4×0.34	0.25×0.05
*Muricea galapagensis*	amb, lo	lo, w	uss	4.1×0.75	0.25×0.06
*Muricea plantaginea*	rb, amb/w	lo, lb/w	ls	1×0.2	0.25×0.08
*Muricea californica*	ro,ly,amb	lo	ls	0.54×0.2	0.23×0.06
*Muricea mortensenii*	w	w	s	0.7×0.12	0.21×0.08
*Muricea austera*	rb,o,ly	w	uss	1.5×0.5	0.36×0.06
*Muricea albida*	w	w	uss	1.4×0.05	0.25×0.03
*Muricea crassa*	rb	lb, w	uss	2.5×0.7	0.22×0.04
*Muricea retusa*	rp, p,o	o, ly	uss	1.2×0.6	0.4×0.1
*Muricea purpurea*	dr, ro	ro	ls	0.7×0.3	0.3×0.055
*Muricea hebes*	py	py	uss	0.83×0.20	0.45×0.1
*Muricea nariformis*	bo,lb	o	ls	0.52×0.28	0.17×0.035
*Muricea robusta*	o, bo, lb	o	ls	0.64×0.26	0.15×0.05

Colours: amb , amber ; bo , brownish orange ; lb , light brown ; lo , light orange ; ly , light yellow ; o , orange ; p , purple ; pb , pale brown ; py , pale yellow ; rb , reddish brown ; ro , reddish orange ; rp , reddish purple ; w , white, colourless Type of coenenchymal and calycular sclerites: ls , leaf-like spindle ; s , warty spindles ; uss , unilateral spinose spindles “-” no data; “?” uncertain

**Table 2.** Comparative features of the eastern Pacific *Muricea* species. Measurements given are from the holotypes and paratypes, in mm.

Species	Colony colour	Colony shape	Branching pattern	Length of unbranched terminal branchlets	Diameter of end branchlets (mm)	Coenenchyme	Calyx height at branchlets	Calyx arrangement at branchlets
*Muricea fruticosa*	rb, w, bi	bu	irr	15–40	3–6	t	1–1.2	c
*Muricea formosa*	w	bu	irr lat, dich	28	6–9	t	2.8–3	c
*Muricea aspera*	lb	fla ?	irr, lat	6–30	4–5	t	1–2	c, slightly imbr
*Muricea echinata*	rb	bu	irr, lat	6–30	5–8	t	2.8–3	c
*Muricea galapagensis*	lo	fall	lat	80	1.6–3	t	0.6–1	s
*Muricea plantaginea*	db/w	fla	irr, lat	10–50	2–3	t	0.7–1.2	c, imbr
*Muricea californica*	ro	bu	irr, lat	0.5–2.8	3–3.2	mt	1.1–1.9	c, slightly imbr
*Muricea mortensenii*	py	fla	irr	2–4	2–3	t	0.7–1	c
*Muricea austera*	rb	bu	dich, lat	50	7–8	T	1.7–2	c
*Muricea albida*	w	cand	dich	11	5–7	T	0.8–1.8	c, slightly imbr
*Muricea crassa*	db	bu	dich, lat	70	7–10	T	2.7–3	c
*Muricea retusa*	rp	-	dich	50	7–8	T	1–1.5	c
*Muricea purpurea*	rp	bu	dich	50–80	9–11	T	1.5–1.8	c, slightly imbr
*Muricea hebes*	yb	fing	dich	32	6–9	T	1–1.8	c, slightly imbr
*Muricea nariformis*	bo	fing	dich	24	57	T	0.8–1.2	c
*Muricea robusta*	bo	bu	dich	70	7–8.5	T	0.7–1.2	c

Colours: bi , bicoloured ; bo , brownish orange ; db , deep brown ; lo , light orange ; lb , light brown ; py , pale yellow ; rb , reddish brown ; ro , reddish orange ; rp , reddish purple ; yb , yellowish brown ; w , white, colourless . Colony shape: bu , bushy ; cand , candelabrum ; fall , falling branches ; fla , flabelliform ; fing , finger-like Branching pattern: dich , irregularly dichotomous ; irr , irregular ; lat , lateral Coenenchyme: t , thin , mt , medium thickness ; T , thick Calyx arrangement at branchlets: c , close, not imbricate ; imbr , imbricate ; s , sparse “-” no data; “?” uncertain

### Terminology

*unilateral spinous spindles*: spindle often massive, sculptured on inner surface by crowded complex tubercles and on outer surface by simple spines or prickles, and in some species with a few more or less prominent coarse, prickly projections.

*prickly spindle*: warty, irregular spindle with prickly, pointed processes at one tip.

*leaf-like spindle*: warty spindles with flat, leaf-like processes, terminal or laterally placed.

*spinous club-like spindle*: club shaped sclerite, with head ornamented by thorny or leafy, sometimes unilaterally placed processes and with warty, thin handle.

### Notes on morphological characters

The most informative characters for the genus are the branch diameter, the colours (colonies and sclerites) and the type, size and combination of sclerites. The colours, although some variation exist, are very constant and a reliable characteristic (Breedy and Guzman 2015). Branch diameter and colony form could vary according to the age of the colony and the exposure to the currents, but in general it is possible to have a primary approximation for identification purposes. Calyx size and spacing vary from the larger branches to the thinner in most of the species, being larger, acuter and closer set at the branchlets and shorter, blunter, and more distantly arranged at the main branches. The sclerites, which are the most informative characters, show a continuum in shapes. It is difficult to find limits to the variety of forms; for example, a prickly spindle is basically a modified leaf spindle, or a leaf-like spindle - as defined here is as described in the octocoral glossary (see Bayer et al. 1983). There are many intermediate sclerite forms and it is not sensible to name each and every one. In most cases it is not one type of sclerite that defines a species, it is rather the combination of forms, colours, and sizes. We made approximate descriptions of the sclerite types, but the illustrations that we present for each species are the best reference, and intra-specific variation could be deduced from those. The polyp sclerites were basically warty rods and spindles arranged in points at the base of the tentacles or longitudinally set along the polyp neck zone. In some cases, small leaf-like spindles are also part of the anthocodiae. Undeveloped sclerites were found by dissecting polyps, in some cases the same types are found in the axial sheath. In species with thin and deteriorated coenenchyme it was not possible to determine different sclerite layers. But it seems that the axial sheath contains the undeveloped sclerites, capstans, radiates and spindles, while the external coenenchyme, is constituted by unilateral spinous, leaf-like, warty or prickly spindles, either a specific type or a combination of them. These are the same sclerites that constitute the external calyx wall. There is also a combination of sclerites that make the internal calyx wall, especially the leaf-like, warty, or prickly spindles, and various types of clubs. The spiny tips of some of these sclerites project beyond the calyx border in many species, giving a prickly appearance to the colonies; because of that, some *Muricea* species are commonly known as spiny sea fans.

Presently, the boundaries among octocorals species are based on common characters, basically colony and sclerite shapes and colours that could be influenced, at certain point, by the environment. For that, in determining an octocoral morpho-species the combination of these morphological characteristics results in a more accurate assessment. Furthermore, analysis of several specimens aid in identifying intraspecific variation. Field observation and evaluation of habitat in terms of oceanographic conditions could provide information in the decision making process.

## Systematics

### Class Anthozoa Ehrenberg, 1834 Subclass Octocorallia Haeckel, 1866 Order Alcyonacea Lamouroux, 1812 Family Plexauridae Gray, 1859

#### Genus Muricea

Taxon classificationAnimaliaAlcyonaceaPlexauridae

Lamouroux, 1821

Muricea Lamouroux, (pars.) 1821: 36; Blainville (pars.) 1834: 509; Ehrenberg (pars.) 1834: 134; Dana 1846: 673; Milne Edwards and Haime 1857: 142; Kölliker 1865: 135; Verrill 1868b: 411; Verrill 1869: 418–419, 450; Studer 1887: 58; Wright and Studer 1889: 93; Gorzawsky 1908: 8; Nutting 1910: 9; Kükenthal 1919: 835; 1924: 141; Riess 1929: 383–384; Aurivillius 1931: 102–104; Deichmann 1936: 99; Bayer 1956: F210; 1959: 12; 1961: 179–180; 1981: 930 (in key); 1994: 23–24; Tixier-Durivault 1970: 154; Harden 1979: 140; Hardee and Wicksten 1996: 127–128; Marques and Castro 1995: 162; Castro et al. 2010: 779.
Eumuricea (pars.) Verrill, 1869: 449; Riess 1929: 397.

##### Type species.

*Muricea spicifera* Lamouroux, 1821, by subsequent designation: Milne Edwards and Haime 1857. [*Muricea spicifera* was later synonymised with *Muricea muricata* (Pallas, 1766) after Bayer 1961: 179–180].

##### Diagnosis

(based on Breedy and Guzman 2015). Colonies planar or multiplanar, bushy, arborescent, laterally branched, pinnately branched, dichotomous or with long flexible branches, with some occasional branch anastomosis. Branches and branchlets upward bending almost parallel, and with about the same thickness all along, frequently with slightly enlarged tips. Coenenchyme moderately to very thick (compared to other plexaurids) with a circle of longitudinal canals surrounding the axis and dividing the coenenchyme into a thin inner layer or axial sheath, and a thicker outer layer. Polyps fully retractile within prominent calyces longitudinally and closely placed all around the branches and branchlets, or spaced in loose spirals around the branches and branchlets. Calyces prominent, shelf-like or tubular, with prickly projecting spindles, longitudinally arranged. Base of the anthocodia without sclerites or with flat rods arranged in weakly differentiated collaret and points below the tentacles, or just transversely set along the neck zone of the polyp. Sclerites of the outer coenenchyme and of the calyx mostly long, unilateral spinous spindles, often massive, sculptured on inner surface by crowded complex tubercles and on outer surface by simple spines or prickles, and in some species with a few more or less prominent coarse, prickly projections. Spindles with laterally placed spinous or leaf-like processes are the dominant type in some species. Axial sheath composed of capstans, spindles, or oval forms, and undeveloped sclerites. Sclerite colours are white, various hues of yellow, amber, orange, purple and red. Anthocodials with lower hues.

##### Distribution.

From Cape Hatteras, North Carolina to Brazil, including Bahamas, Greater and Lesser Antilles, Gulf of México, and Caribbean islands (Bayer 1961); in the eastern Pacific from southern California to Perú and presumably in Chile. The genus occurs at depths down to 200 m, but normally found less than 100 m. *Muricea midas* Bayer, 1959 is the deepest record for the genus in the western Atlantic (Bayer 1959); and *Muricea galapagensis* Deichmann, 1941 in the eastern Pacific.

##### Remarks.

Based on calyx morphology, Verrill (1869) subdivided *Muricea* from the eastern Pacific in two main groups, one with tubular calyces (former *Eumuricea*, see Breedy and Guzman 2015) and the other with shelf-like calyces. As Breedy and Guzman (2015) noted, there are many intermediate forms referring to calyx structure and two extreme structures: tubular and shelf-like. For this reason the more sensible alternative was the division of the genus in two groups: the first, having tubular calyces (already revised by Breedy and Guzman 2015) and the second, having shelf-like calyces (in this work). The second group might be subdivided according to the branch thickness and the dominant type of sclerites of the outer coenenchyme and the calyx. The shelf-like structure of the calyx could vary from prickly prominent to slightly raised borders, and from raised adaxial borders to minute abaxial rims blending with the coenenchyme. Species groups based on the former characters are proposed herein.

#### Muricea fruticosa group

Taxon classificationAnimaliaAlcyonaceaPlexauridae

**Figure 1.**
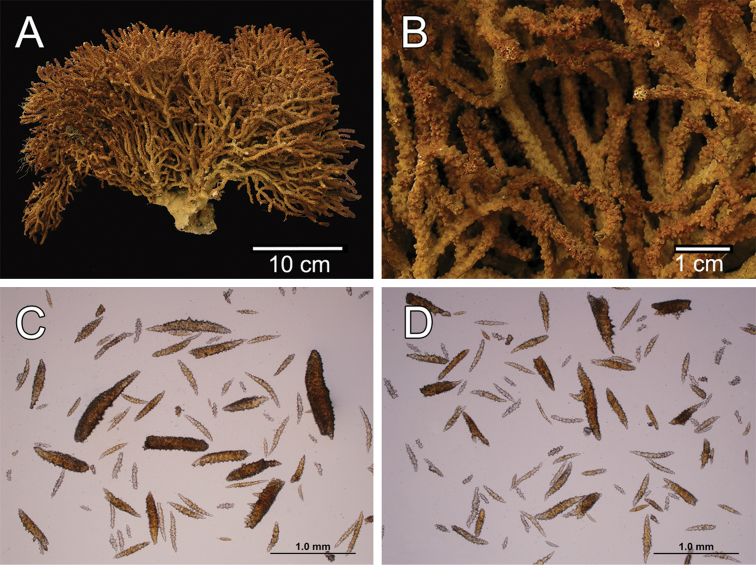
*Muricea fruticosa* Verrill, 1869 YPM 1574c. **A** Colony **B** Detail of branches **C–D** Sclerites, light micrograph.

**Figure 2.**
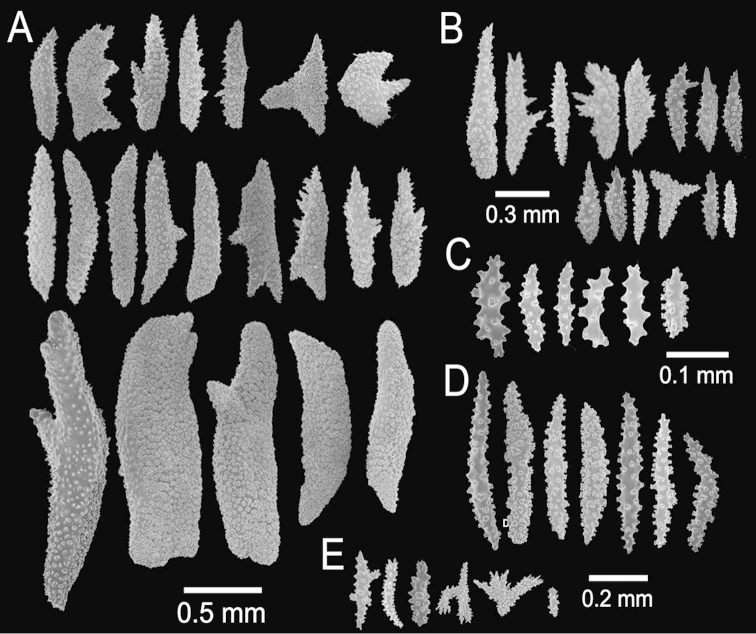
*Muricea fruticosa* Verrill, 1869 YPM 1574c. **A–B** Calycular and coenenchymal sclerites **C–D** Axial sheath **E** Anthocodial sclerites.

**Figure 3.**
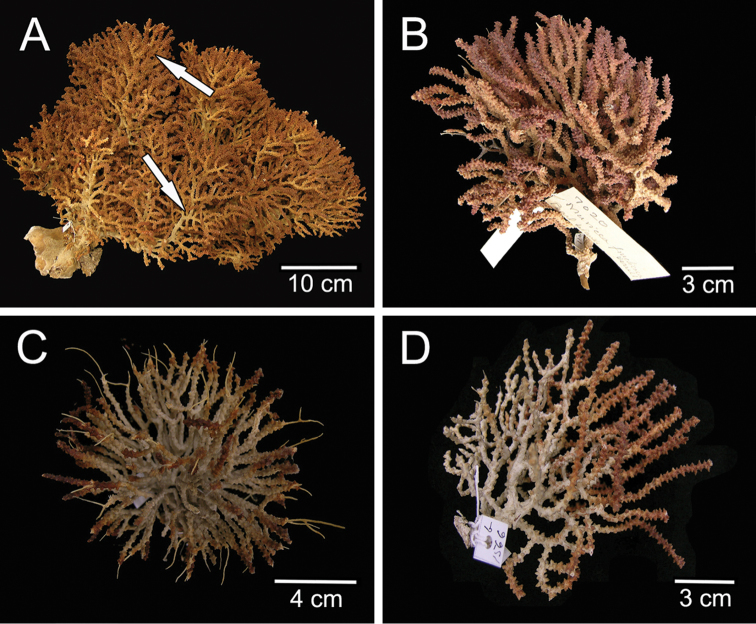
*Muricea fruticosa* Verrill, 1869 Morphological variation. **A** YPM 1574a, arrow showing two branch variations in the same colony **B** MCZ 7020 **C** YPM 1566d **D** YPM 1566b.

**Figure 4.**
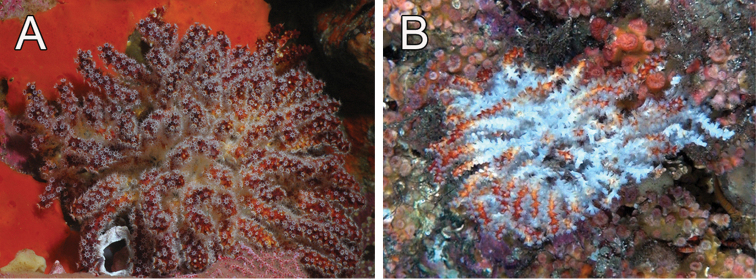
*Muricea fruticosa* Verrill, 1869. **A** *In situ* colonies, with expanded polyps, Nameless Islet, Galápagos Islands National Park, Ecuador. Photograph: Graham Edgar **B** *In situ* colonies, Everest mount, Isla del Coco National Park, 95 m deep. Photograph: *DeepSee* submersible.

Figures 1 , 2 , 3 , 4

Muricea fruticosa Verrill, 1869.
Muricea fruticosa Verrill, 1869: 428; Kükenthal 1919: 752; Kükenthal 1924: 142; Harden 1979: 147; Hardee and Wicksten 1996: 129.
Muricea fruticosa typica Kükenthal 1924: 142; Harden 1979: 147.
Muricea fruticosa var. miser Verrill, 1869: 430; Kükenthal 1919: 752; Kükenthal 1924: 143; Harden 1979: 149 (syn. n.).
Thesea crosslandi Hickson, 1928: 354–356 (syn. n.).
Pseudothesea crosslandi (Hickson, 1928); Stiasny 1943: 64–66 (syn. n.).

##### Material.

Lectotype (here designated). YPM 1574c, dry, Pearl Islands, Panamá, 11–14 m, F.H. Bradley, 1866. (YPM 1792, fragment from lectotype, Verrill’s 1868 figured specimen). Paralectotypes. PANAMÁ: MCZ 706 (fragment from YPM 1574); MCZ 7020; USNM 33588; YPM 1566 (as Muricea fruticosa var. miser), dry, Pearl Islands, F.H. Bradley, 1866, no more data; YPM 1660; YPM 1574a-b, d-e, same data as the lectotype; YPM 3067, dry, with the holotype of *Muricea retusa* at the base, Pearl Islands, 11–14 m, F.H. Bradley, 1866.

MCZ 5002; YPM 1566a-d; ZMUC-ANT 193 (as Muricea fruticosa var. miser), dry, Pearl Islands, F.H. Bradley, 1866. MCZ 4126 (*Parisis fruticosa*), ZMUC-ANT 169 (as *Thesea crosslandi*), ethanol preserved, San Jose Island, Pearl Islands, 49.3 m, T. Mortensen, 27 January 1916.

##### Description.

The lectotype is a large, bushy colony 35 cm tall, and about 45 cm wide. Four main branches, 25–35 mm in diameter, somewhat flattened, arise from an irregular, 52 mm diameter holdfast. The holdfast is spreading and raised about 30 mm above substrate, the specimen is attached to a plaster base for a past years museum display (Fig. 1A). The main branches subdivide very close to the base in secondary branches that immediately divide and subdivide in an irregular manner producing branches and branchlets closely placed, no more than 20 mm apart, at angles 45°–90°. Secondary branches and branchlets are 3–5 mm in diameter, mostly crooked and curved upwards or downwards. Some anastomosis occurs at the ends of branchlets. Unbranched terminal ends are 3–5 mm in diameter and 15–40 mm long. The axis is clear amber at the tips and darker at the base. The calyces are close together, or few millimetres apart, not imbricate, spreading outward and upward. They have large, strong, sharp sclerites forming the shelf-like projecting platform, 1–1.2 mm long, on the lower side (Fig. 1B). Polyps are on the upper side of the prominent calyces. The calyx sclerites give a prickly appearance to the colony (Fig. 1A–B). The calyx size and spacing vary from the larger branches to the thinner, being larger and acute, and closer placed at the branchlets and shorter, blunt, and distant at the main branches. The polyp apertures are covered by anthocodial sclerites. The coenenchyme is thin, composed of reddish-brown, amber, pale yellow to whitish sclerites (Fig. 1C–D). The outer coenenchyme and the calycular sclerites are composed of large, conspicuous unilateral spinous spindles visible to the naked eye (Fig. 2A–B). These spindles are of diverse shapes, with blunt or acute ends, or irregular with one acute end and the other blunt, with bifurcated ends or with spiny tips. The unilateral spinous spindles are basically spinulose on the outer surface and tuberculate on the inner surface in this species, some tubercles are large, sharp and spiny. The spindles are deep reddish brown, brownish yellow to pale yellow, and combinations of them (Fig. 1C). These spindles are 0.53–2.1 mm long, and 0.11–0.55 mm wide (Fig. 2A–B); they are forming the calyces and lying between them. The spindles bordering the calyx are long with stout, terminal spikes, 0.32–1 mm long and 0.07–0.2 mm wide, some with bifurcated warty ends (Fig. 2B). The axial sheath is composed of small, pale yellow to colourless spindles, 0.30–1 mm long and 0.05–0.12 mm wide, with whorls of small warts, and long spindles (Fig. 2C–D). Anthocodial sclerites are of a yellow to a very pale yellow colour, arranged in irregular points, mostly composed of warty spindles, 0.40–0.64 mm long, and 0.07–0.1 mm wide, small warty rods 0.2–0.38 mm long and 0.5–0.1 mm wide, and small branched spindles around 0.20 mm long, and 0.15 mm wide (Fig. 2E).

The colony is bicoloured, reddish brown at the tips, fading to a light yellow towards the base (Fig. 1A–B).

##### Habitat and variability.

There are two colour patterns in the syntype series, a marked bicoloured pattern, with dark reddish tips and whitish to pale yellow stems (Fig. 3C–D), and intermediate patterns less differentiated (Fig. 3B). The bicoloured pattern is more evident in small specimens than in large colonies (Figs 1A, 3A), and in some case there is a dominance of white colour in the branches. In large colonies, we have observed both patterns in different branches in the same colony (Fig. 3A, arrows). The lectotype (YPM 1574c) is the largest specimen and with the most profuse branching, some other colonies in the syntypes are formed by just a few branches. Our recent collected material shows the bicolour pattern and the colonies can reach up to 25 mm long and 30 mm wide. They are mostly bushy, openly ramified colonies with white polyps. Some specimens change colour after drying, the white part becomes a rusty reddish. When preserved in alcohol they keep the colours unaltered and do not tint the spirit. The sclerites of the paralectotypes and the other material analysed are in the variation range of the species. The calyces can be more sparsely set and shorter in some colonies. The species is found on rocky substrata in clusters or solitary, attached to small debris or shells, especially when sparsely distributed. The colonies are in caves or exposed to the currents. They are found in clear or turbid waters. *Muricea fruticosa* is found at various localities in the Galápagos Islands, exposed to moderate currents and in caves, reaching no more than 15 cm wide (Fig. 4A) (Hickman 2008, Breedy et al. 2009). The deepest record for the species is down to 102 m at Cocos Island seamounts (Fig. 4B), but it is found shallower in other places from 8 to 25 m deep.

##### Distribution.

From México to Perú. Type locality, Pearl Islands, Panamá.

##### Remarks.

Verrill (1869) described *Muricea fruticosa* with a collection of specimens from Panamá, and Muricea fruticosa var. miser based on some small specimens from the same locality but from shallower waters. He pointed out that the differences between the species and the variety are the small size of the colonies and the marked bicolour pattern. He also noticed that the calyces at the base of the branches were shorter, and that the sclerites were similar to the typical form but smaller. However, the reduction of the calyx size at the base of the branches occurs in most species of the genus, and the size of sclerites is in the range of variation found in the examined specimens. Hickson (1928) described *Thesea crosslandi* with specimens from Taboga Island, and San José Island (Pearl Islands), Panamá. Later, Stiasny (1943) re-examined Hickson specimens acknowledging the differences with the genus *Thesea* Duchassaing & Michelotti, 1864, and assigned the genus *Pseudothesea* Kükenthal, 1919 to the specimen. However, the description and the sclerite illustrations given by Hickson (1928), and Stiasny (1943) clearly refer to *Muricea fruticosa*, and we corroborated this after examination of Hickson’s specimens in the BM. For this reason, *Thesea crosslandi* and *Pseudothesea crosslandi* are herein treated as synonyms of *Muricea fruticosa*.

In order to establish the identity of this species, the YPM 1574c specimen is herein designated as the lectotype of *Muricea fruticosa*.

##### Other material revised.

COSTA RICA: UCR 482, 486, dry, Punta Conejo, Herradura, Puntarenas, 10 m, J. Cortés, 21 September 1996; UCR 520 (3), dry, Nicoya Gulf, CJ Kalb, 2 March 1967; UCR 576, dry, San Juanillo, Guanacaste, 12.5 m, J. Cortés, 14 June 1991; UCR 588, dry, Pitahaya Beach, Guanacaste, 20–23 m, J. Cortés, 15 June 1991; UCR 837-839, dry, Ballena Bay, Nicoya Gulf, 40 m, R/V Victor Hensen, 2 December 1993. ECUADOR: CASIZ 105032, ethanol preserved, Santa Cruz Island, Nameless Islet, Galápagos Islands, 20 m, P. Humann, no collection date. CDRS 03-76, ethanol preserved, Los Hermanos, Galápagos Islands, 9 m, C. Hickman, 17 January 2003. CDRS 06-33, ethanol preserved, Nameless Island, Galápagos Islands, 9–10 m, C. Hickman, 25 May 2006. IIN 7, 9, dry, Tambip, Salinas, 12–14 m, F. Rivera, P. Martínez, 20 July 2010; IIN 21, dry, Bajo Lunes, Salinas, 18 m, F. Rivera, P. Martínez, 21 July 2010; IIN 34, 69, Gigima, Salinas, 12–14 m, F. Rivera, P. Martínez, 22 July 2010; IIN 88, 89, 123, dry, Los Ahorcados, Machalilla National Park, 10–12 m, F. Rivera, P. Martínez, 25 July 2010. EL SALVADOR: UCR 1938, ethanol preserved, Departamento la Libertad, Playa Mizata, J. Segovia, 27 February 2010. MÉXICO: CASIZ 097734, ethanol preserved, Roca Partida, South side, Revillagigedo Islands, 36 m, R.J. Van Syoc, M/V “Royal Star, Clipperton Island Expedition 1994, 2 May 1994. CASIZ 103387, ethanol preserved, Boca del Tule to Arena Blanca, Baja California Sur, 30 m, W. Lee, J. Moran, J. McCosker, 26–27 April 1976; CASIZ 100843, ethanol preserved, Roca Alejos, Baja California Sur, 18–33, Robert Van Syoc, Cordell Expeditions, 5 November 1990. STRI 1124, 1130B, 1151, ethanol preserved, La Blanca, Oaxaca, 46–48 m, R. Abeytia, 23 August 2004. PANAMÁ: STRI 405, Seca Grande Island, 20 m, H. Guzman, 26 August 2002; STRI 534, Bajo Bolano, 25 m, H. Guzman, 16 April 2003; STRI 572, ethanol preserved, Viudas Island, 10–20 m, H. Guzman, 18 April 2003; STRI 836, San Telmo Island, 27 m, H. Guzman, 7 April 2004; STRI 848, Sur Pacheca, 2 m, H. Guzman, 20 April 2004; STRI 865, Achotines, 3–10 m, H. Guzman, 5 May 2004; STRI 879, Pearl Island, H. Guzman; STRI 888, Pearl Island, 25 m, H. Guzman, 15 August 2004; STRI 892, Pearl Island, H. Guzman, 15 August 2004; STRI 942, Pearl Island, 3–20 m, H. Guzman, 23 September 2004. USNM 34063, dry, Gulf of Panamá, L.C. Cash, no more data. PERÚ: CZA 230, dry, Foca Island, 12–15m, Y. Hooker, 14 June 2009; CZA 255; 293, dry, Punta Sal, 12–15m, Y. Hooker, 2 July 2011.

#### Muricea formosa

Taxon classificationAnimaliaAlcyonaceaPlexauridae

Verrill, 1869

Figures 5 , 6 , 7

Muricea formosa Verrill, 1869: 434–436; Kükenthal 1919: 752; Kükenthal 1924: 143; Harden 1979: 146.

##### Material.

Holotype. YPM 1621a, ethanol preserved, Zorritos, Tumbes Department, Perú, 5 m, F.H. Bradley, 1866–1867. Schyzotypes. PERÚ: MCZ 35945, 3 dry fragments, YPM 1621b, ethanol preserved fragment, Zorritos, 5 m, F.H. Bradley, 1866–1867.

##### Description.

**Figure 5.**
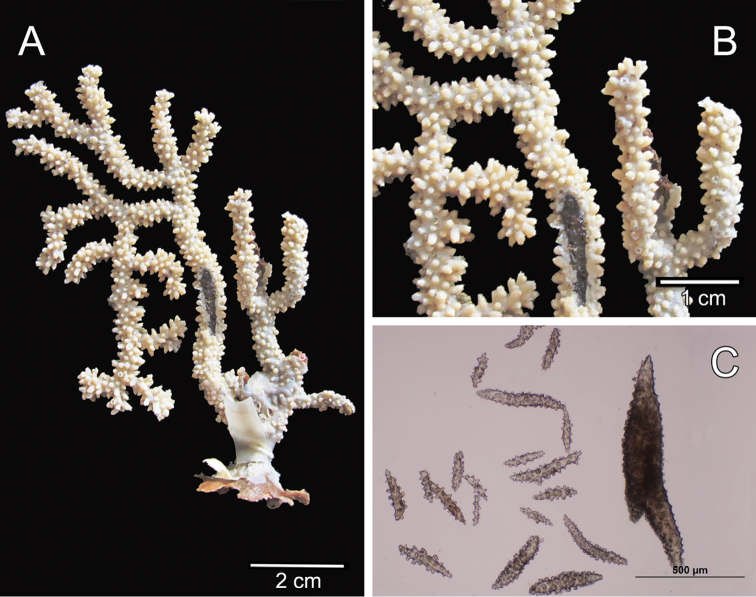
*Muricea formosa* Verrill, 1869 YPM 1574c. **A** Colony **B** Detail of branches **C** Sclerites, light micrographs.

**Figure 6.**
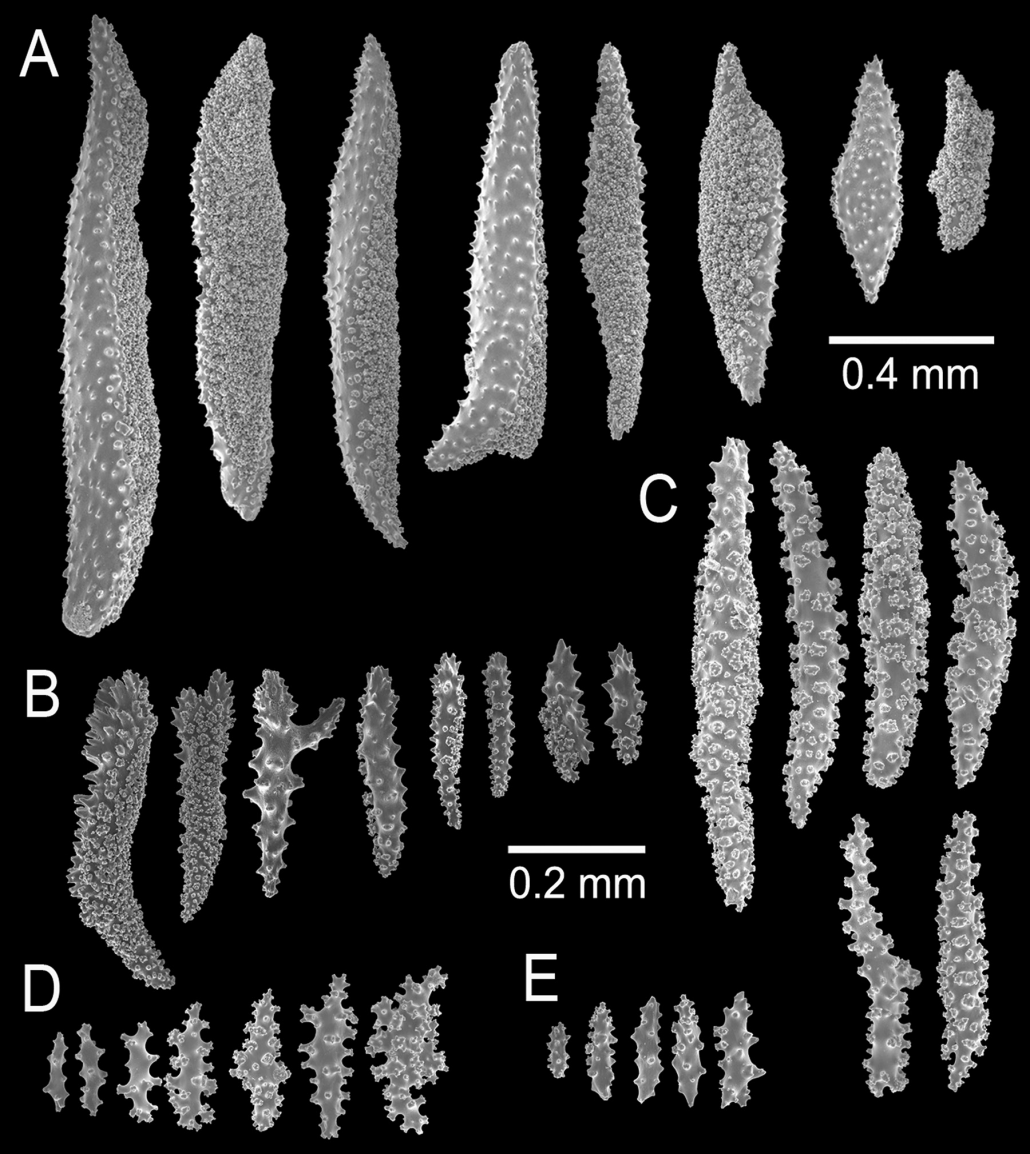
*Muricea formosa* Verrill, 1869 YPM 1574c. **A–C** Calycular and coenenchymal sclerites **D** Axial sheath sclerites **E** Anthocodial sclerites.

The holotype is a 10 cm long and 6 cm wide colony, branching lateral, in one plane and irregularly dichotomous (Fig. 5A). It arises from a conical holdfast about 100 mm tall and about the same in diameter. Two main branches bifurcate from a short stem, 7.5 mm in diameter, producing secondary branches that subdivide again at distances of 5–50 mm apart. The branches split at angles of 45° to 90°. The branches are 6–9 mm in diameter and little tapered toward the tips. The unbranched terminal ends are up to 28 mm long. The calyces are all around the branches, close together, not imbricate (Fig. 5B). They are elongated, sub-conical, projecting perpendicular to the branches, and directed upwards at smaller angles at the upper branches. The calyces reach up to 3 mm long with projecting spiny borders. The sclerites are all whitish to colourless (Fig. 5C). The outer coenenchyme and calycular sclerites are elongated with both ends sharp or with one end sharp and the other truncated or forked. They are unilateral spinous spindles, 0.33–1.5 mm long and 0.14–0.25 mm wide (Fig. 6A), the inner side with numerous small warts and the outer side with short spines. The spindles bordering the calyx are prickly spindles and modified leaf spindles, 0.3–0.475 mm long and 0.04–0.1 mm wide, with lateral or terminal spiny processes (Fig. 6B) that project beyond the calyx border, and elongated warty spindles, 0.41–0.7 mm long and 0.075–0.1 mm wide (Fig. 6C). The axial sheath is mostly composed of tuberculate, irregular spindles, 0.195–0.25 mm long and 0.084–0.13 mm wide (Fig. 6D). Anthocodial sclerites 0.086–0.175 mm long and 0.034–0.075 mm wide (Fig. 6E).

Colour of the colony is white.

##### Habitat and variability.

**Figure 7.**
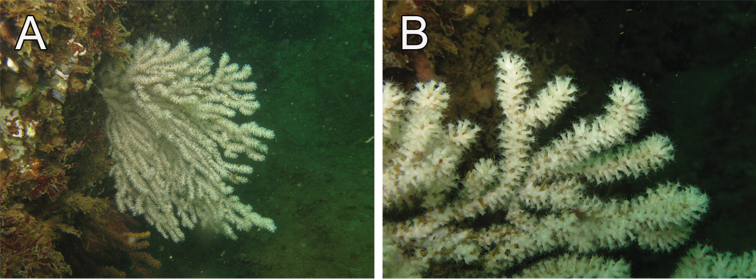
*Muricea formosa* Verrill, 1869. *In situ* colonies, Canoas de Punta Sal, Perú. Photograph: Yuri Hooker.

The species has been found living on rocky bottoms, caves and outcrops at 10–13 m in depth. The colonies are mostly growing in one plane, but in some cases they extend in two or three planes (Y. Hooker pers. comm.) (Fig. 7A). The examined colonies are bushy, mostly with lateral and irregular branching subdividing up to 10 times. Some branch anastomosis occurs. Colonies reach up to 25 cm long by 21 cm wide and bifurcate up to 10 times, diameter of branches reaches up to 10 mm. Sclerites are as in the holotype. The colonies are infested with a polychaete species that perforates the axes, and are also colonised in some branches by small cirripedia.

##### Distribution.

Reported from Las Ánimas Islet, Gulf of California by Harden (1979) and Canoas de Punta Sal, Perú (Y. Hooker pers. comm.), and Mazatlán, México (J.L. Carballo pers. comm.). Type locality, Zorritos, Perú.

##### Remarks.

Verrill (1869) described this species with a single specimen that was infested by a parasitic worm; this is consistent with the YPM 1621a specimen (Fig. 7A). All the recently collected material from Perú by Y. Hooker (2011–2012) is also hosting the same polychaete (Fig. 7A–B). We examined two small specimens from México that also show the tunnels and axial projections made by the worm.

##### Other material revised.

MÉXICO. M 18, dry, Punta Tiburón, Kino Bay, Sonora, 5.5 m, J.L. Carballo, 11 October 1999. PERÚ. CZA 286, dry, Canoas de Punta Sal, 12 m, Y. Hooker, 2 July 2011. CZA 412–416, 419, 424, dry, Canoas de Punta Sal, 13 m, Y. Hooker, 13 August 2012. CZA 286, Canoas de Punta Sal, 13 m, Y. Hooker, 2 July 2011. CZA 417–418, 420–423, Cabo Blanco, Piura, Y. Hooker, 13 August 2012. CZA 425, dry, El Ñuro, Piura, 10 m, Y. Hooker, 8 August 2012.

#### Muricea aspera

Taxon classificationAnimaliaAlcyonaceaPlexauridae

Verrill, 1869

Figures 8 , 9

Muricea aspera Verrill, 1869: 448–449; Kükenthal 1919: 752; Kükenthal 1924: 144–145; ?Harden 1979: 143.

##### Material.

Lectotype. YPM 1663A, dry fragment, Panamá, F.H. Bradley, 1866.

Paralectotypes. PANAMÁ: MCZ 35970; YPM 1663B-C, dry fragments, F.H. Bradley, 1866. YPM 1657, ethanol preserved, Pearl Islands, F.H. Bradley, 1866.

##### Description.

Type series is comprised of fragments, the larger ones are 12 cm tall and 9 cm wide, and 8.5 cm tall and 4.5 cm wide, probably fragments of a larger specimen (Verrill 1869).

**Figure 8.**
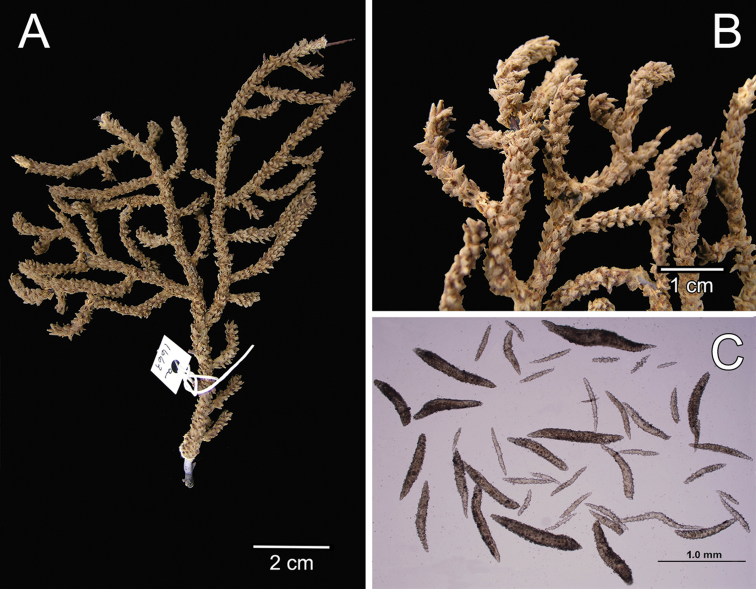
*Muricea aspera* Verrill, 1869 YPM 1663a. **A** Colony **B** Detail of branches **C** Sclerites, light micrograph.

**Figure 9.**
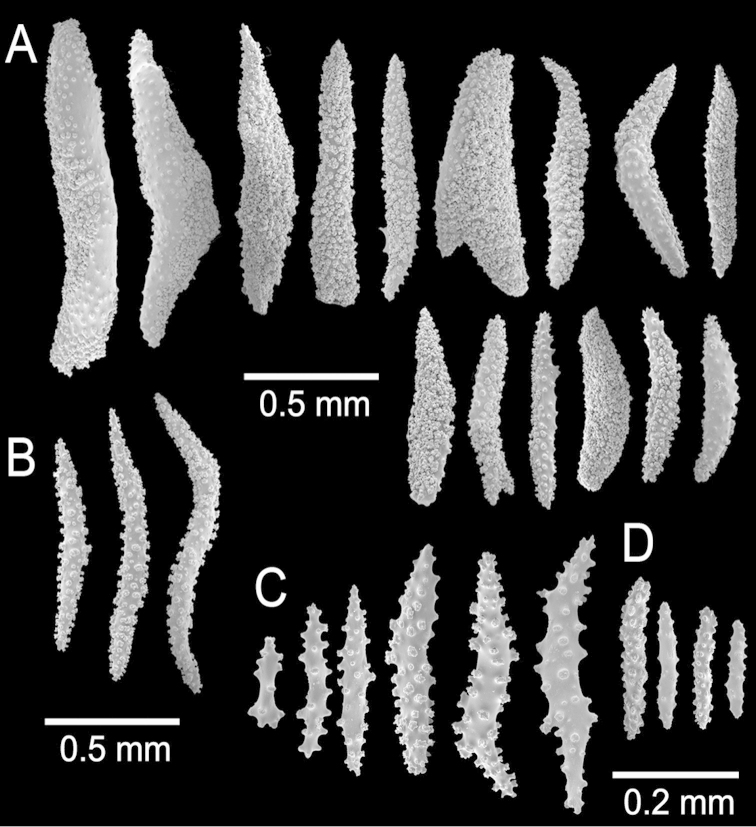
*Muricea aspera* Verrill, 1869 YPM 1663a. **A–B** Calycular and coenenchymal sclerites **C** Axial sheath **D** Anthocodial sclerites.

The lectotype is 12 cm tall and 9 cm wide (Fig. 8A), the branching is lateral and irregular; branches subdivide up to 5 times. Branches are 4 mm in diameter, they subdivide producing branchlets, 4–5 mm in diameter, closely placed, about 6–12 mm apart, mostly at angles of 45°, but some stick out at 90° angles. Unbranched terminal ends are 6–30 mm long. Calyces are prominent, 1–2 mm long from the base to the tip, with lower borders elongated, with straight tips or slightly curved inwards (Fig. 8B). Calyces are close together and slightly imbricate. Coenenchyme is thin, covered with long spindles. All sclerites are of a pale brown to whitish colour (Fig. 8C). The coenenchymal and calycular sclerites are mostly unilateral spinous spindles with one warty side and the other with sparse short spines. These spindles are 0.60–1.35mm long and 0.10–0.35 mm wide (Fig. 9A). Furthermore, elongated thin spindles are present, 0.80–1.15 mm long and 0.10–0.13 mm wide (Fig. 9B). The axial sheath is composed by irregular radiates and spindles, 0.14–0.40 mm long and 0.055–0.085 mm wide (Fig. 9C). Anthocodial sclerites are mostly flat warty rods, 0.15–0.25 mm long and 0.02–0.04 mm wide (Fig. 9D).

Colour of the colony is light brown.

##### Distribution.

Only reported for the type locality at extreme low waters (according to Verrill 1869). Type locality, Panamá.

##### Remarks.

Verrill (1869) described this species from two colony fragments that constitute the type series of *Muricea aspera*.

##### Other material revised.

PANAMÁ: STRI 559, ethanol preserved, Palito afuera Island, 5–8 m, H.M. Guzman, 17 April 2003.

#### Muricea echinata

Taxon classificationAnimaliaAlcyonaceaPlexauridae

Verrill, 1866

Figures 10 , 11

Muricea echinata Verrill, 1866: 328 (pars.); Verrill 1869: 426–427; Kükenthal 1919: 752; Kükenthal 1924: 143; Hickson 1928: 361–363.
Muricea echinata var. flabellum Verrill, 1869: 427–428; Kükenthal 1919: 752; Kükenthal 1924: 143.
Muricea echinata not Eunicea echinata Valenciennes, 1855: 13 (nom. nud.); Kükenthal 1924: 123.
Muricea echinata not Muricea echinata Milne Edwards & Haime, 1857: 143

##### Material.

Lectotype: YPM1565d, dry, Pearl Islands, Panamá, F.H. Bradley, 1866.

Paralectotypes: PANAMÁ: MCZ 67511, YPM 560 a-f, YPM 1565a,c-h, dry, Pearl Islands, F.H. Bradley, 1866.

##### Description.

**Figure 10.**
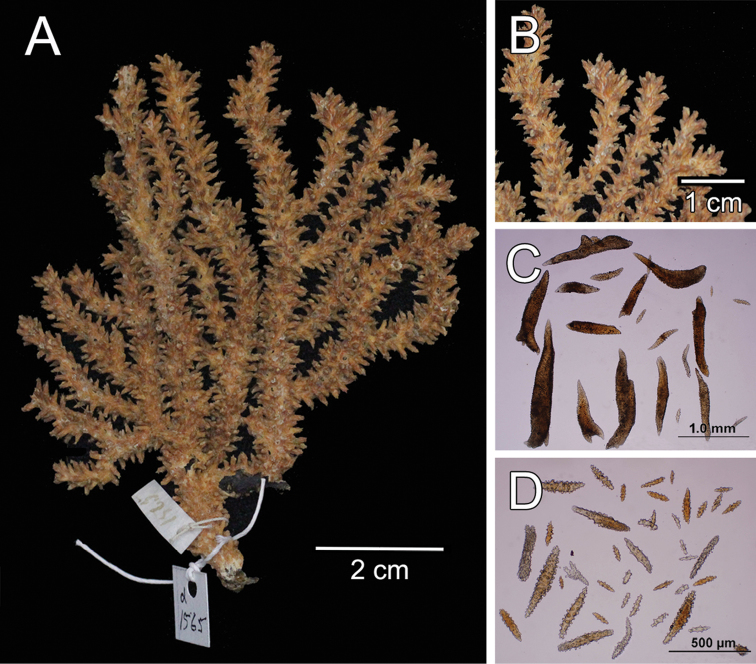
*Muricea echinata* Verrill, 1866. YPM 1565d. **A** Colony **B** Detail of branches **C–D** Sclerites, light micrographs.

**Figure 11.**
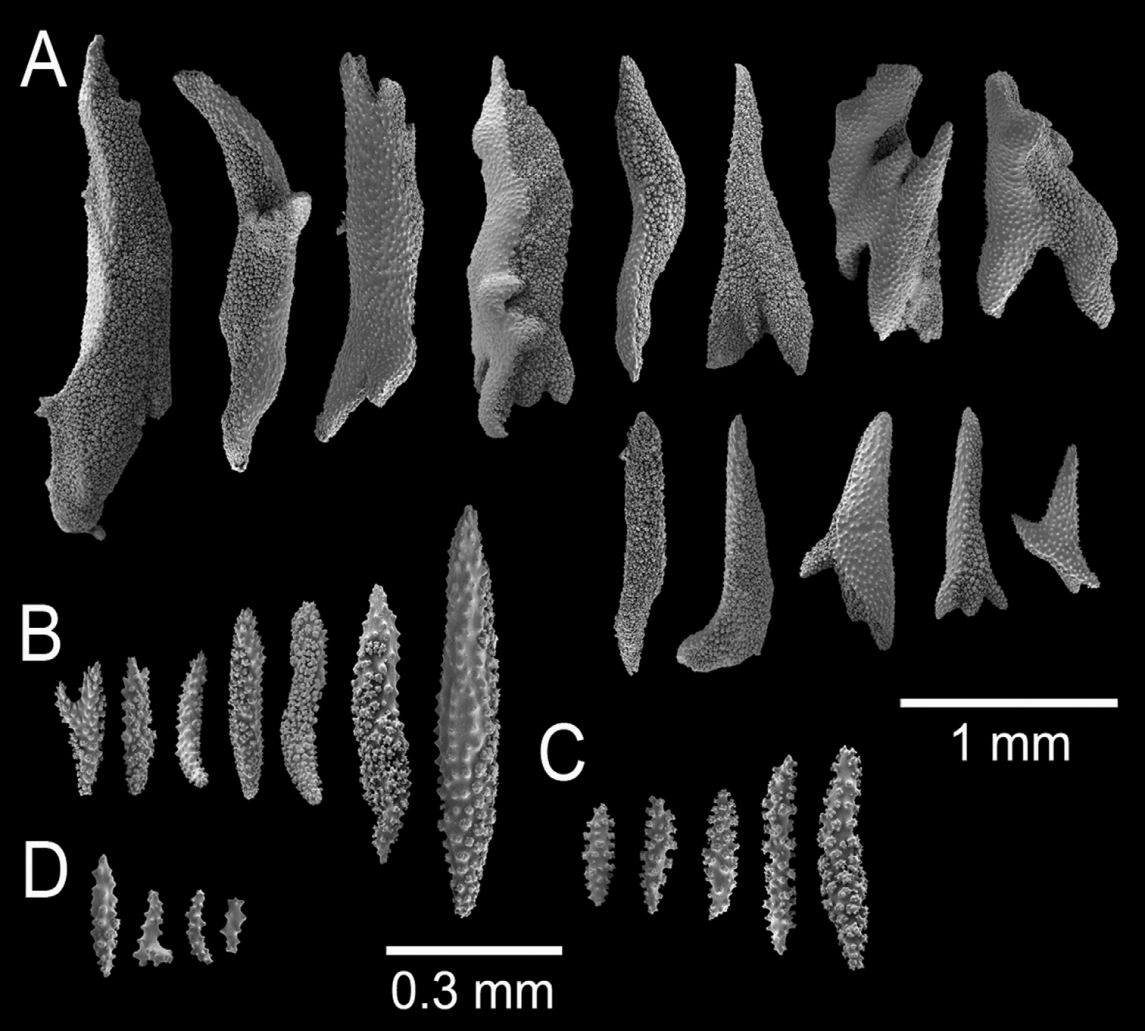
*Muricea echinata* Verrill, 1866. YPM 1565d. **A–B** Calycular and coenenchymal sclerites **C** Axial sheath sclerites **D** Anthocodial sclerites.

The lectotype is a colony 8.5 cm long and 7 cm wide, branching lateral and irregular and spreading in almost one plane (Fig. 10A). The main stem is 15 mm tall and 8 mm in diameter, it subdivides in three secondary branches, 5–7 mm in diameter, and then subdivides up to 6 times in an irregular manner producing subordinate branches, no more than 15 mm apart, at angles 35°–90°. These branches are of the same diameter, or thinner at the base and little thicker toward the tips, which are wide and clavate. The secondary branches are mostly crooked and curved upwards. About 1 cm oval portion of the holdfast remains. No anastomosis occurs. Unbranched terminal ends are 5–7 mm in diameter and 6–30 mm long. Axes are amber at the tips and darker at the base. Calyces are all around the branches, around 0.5 mm apart, not imbricate (Fig. 10B). They are prominent, up to 3 mm long, and 1–2 mm wide at the base, covered with large spindles with sharp ends, some of them project from the outer side (abaxial) elongating the lower border. The calyx size and spacing vary from the larger branches to the thinner, being larger and acute, and closer arranged at the upper branches and shorter and more distant at the lower or main branches. Polyps are small, on the upper (adaxial) side of the calyces. Coenenchyme is thin, outer coenenchyme is composed basically by the calyx sclerites, they are orange and light brown, the larger are darker (Fig. 10C–D). The outer coenenchymal and calycular spindles are of diverse shapes, unilateral spinous, spinulose on the outer surface and tuberculate on the inner, with conspicuous forms with bifurcated ends or cuspidate prolongations. These spindles are 1.55–2.4 mm long and 0.20–0.32 mm wide (Fig. 11A). Furthermore modified prickly-spindles are present, 0.35–0.6 mm long and 0.1–0.22 mm wide, with lateral thorny processes (Fig. 11B) and some with bifurcated ends. The axial sheath is composed of small, light brown warty spindles, 0.3–0.53 mm long and 0.09–0.13 mm wide (Figs 10D, 11C). Anthocodial sclerites are light brown rods and whitish-branched spindles (Fig. 11D), 0.07–0.2 mm long and 0.025–0.05 mm wide. They are longitudinally arranged at the base of the tentacles.

Colour of the colony is reddish brown.

##### Variability.

The examined colonies are up to 10 cm tall and up to 14 cm wide, branching irregular, spreading in almost one plane. Calyces can be up to 3.5 mm long. Colour of the colonies varies to a deeper orange hue.

##### Remarks.

*Muricea echinata* was erroneously mentioned by Verrill (1866) as *Muricea echinata* Valenciennes, 1855 (which was originally *Eunicea echinata*), with a minimal description. Later Verrill (1869) re-described the species with specimens from Pearl Islands, Panamá. We searched for the *Muricea echinata* type in the MNHN where Valenciennes’ specimens are kept, and found two specimens labeled as *Muricea echinata*: one, in the dry collection collected by Duchassaing in 1851 (that is the only information available), but only the wooden base of the specimen is left (S. Cairns pers. comm.); and the other collected by Agassiz in 1863, in the wet collection. None of these specimens could have been Valenciennes species because Duchassaing collections of *Eunicea* and *Muricea* species were from the Atlantic. Moreover, the wet specimen was collected after the species was named. Besides, there is no reference that could corroborate that Verrill analysed any of Valenciennes’ specimens. We conclude that Eunicea (Muricea) echinata Valenciennes was a *nomen nudum* or an inexistent species. Consequently, Verrill was the author of the species, producing the first description. Many different specimens have been assigned to this species in museum collections because its status was not clearly defined. We designate the specimen YPM 1565d as the lectotype to establish the taxonomic status of *Muricea echinata*. Verrill (1869) described Muricea echinata var. flabellum based on colonies with more branches, but in any other aspect this variety was consistent with the typical form. Therefore we consider it a synonym.

##### Other material revised.

COSTA RICA: USNM 44213, dry, Golfo de Nicoya, M Valerio, 20 February 1931. MÉXICO: USNM 42132, dry, Baja California, La Paz Bay, AL Herrera, 27 November 1919; USNM 57094, dry, Nayarit, West of Tepic, CR Orcutt, 30 August 1922. PANAMÁ: BM 1946.1.14.47; BM 1946.14.46; BM 30.6.17.17, dry, rock pools, low water mark, off Balboa, C. Crossland, 1914–1916. MNHN no catalogue number, ethanol preserved, M. Agassiz, 1863.

#### Muricea galapagensis

Taxon classificationAnimaliaAlcyonaceaPlexauridae

Deichmann, 1941

Figures 12 , 13

Muricea (?) galapagensis Deichmann, 1941: 6–9; Harden 1979: 150–151.

##### Material.

Holotype. USNM 43449, a colony and a fragment, Elizabeth Bay, Albemarle Island, Galapágos Islands, Ecuador, 91.4 m, W.L. Schmitt, Presidential Cruise, 26 July 1938.

##### Description.

**Figure 12.**
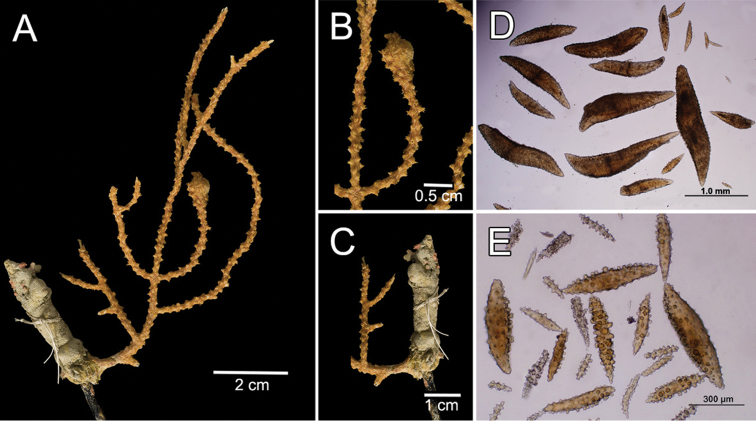
*Muricea galapagensis* Deichmann, 1941. USNM 43449. **A** Colony **B–C** Detail of branches, photographs: Walter Larrimore **D–E** Sclerites, light micrographs.

**Figure 13.**
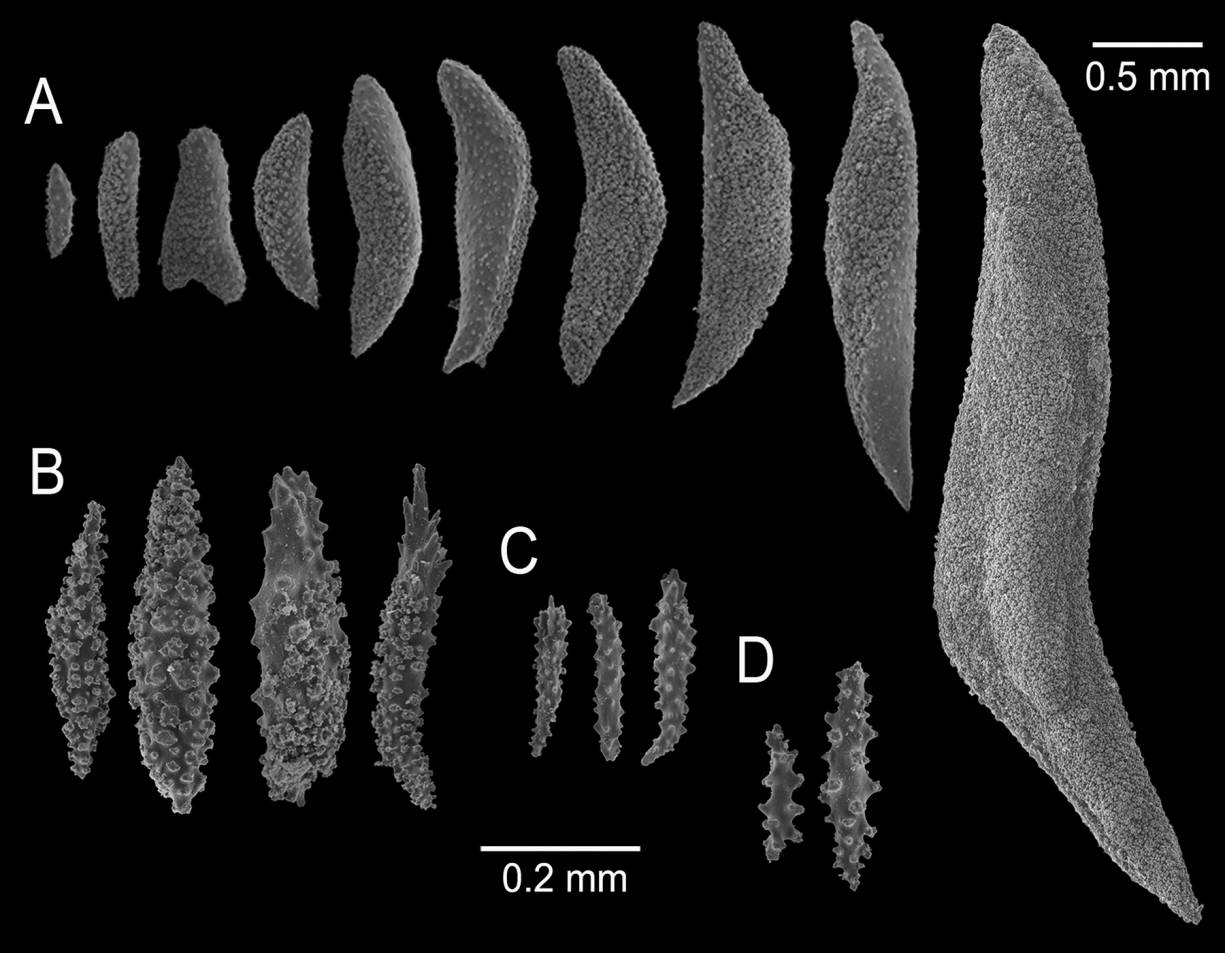
*Muricea galapagensis* Deichmann, 1941. USNM 43449. **A–B** Calycular and coenenchymal sclerites **C** Anthocodial sclerites **D** Axial sheath sclerites.

The holotype is a 10 cm tall and about 5 mm wide colony, it has one broken branch and according to Deichmann’s illustration (1941: 7, Fig. 2) the colony was wider and openly branched. The stem, 1 cm long and 0.3 cm in diameter, is attached to a black coral fragment by a conical holdfast about 1 cm in diameter. The stem subdivides (the lower branches are stumps) producing few long secondary branches sparsely placed, 3.7–29 mm apart, diverging at angles 30°–90° and curved upwards. Branches are 1.6–3 mm in diameter all along their length, and bifurcate up to 4 times (Fig. 12A). Unbranched terminal ends reach up to 80 mm long. The axis is clear amber at the tips and darker at the base. The calyces are low shelf-like, spreading outward and at right angles, no more than 1 mm long, not close or imbricate (Fig. 12B–C). The lower border is composed of 3-6 projecting spindles and the adaxial border, when present, of smaller spindles in an indistinct rim. The coenenchyme is thin, composed of amber to light orange sclerites (Fig. 12D–E). The outer coenenchyme and the calyces are composed of large, usually curved, unilateral spinous spindles that project beyond the calyx borders (Fig. 13A–B). They are visible to the naked eye. The largest size of sclerites in the genus is found in this species. These spindles are of diverse shapes, with blunt, acute or bifurcated ends, 0.43–4.1 mm long and 0.12–0.75 mm wide (Fig. 13A). Furthermore, warty spindles and prickly spindles are present, 0.35–0.45 mm long and 0.075–0.11 mm wide (Fig. 13B). The axial sheath is composed of warty spindles, 0.17–0.30 mm long and 0.04–0.08 mm wide (Fig. 13D). The polyp apertures are covered by pale orange and whitish anthocodial rods and small prickly spindles that were probably transversely placed at the base of the tentacles (Deichmann 1941). These rods and spindles are 0.2–0.25 mm long and 0.04–0.06 mm wide (Fig. 13C).

The colony is light orange (Fig. 12A–B).

##### Distribution.

Reported only from the type locality, Elizabeth Bay, Albemarle Island, Galapágos Islands, Ecuador.

##### Remarks.

Deichmann (1941) described this species with a specimen attached to a colony of *Antipathes galapagensis* Deichmann, 1941 that was pulled up with an anchor chain at the type locality. She reported another smaller and paler specimen in the MCZ (96.6 m, *Albatross* Sta. 3405). The specimen USNM 43449 represents the holotype.

### Species-group comparison summary

The *Muricea fruticosa* group comprises five species: *Muricea fruticosa*, *Muricea formosa*, *Muricea aspera*, *Muricea echinata* and *Muricea galapagensis*. This species-group is characterised by thin branches, long, prickly calyces and outer coenenchyme and calyces with unilateral spinous spindles (Tables 1–2). *Muricea fruticosa*, *Muricea echinata* and *Muricea galapagensis* have the largest spindles in the group, in the first they reach up to 2 mm long, the second 2.4 mm and the third 4 mm [Deichmann (1941) reported 2 mm long]. However, they are different in several characteristics; *Muricea fruticosa* has a profuse branching, mostly bicolored colony that separates it from the rest of the group. *Muricea galapagensis* differs from the others in having the thinnest branches and orange colonies. *Muricea formosa* differs from the others in that the colony and sclerites are white coloured and together with *Muricea echinata* have the most prominent calyces in the group (Table 2). It is comparable with *Muricea albida* (see *Muricea austera* group) which is also white with a similar colony form, but with thicker branches, shorter calyces, up to 2 mm long, which are more numerous and slightly imbricate. The outer coenenchymal sclerites in *Muricea albida* are wider (up to 0.40 mm) than those in *Muricea formosa* which are thinner (less than 0.05 mm) with acute ends (Table 1). The leaf-like spindles in *Muricea formosa* are not present in *Muricea albida*. In *Muricea albida* instead, there are modified warty spindles, shorter than in *Muricea formosa*, with a spiny end and a wider base (Figs 6, 30 below). *Muricea echinata* is similar to *Muricea aspera* and *Muricea fruticosa* by the prickly calyces but the longest are present in *Muricea echinata*, and the colour of the latter is reddish brown, darker than the light brown of *Muricea aspera* and different from the bicolour pattern of *Muricea fruticosa* (Tables 1–2).

### *Muricea plantaginea* group

#### Muricea plantaginea

Taxon classificationAnimaliaAlcyonaceaPlexauridae

(Valenciennes, 1846) comb. n.

**Figure 14.**
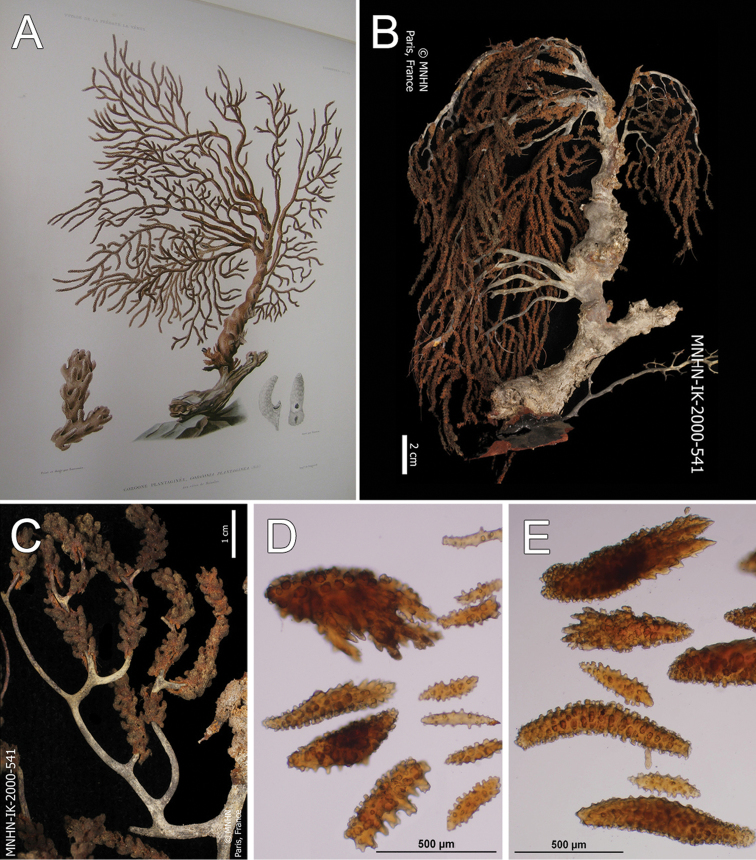
*Muricea plantaginea* (Valenciennes, 1846). MNHN oct 0541. **A** Original figure of the holotype, Valenciennes 1846: plate15 **B** Colony **C** Detail of branches, **B** and **C** photographs: Aude Andouche **D–E** Sclerites, light micrographs.

**Figure 15.**
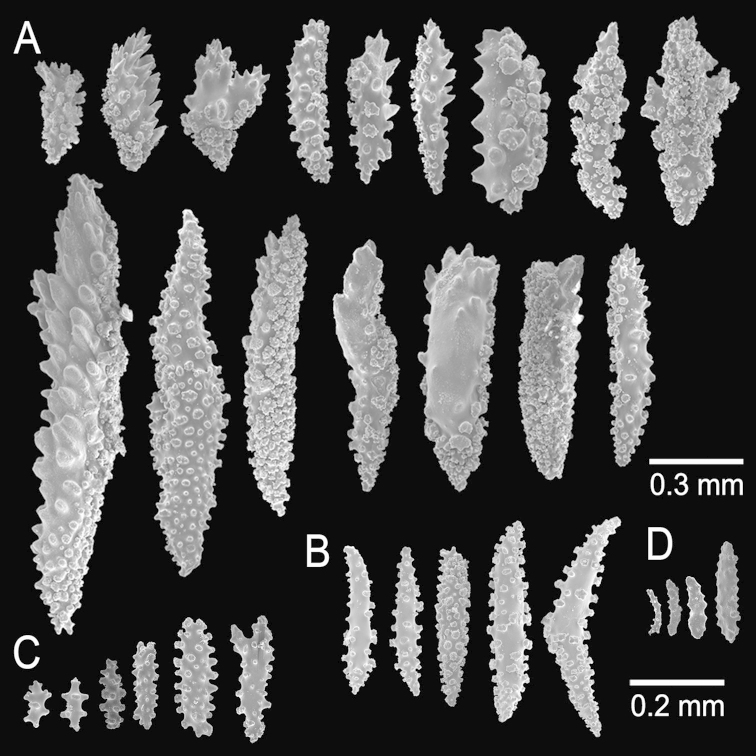
*Muricea plantaginea* (Valenciennes, 1846). MNHN oct 0541. **A–B** Calycular and coenenchymal sclerites **C** Axial sheath sclerites **D** Anthocodial sclerites.

**Figure 16.**
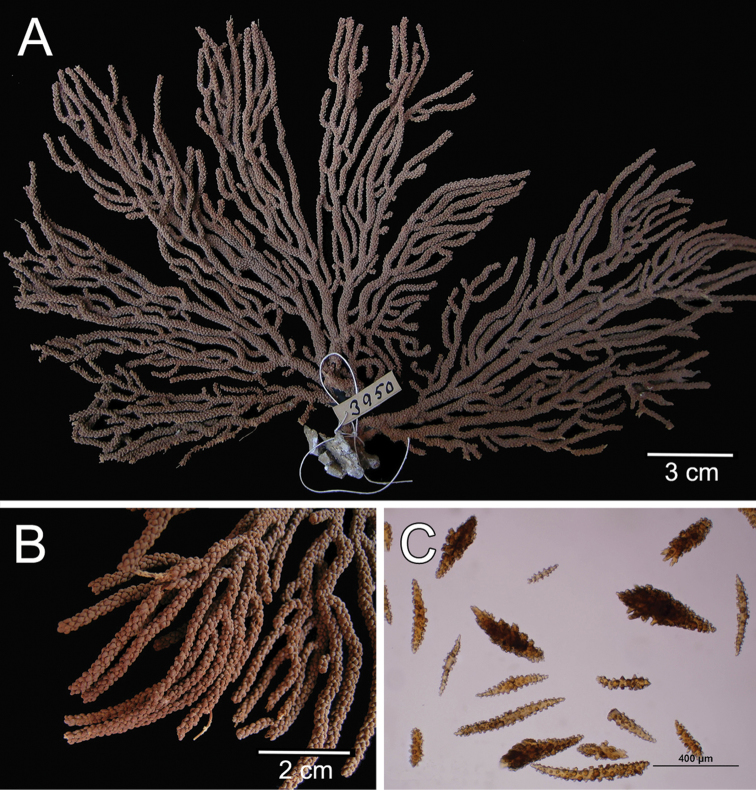
*Muricea plantaginea* (Valenciennes, 1846), MCZ 3950. **A** Colony **B** Detail of branches **C** Sclerites, light micrographs.

**Figure 17.**
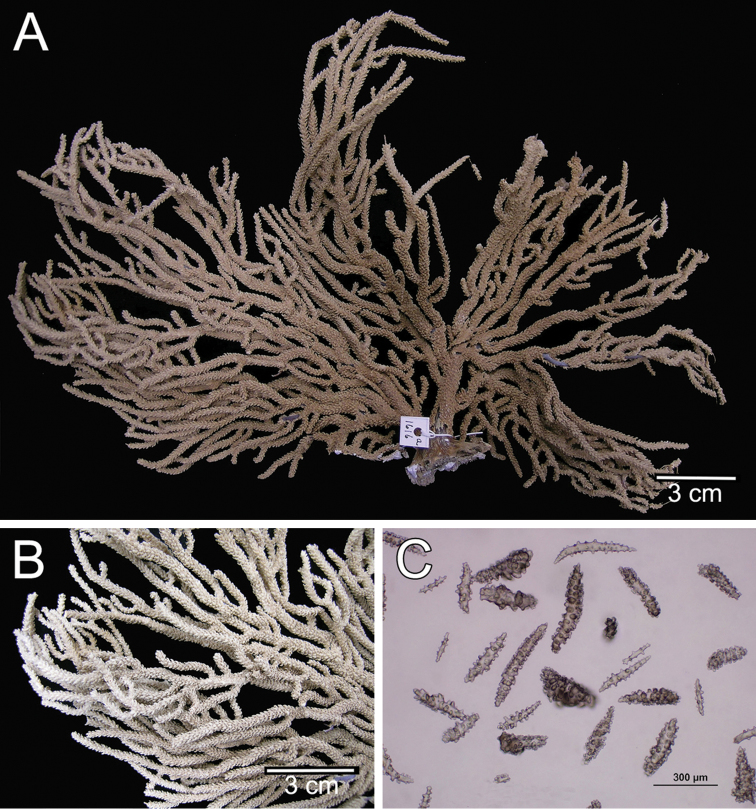
*Muricea plantaginea* (Valenciennes, 1846). YPM 1616A, Verrill syntype of Muricea appressa var. flavescens. **A** Colony **B** Detail of branches **C** Sclerites, light micrograph.

**Figure 18.**
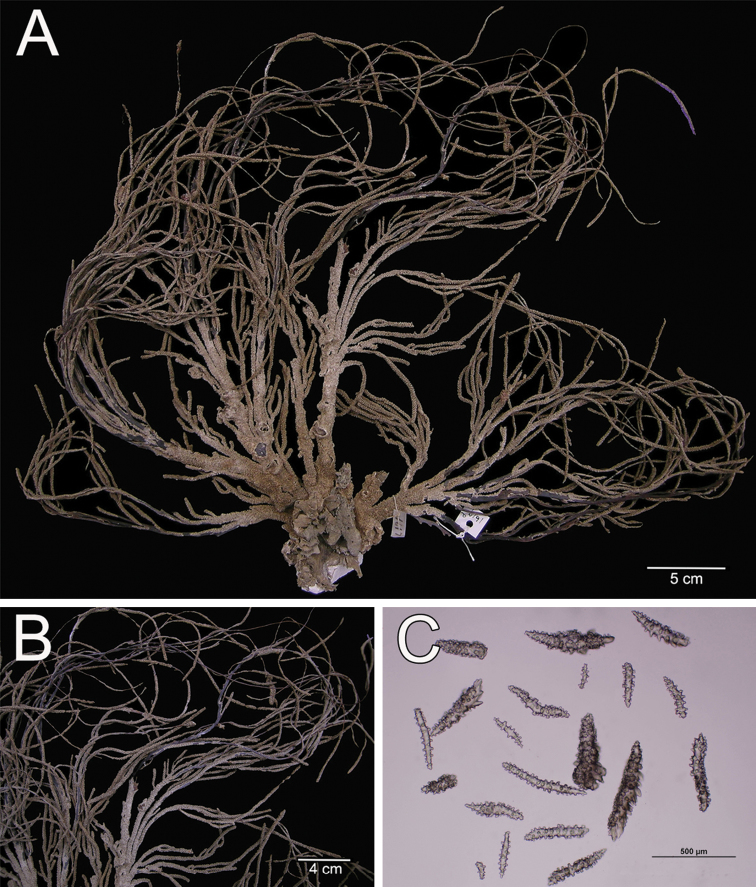
*Muricea plantaginea* (Valenciennes, 1846). YPM 1617A, Verrill syntype of *Muricea tenella*. **A** Colony **B** Detail of branch **C** Sclerites, light micrographs.

**Figure 19.**
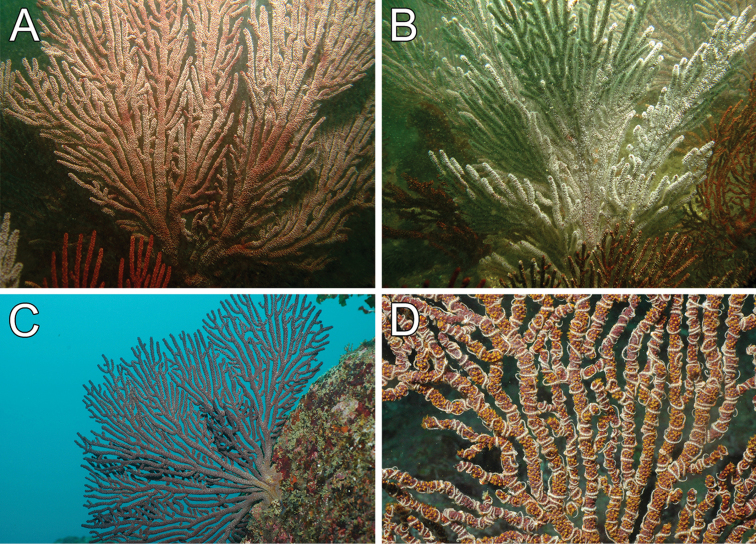
*Muricea plantaginea* (Valenciennes, 1846), colonies *in situ*, submarine pictures. **A–B** Bajo Lunes, Ecuador, photograph: Fernando Rivera **C–D** Albermarle, Galápagos Islands, Ecuador, photograph: Graham Edgar.

Figures 14 , 15 , 16 , 17 , 18 , 19

Gorgonia plantaginea Valenciennes, 1846: pl 15.
Muricea plantaginea not Gorgonia plantaginea Lamarck, 1815: 163 (Antilles).
Muricea plantaginea not Eunicea plantaginea Valenciennes, 1855: 13; Milne Edwards and Haime 1857: 151
Eunicea tabogensis Duchassaing & Michelotti, 1864: 17.
Muricea appressa Verrill, 1864: 37; Verrill 1866: 329; 1868: 412; 1869: 444–446; Kükenthal 1919: 752; Kükenthal 1924: 145; Riess 1929: 390–391; Hardee and Wicksten 1996: 132–136 (syn. n.).
Muricea appressa var. flavescens Verrill, 1869: 446; Kükenthal 1919: 752; Kükenthal 1924: 145 (syn. n.).
Muricea tenella Verrill, 1869: 446–448; Kükenthal 1919: 752; Kükenthal 1924: 145; Hickson 1928: 371–372; Riess 1929: 389–390; Stiasny 1943: 72–74; Harden 1979: 160 (syn. n.).

##### Material.

Holotype. MNHN oct 0541, dry, Mazatlán, Mexico, Voyage sur la Frégate La Vénus, M.A. Du Petit Thouars, 1836–1839.

##### Other type material.

*Muricea appressa*: MCZ 381–384, 3950 (380), 3950A-B, ethanol preserved, Panamá, P.H. Sternberg, July 1863. USNM 33585, 33587, 44162, dry, Panamá, J.H. Sternberg, no more data found. *Muricea appressa var. flabescens*: YPM 1616A, Zorritos, Perú, 5–9 m, F.H. Bradley, 1866–1867. MCZ 705; YPM 1179A-D, USNM1130760 (YPM 1616), dry, Pearl Islands, Gulf of Panamá, F.H. Bradley, 1866–1867. *Muricea tenella*: YPM 1617A-B MCZ4978 (708), dry, Zorritos, Perú, F.H. Bradley, 1867. YPM 1180B, dry, Pearl Islands, Panamá, F.H. Bradley, 1866. YPM 1657, ethanol preserved, Pearl Islands, F.H. Bradley, 1866.

##### Description.

The holotype is a large, flabelliform colony 40 cm tall and 18 cm wide. The colony is in bad shape, the main branches are almost nude and branchlets are bent to the sides (Fig. 14B). However, from Valenciennes´ illustration (Fig. 14A) it is possible to tell the flabelliform original aspect. A thick, 4.32 cm main branch arises from an irregular holdfast 5.29 cm in diameter, then bifurcates 6 cm above the base, producing two secondary branches 10–12 mm in diameter. One secondary branch is broken and the other subdivides many times into 4–3 mm wide branches which subdivide in an irregular manner producing branchlets 2–3 mm in diameter. Branchlets closely placed, about 10–15 mm apart, mostly at angles of 30°–45°. Unbranched terminal ends are 10–20 mm long. Calyces are small, 0.7–1 mm long from the base to the tip, with elongated lower borders curved inwards. Calyces are numerous, very close together and imbricate, 10–20/cm around the branchlets and more crowded and smaller at the branches. Coenenchyme is thin. Calycular and coenenchymal sclerites are reddish-brown and amber (Fig. 14D–E). Coenenchymal and calycular sclerites are mostly reddish-brown leaf-like spindles, 0.22–1.0 mm long and 0.09–0.20 mm wide (Fig. 15A), and amber elongated warty spindles 0.24–0.53 mm long and 0.06–0.10 mm wide (Fig. 15B). The axial sheath is composed of spindles, with single or bifurcated ends and radiates, 0.10–0.3 mm long and 0.05–0.09 mm wide (Fig. 15C). Anthocodial sclerites are lobed and warty rods, 0.06–0.23 mm long and 0.02–0.05 mm wide (Fig. 15D).

Colour of the colony is deep brown.

##### Habitat and variability.

Verrill´s type series is very consistent in all characters with respect to the Valenciennes’ holotype. For example, the specimen MCZ 3950 (Fig. 16A–C) shows the flabellate colony with imbricate calyces, and the types and colours of the sclerites (Fig. 16C) matching the holotype. Some colour variation was found in the syntypes of Muricea appressa var. flavescens and *Muricea tenella* that are herein considered synonyms of *Muricea plantaginea*. The colony colour varies from lighter hues of brown to yellowish or whitish as in the former Muricea apressa var. flavescens and *Muricea tenella* (Figs 17A–B, 18 A–B), and also sclerites colour is whitish in these varieties (Figs 17C, 18C). Variation in sclerite’s size was observed in some specimens respect to the holotype, the leaf-spindles could be shorter (about 0.50 mm) and the warty spindles longer (about 0.70 mm). In living colonies, the polyps can be white or yellow (Fig. 19B–D). Branches reach up to 15 mm in diameter and branchlets up to 5 mm. Colonies could have thinner branchlets (less than 2.5 mm diameter) and longer unbranched terminal ends up to 15 cm long (Fig. 18A). Calyces could reach up to 1.2 mm long, density around branches and branchlets could vary 8–22 calyces/cm. Calyces are imbricate especially at the branchlets, more scarcely imbricate at thick branches or stems.

The species is widespread throughout the central archipelago of the Galápagos (Breedy et al. 2009, Hickman 2008), and along the coast of Ecuador (Rivera and Martínez 2011). The species is abundant in Bajo Lunes (coast of Ecuador, 18–20 m deep) where they grow on a flat rocky bottom covered by sand and thin grain sediment. The thin grain sediment suspends in the water column producing high turbidity. In this locality we have found the largest colony sizes, up to 1.20–1.30 m tall and 1.5 m wide (Fig. 19A–B). In the Galápagos the colonies are of smaller size, around 0.5–0.6 by 0.5–0.70 m, and they are found deeper, down to 30 m on rocky bottoms and in clear water (Fig. 19C). Along the coast of Panamá the colonies do not reach more than a half-meter in size. The species reaches its deepest record, down to 65 m, at Hannibal Bank, off Panamá coast.

##### Distribution.

A widespread distribution, it has been reported from México to Perú, at a depth range from 10–65 m including the oceanic islands, Galápagos (Ecuador) and Revillagigedos (México). Type locality: Mazatlán, México.

##### Remarks.

The species appears for the first time in Valenciennes’ book (1846) *as Gorgonia plantaginea*, apart from the illustration (Plate XV) of this species there is no description. The specimen MNHN oct 0541 matches Valenciennes´ drawing (Fig. 14A–B). Verrill (1864) proposed the genus *Muricea* for this species, describing it as *Muricea appressa*, and properly described it in 1868, but overlooked the previous name that according to the ICZN has priority. Verrill (1869) also mentioned a variety of this species Muricea appressa var. flavescens based on some difference in colour and size of sclerites (Fig. 17). He also described *Muricea tenella* based on the slender branches, acute calyces and slender and sharp spindles (Verrill 1869). However, he made these observations from one specimen (YPM 1617A) that out of the long untidy branches, do not present significant difference to be considered another species.

##### Other material revised.

COSTA RICA: UCR 591, dry, Pitahaya Beach, Guanacaste, 23 m, J. Cortés, 15 June 1991; UCR 634, dry, Las Cocineras, Santa Elena Bay, Guanacaste, at the beach, O. Piedra B., 20 February 1965; UCR 945, dry, Caño Island, 22 m, H. Guzman, 11 February 1984. USNM 49389, dry, San Lucas, Gulf of Nicoya, Puntarenas, M. Valerio, January 1st 1930. ECUADOR: CDRS 04-302-304, ethanol preserved, Punta Albemarle, Galápagos Islands, 20–22 m, C. Hickman, 29 November 2004; CDRS 06-32, ethanol preserved, Nameless Island, Galápagos Islands, C. Hickman, 25 May 2006. IIN 23,24, 27a, 27, 28, dry, Bajo Lunes, Salinas, 18 m, F. Rivera, P. Martínez, 21 July 2010; IIN 30, 31, 40, 63-65, 72, dry, Gigima, Salinas, 12–14 m, F. Rivera, P. Martínez, 22 July 2010; IIN 92, 124, dry, Los Ahorcados, Machalilla National Park, 10–12 m, F. Rivera, P. Martínez, 25 July 2010.

MÉXICO: CASIZ 099631, 097735, ethanol preserved, Roca Partida, Revillagigedo Islands, 36 m, R.J. Van Syoc, M/V “Royal Star, Clipperton Island Expedition 1994, 2 May 1994. MNHN oct, dry, Baja California, M.L. Piquet, 1898, no more data. M-Gorgonia7, dry, Islas Gringas, San Carlos Bay, Sonora, 5–25 m, J.L. Carballo, 27 November 2002. PANAMÁ: STRI 557, etanol preserved, Palito Afuera Island, 5–8 m, H. Guzman, 17 April 2003; STRI 778, Pedro Gonzales Island, 3 m, H. Guzman, 11 August 2003; STRI 829, 831, 833, San Telmo Island, 27 m, H. Guzman, 7 April 2004; STRI 902, 903, 906, Pedro Gonzales Island, 10 m, H. Guzman, 23 September 2004. USNM 34065, dry, Gulf of Panamá, Panamá Bay, no more data found.

#### Muricea californica

Taxon classificationAnimaliaAlcyonaceaPlexauridae

Aurivillius, 1931

Figures 20 , 21

Muricea californica Aurivillius, 1931: 111–114; Harden 1979: 144–145; Hardee and Wicksten 1996: 130–132.

##### Material.

Lectotype. USA: SMNH 1122, ethanol preserved, Santa Catalina, California, 18–27.4 m, Leg. G. Eisen, 1874.

##### Description.

**Figure 20.**
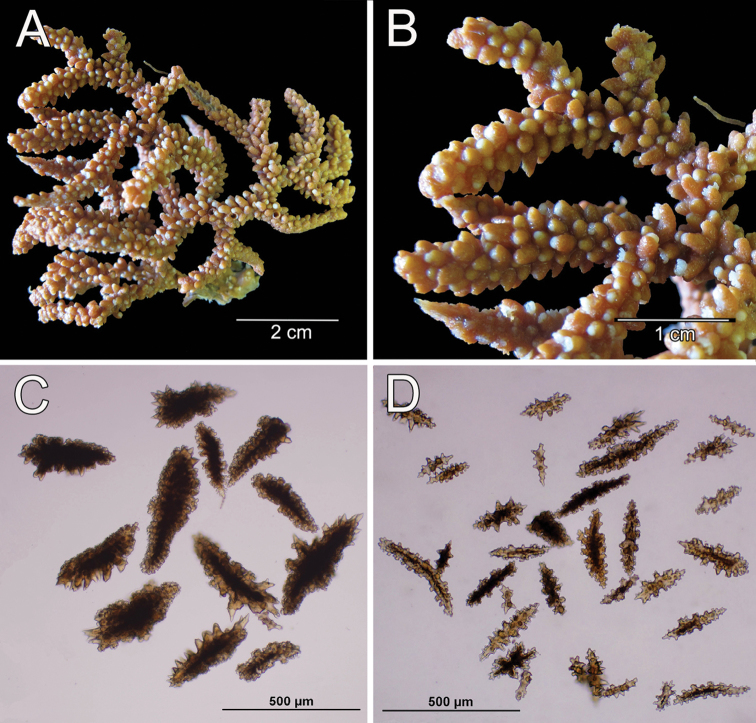
*Muricea californica* Aurivillius, 1931. SMNH 1122. **A** Colony **B** Detail of branches **C–D** Sclerites, light micrograph.

**Figure 21.**
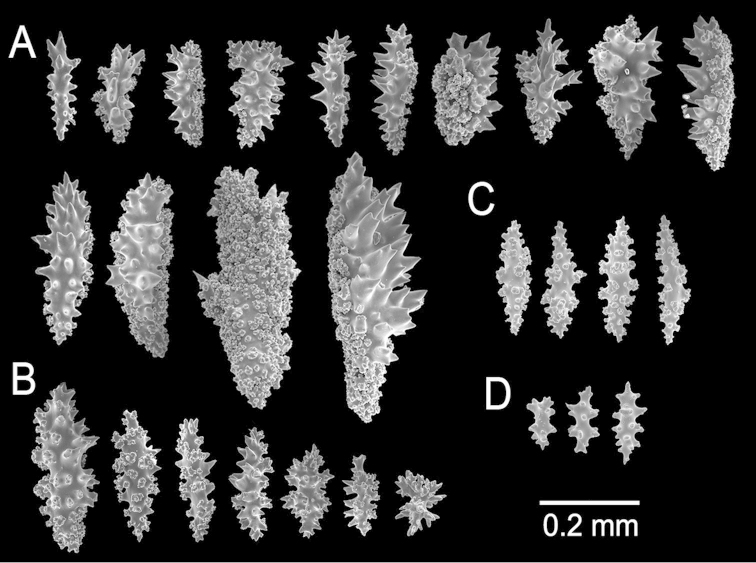
*Muricea californica* Aurivillius, 1931. SMNH 1122. **A–B** Calycular and coenenchymal sclerites **C–D** Axial sheath sclerites.

The lectotype is a bushy colony 5.6 cm tall and 7.8 cm wide. Two main branches, diameter 4.6–5 mm, arise from an oval holdfast, 1.38 cm in diameter (Fig. 20A). Branches are cylindrical, mostly in one plane, subdividing, and curving upwards parallel to the main branches. They are about 3–3.2 mm in diameter, of even thickness at the ends. Branching is lateral and irregularly dichotomous, dividing up to 8 times. Unbranched terminal ends are 0.5–2.8 mm long. The axis is amber at the base and lighter at tips. Calyces are closely set all around the branches, more distantly placed at the base of the colony and the holdfast. The calyces are elongate, 1.1–1.9 long and about 0.9–1.0 mm wide (up to 1.4 wide after Aurivillius 1931), extending upwards and slightly imbricate (Fig. 20B). The outer side of the calyces (abaxial) with numerous imbricate sclerites, and the adaxial with very few. Polyps are white (Fig. 20B). The coenenchyme is thin, composed of reddish-orange and light yellow to amber sclerites (Fig. 20C–D). Coenenchymal and calycular sclerites are mostly reddish-orange and amber leaf-like spindles, 0.19–0.54 mm long and 0.08–0.2 mm wide (Fig. 21A), and elongated spindles, 0.24–0.34 mm long and 0.07–0.09 mm wide (Fig. 21B). Aurivillius (1931) reported larger sclerite sizes in other specimens, up to 0.66 mm long and 0.3 mm wide, but we did not find this size either in the lectotype or in the other samples. The axial sheath is composed of spindles, with single or composed tubercles, and star-like radiates, 0.12–0.34 mm long and 0.07–0.13 mm wide (Fig. 21C), and small radiates and spindles 0.10–0.16 mm long and 0.05–0.07 mm wide (Fig. 21D). Anthocodial sclerites are lobed and warty rods, 0.08–0.23 mm long and 0.017–0.06 mm wide, light orange.

Colour of the colony is reddish orange.

##### Habitat and variability.

Hardee and Wicksten (1996) found colonies of *Muricea californica* with white polyps on one set of branches and yellow polyps on the other branches. They reported that most of the colonies in Catalina Island area have orange and yellow polyps instead of white. Most of the colonies were fan-shaped but they found some shrubby ones. Grigg (1972) observed that the branch diameter of *Muricea californica* could vary according to exposure to current flow.

According to Hardee and Wicksten (1996) colonies of *Muricea californica* were found living at the lowest intertidal level on the Los Angeles Harbor Breakwater at San Pedro reaching down to 30 m along Santa Catalina Island. The colonies were found on granite and other hard rocks, shale reefs, pilings or attached to shells. Grigg (1972, 1977) reported the coexistence of *Muricea fruticosa* and *Muricea californica* off La Jolla, California. He reported that *Muricea californica* grew exposed on outer surfaces on rocks and hole borders while *Muricea fruticosa* was in the interior of holes or growing on the lower surface of overhangs. We have observed the same type of habitat for *Muricea fruticosa* in the Galápagos Islands, Panamá and Costa Rica, but we have not observed *Muricea californica* in these southern areas.

##### Distribution.

South of point Conception, California to Santa María, Baja California, Mexico (Grigg 1977); Santa Catalina, California (Aurivillius 1931, Hardee and Wicksten 1996); Isla Tiburón, Kino Bay, Sonora, Mexico (J.L. Carballo, pers. comm.).

##### Remarks.

The species was originally described by Aurivillius (1931) with two specimens from Santa Catalina, California. He made reference to other analysed material and provided a general diagnosis but he did not designate a holotype. The specimen SMNH 1122 is herein designated as the lectotype of *Muricea californica* in order to clearly establish the taxonomic status of the species.

##### Other material revised.

MÉXICO: Geoff1, dry, Baja California, Geoff Shester, 2007. Gorgonia 11, dry, Isla Tiburón, San Carlos Bay, Sonora, 5–25 m, J.L. Carballo, 27 April 2001. PANAMÁ: BM 30.6.17.18 (fragment), (erroneously identified as Muricea hebes), ethanol preserved, off Panamá, low tide, St. George, Scientific Expedition, Pacific Cruise, C. Crossland, 1923–1924.

#### Muricea mortensenii

Taxon classificationAnimaliaAlcyonaceaPlexauridae

Hickson, 1928

Figures 22 , 23

Muricea mortensenii Hickson, 1928: 369–371; Stiasny 1943: 69–72.

##### Material.

Holotype. MZUC ANT 106, ethanol preserved, Rey Island, Pearl Islands, Panamá, T. Mortensen, 27.4 m, 26 January 1916. Schizotype RMNH Coel 6553.

##### Description

**Figure 22.**
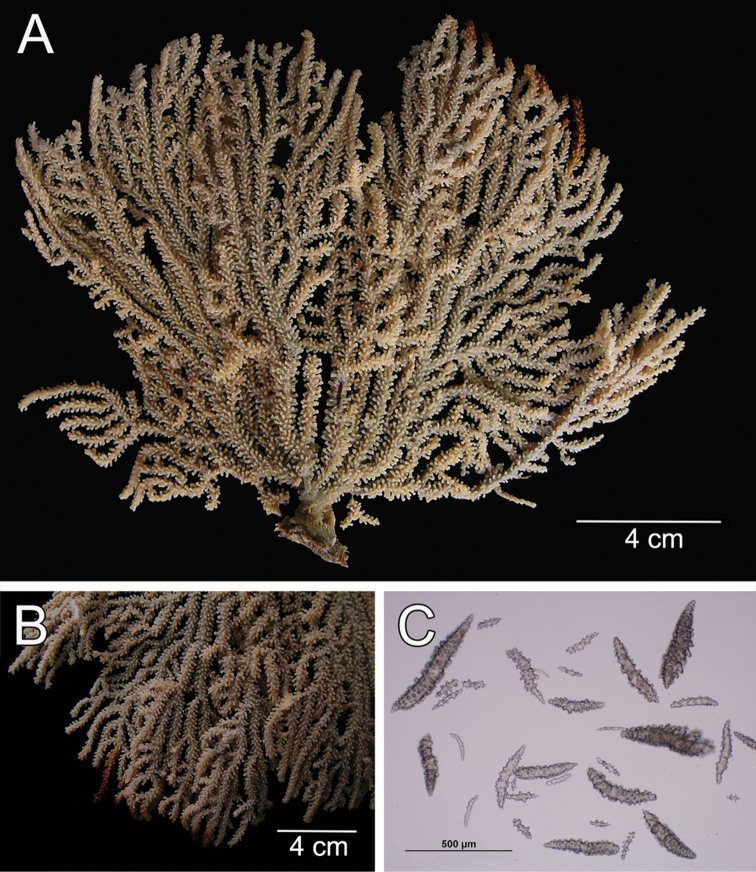
*Muricea mortensenii* Hickson, 1928. MZUC ANT 106. **A** Colony **B** Detail of branches **C** Sclerites, light micrograph.

**Figure 23.**
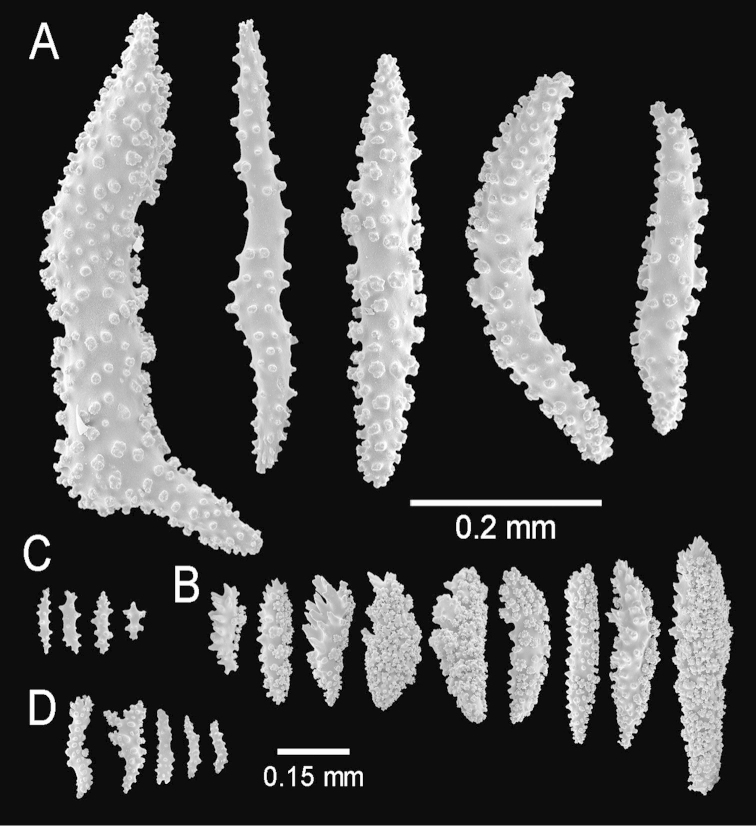
*Muricea mortensenii* Hickson, 1928. MZUC ANT 106. **A–B** Calycular and coenenchymal sclerites **C** Axial sheath sclerites **D** Anthocodial sclerites.

(see also Hickson 1928). The holotype is a flabelliform colony 15 cm tall and 18 cm wide (Fig. 22A). A short stem, 6 mm in diameter, arises from an irregular holdfast, 22 mm in diameter, which bears a few scattered polyps. The stem divides into numerous branches, each about 3 mm in diameter; they subdivide in an irregular manner producing branchlets, 2–3 mm in diameter, closely placed, mostly 1–2 mm apart, and at angles 30–45°. Branchlets are very numerous and overlap on the surface of the fan, but do not anastomose. Branches subdivide up to four times. Unbranched terminal ends are mostly 2–4 mm long. Calyces are elongated, 0.7–1.0 mm long from the base to the tip, with lower borders slightly curved inwards. Calyces are numerous, not imbricate, placed all around the branches, very close together, about 18–20 calyces/cm around the branches, 10–15/cm around the branchlets, (Fig. 22B). Polyps are whitish preserved in ethanol. All sclerites are whitish to colourless (Fig. 22C). Coenenchyme is very thin, composed of warty spindles, 0.35–0.7 mm long and 0.07–0.12 mm wide, of diverse shapes, straight, curved or irregular with one side curved and the other straight (Fig. 23A). Leaf spindles and modified forms 0.18–0.53 mm long and 0.068–0.13 mm wide, with long lateral sharp spines, are also present (Fig. 23B). The axial sheath is composed of immature forms, spindles and capstans, 0.08–0.13 mm long and 0.03–0.05 mm wide (Fig. 23C). Anthocodial sclerites are warty rods, 0.11–0.21 mm long and 0.02–0.08 mm wide, some with bifurcated ends (Fig. 23D).

Colour of the colony is pale yellow.

##### Distribution.

Reported for the type locality, Rey Island, Pearl Islands, Panamá.

##### Remarks.

Hickson (1928) described this species with one colony from Panamá. He indicated that the colour of the colony was pale umber brown, darker than the holotype which is pale yellow; we assumed that the colony was decoloured when preserved. The species is different from the others found in the area and we have not collected this species in any of our recent surveys in Panamá. According to Hickson 1928 this species is closely related to *Muricea hispida* Verrill, 1866 and *Muricea horrida* Möbius, 1861; however, the characteristic tubular calyces of these species separate *Muricea mortensenii*, which has shelf-like calyces. The species can be placed in the same group together with *Muricea californica* and *Muricea plantaginea* based on the lack of unilateral spinous spindles as structural part of coenenchyme and calyces, the thin branches, and the fan-shaped colony that have one or more planes. The holotype is the only colony presently found of *Muricea mortensenii*.

### Species-group comparison summary

The *Muricea plantaginea* group comprises three species: *Muricea plantaginea*, *Muricea mortensenii* and *Muricea californica*. The group is characterised by thin coenenchyme, thin branches and the lack of unilateral spinous spindles (as defined for the genus). The main components of the calyces and outer coenenchyme are leaf-like spindles with prominent spines and elongated warty spindles. *Muricea mortensenii* is conspicuously different from the others in the profuse branching, very crowded upwards-curved calyces and soft texture of the colony. *Muricea californica* and *Muricea plantaginea* (typical) have similar colony and sclerites colours. Respect to the colony morphology, *Muricea californica* is not as fan-shaped as *Muricea plantaginea*. Colonies could develop in various planes. *Muricea californica* differs also from *Muricea plantaginea* in having shorter spindles, no more than 0.55 mm long, and longer, not imbricate calyces (Tables 1–2).

### *Muricea austera* group

#### Muricea austera

Taxon classificationAnimaliaAlcyonaceaPlexauridae

Verrill, 1869

**Figure 24.**
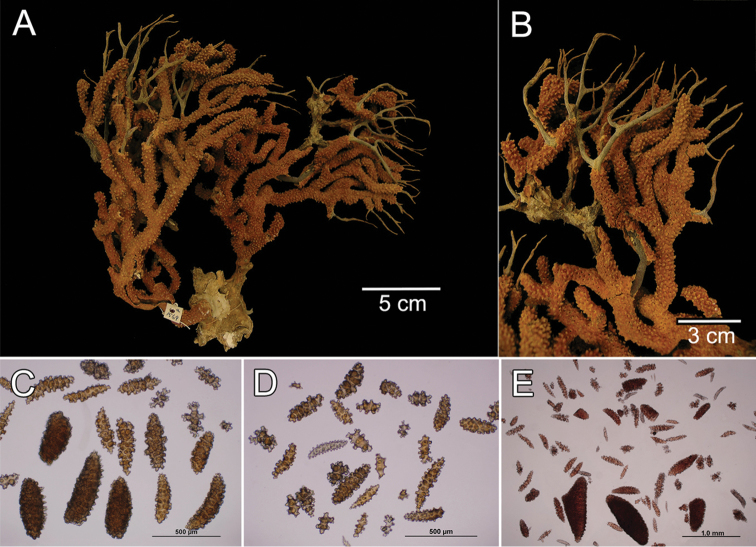
*Muricea austera* Verrill, 1869. YPM 1569a. **A** Colony **B** Detail of branches **C–E** Sclerites, light micrographs.

**Figure 25.**
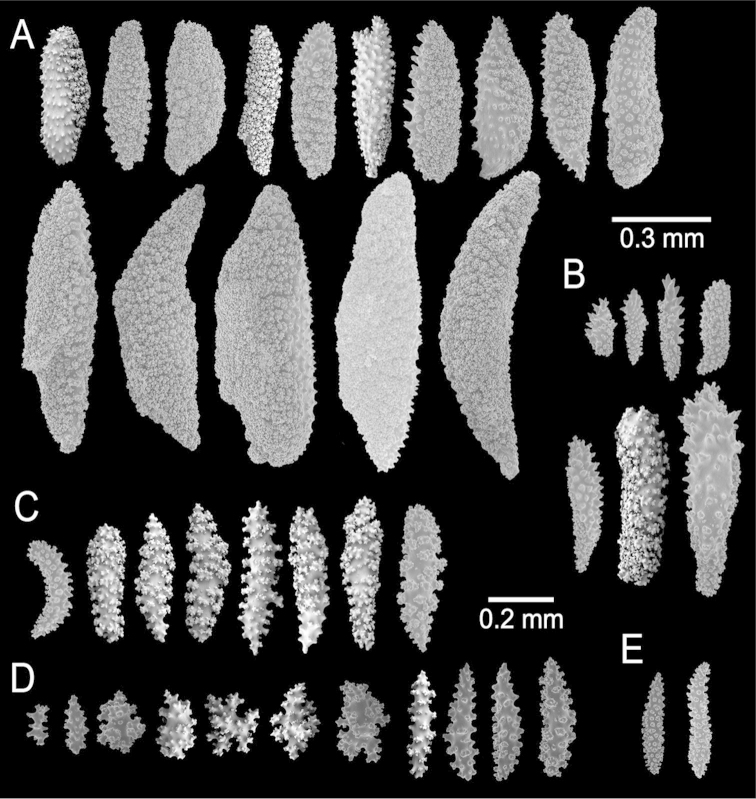
*Muricea austera* Verrill, 1869. YPM 1569a. **A–C** Calycular and coenenchymal sclerites **D** Axial sheath sclerites **E** Anthocodial sclerites.

**Figure 26.**
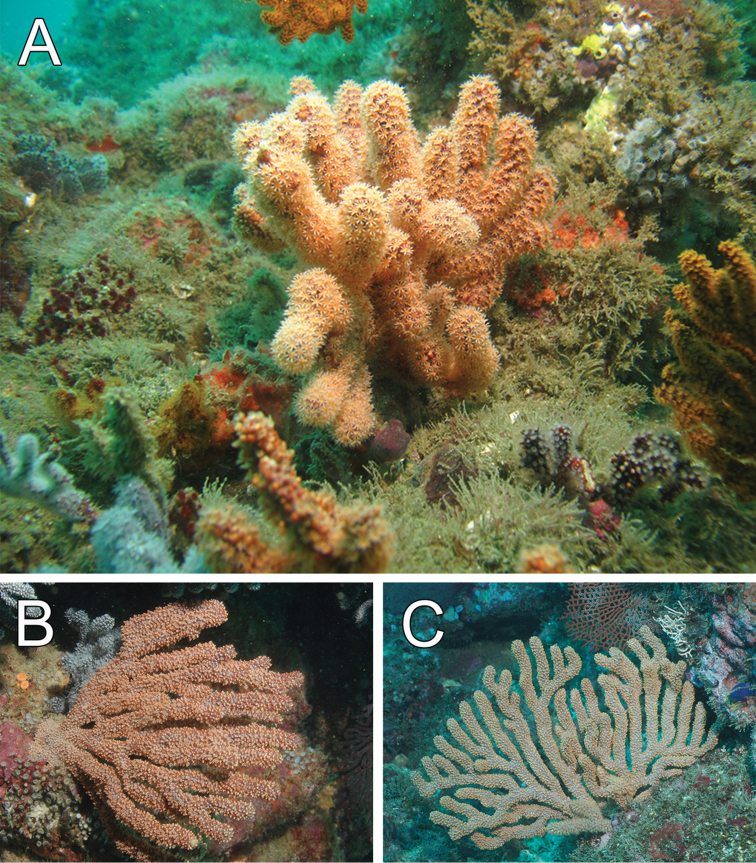
*Muricea austera* Verrill, 1869, colonies *in situ*, submarine pictures. **A** Gigima, AMP Puntilla de Santa Elena, Ecuador, photograph: Fernando Rivera **B** Salango, Machalilla National Park, Ecuador, photograph: Fernando Rivera **C** Coiba National Park, Panamá, photograph: Graham Edgar,.

Figures 24 , 25 , 26

Muricea austera Verrill, 1869: 430–432; Kükenthal 1919: 752; Kükenthal 1924: 142; Harden 1979: 143–1 44; Hickson 1928: 367–369.

##### Material.

Lectotype: YPM 1569a, dry, Pearl Islands, Panamá, 11–14 m, F.H. Bradley, 1866. Paralectotypes: MÉXICO: MCZ 4974; USNM 3094; dry, Cape San Lucas, Baja California, J. Xantus, April 1859-August 1861. USNM 52291; USNM 1130762 (part of YPM 8660); YPM 8660, dry, La Paz, Baja California, J. Pedersen, no date. PANAMÁ: USNM 1130761 (part of YPM 1569a); YPM809a-c; YPM 1569b; ZMUC-ANT 191 (part of YPM 809), dry, Pearl Islands, 11–14 m, F.H. Bradley, 1866.

##### Description.

The lectotype is a bushy colony, 20 cm long and 23 cm wide, with brittle coenenchyme that is partially lost on some branches and with mostly naked terminal ends (Fig. 24A). Two slightly flattened stems, each 7–10 mm in diameter, arise from a large holdfast, about 6 cm long, devoid of coenenchyme and with a small white sponge attached (Fig. 24A). The branching is mostly dichotomous and mostly in one plane (Fig. 24A). The stems extend up to 15 mm long and subdivide into secondary branches, that bifurcate up to 6 times producing subordinate branches, no more than 25 mm apart. Some branches are wider at the lower part of the colony, up to 10 mm in diameter, but they are mostly of even thickness up to the tips, 7–8 mm in diameter. The branches bifurcate at close angles 30°–45°, and curved upwards. Some occasional branch anastomosis occurs, especially at the base. Unbranched terminal ends are up to 5 cm long, with rounded tips, and 6–9 mm in diameter. Axes are brownish and lighter at the tips. The calyces are all around the branches, bent upwards and close together, not imbricate but a little overlapped (Fig. 24B). They are raised, up to 2 mm long. The coenenchyme is thick and rough, composed of reddish–brown, orange and light yellow sclerites (Fig. 24C–E). The outer coenenchymal and calycular spindles are of various types: mostly unilateral spinous with the inner side heavily warty, and the outer side with short spines. They are elongated, with round ends, or with one end tapered and the other wide and blunt, or with one end acute or bifurcated, or tapered at both ends, 0.55–1.5 mm long and 0.20–0.50 mm wide (Fig. 25A). The largest sclerites are of a darker colour in the central part with lighter hues around the borders. Furthermore, leaf-like spindles are present, 0.22–0.65 mm long and 0.06–0.20 mm wide (Fig. 25B), with a spiny end that projects beyond the calyx border, and warty spindles, 0.35–0.48 mm long and 0.11–0.14 mm wide (Fig. 25C). The axial sheath is composed of warty radiates, 0.12–0.36 mm long and 0.12–0.2 mm wide, and spindles (Fig. 25D). Anthocodial sclerites are pale yellow to whitish, 0.14–0.36 mm long and 0.02–0.06 mm wide (Figs 24E, 25E).

Colour of the colony is reddish brown.

##### Habitat and variability.

The colonies ramify producing a bouquet-like structure (Fig. 26A) or mostly grow in one plane (Fig. 26B–C), in some cases, the holdfast extends for some distance along the substrate producing many more branches in a fringing-like colony that in some cases is growing upside down. The polyps are orange to yellow (Fig. 26A–B). In the examined specimens the larger sclerites reach up to 0.2 mm long. The branches vary in diameter from 6–10 mm, in some cases few bifurcations occur forming candelabrum-like colonies or single finger-like short branches joint by extending holdfast.

##### Distribution.

It has a widespread distribution, from México to Perú. Type locality: Pearl Islands, Panamá.

##### Remarks.

Verrill (1868) described this species with specimens from Pearl Islands, Cape San Lucas and La Paz, Baja California. Later, Hickson (1928) revised a specimen from Taboguilla Island, Gulf of Panama and added some details about variation in calyx length (little larger) and found larger sclerites, up to 2 mm long. Verrill (1868) and Hickson (1928) considered the species as rare in Panamá, but we found it common at various localities around Pearl Islands and Gulf of Chiriquí.

Verrill did not designate a holotype for the species. The specimen YPM 1569a is the largest of the syntype series and closely matches Verrill’s description of the species, therefore, we designate this specimen as the lectotype of *Muricea austera* with the purpose of clearly establishing its taxonomic status.

##### Other material revised.

COSTA RICA: UCR 623–624, 633a, dry, Bajo Isla Chora, Sámara Bay, Guanacaste, 10 m, H. Guzman, 18 March 1984; UCR 779, dry, Peñón de la Bruja, Santa Rosa National Park, 10 m, J. Cortés, 5 March 1994; UCR 836, dry, Cabo Blanco Islet, SW from Cabo Blanco National Reserve, Puntarenas, 12 m, L. Mena, 26 April 1994. ECUADOR: IIN 4b, 5, 6, dry, Tambip, Salinas, 12–14 m, F. Rivera, P. Martínez, 20 July 2010; IIN 23, dry, Bajo Lunes, Salinas, 18 m, F. Rivera, P. Martínez, 21 July 2010; IIN 62, dry, Gigima, Salinas, 12–14 m, F. Rivera, P. Martínez, 22 July 2010; IIN 104, 118, dry, Los Ahorcados, Machalilla National Park, 10–12 m, F. Rivera, P. Martínez, 25 July 2010. EL SALVADOR: UCR 1936, ethanol preserved, Departamento la Libertad, Playa Mizata, J. Segovia, 26 February 2010. MÉXICO: M 11, dry, León echado Island, San Carlos Bay, Sonora, 5–25 m, J.L. Carballo, 6 December 2000; M 29, dry, Lobos Island, Mazatlan Bay, Sinaloa, 5–25 m, J.L. Carballo, 17 November 1998. STRI 1128, ethanol preserved, El Faro, Oaxaca, 8 m, R. Abeytia, 2 September 2004; STRI 1139, ethanol preserved, El Faro, Oaxaca, 8 m, R. Abeytia, 13 August 2004. PANAMÁ: STRI 25, 27, dry, Santa Cruz Island, Chiriquí Gulf, 5–10 m, H. Guzman, 10 December 2001; STRI 143, 204, Jicarita Island, Chiriquí Gulf, 10–20, H. Guzman, 9 May 2002; STRI 145, dry, Jicarita Island, Chiriquí Gulf, 10–30 m, H. Guzman, 19 April 2002; STRI 290, Piedra Hacha, Chiriquí Gulf, 20–30m, H. Guzman, 22 April 2002; STRI 319, dry, Chiriquí Gulf, 5–10 m, H. Guzman, 24 April 2002; STRI 406, Seca Grande Islande Chiriquí Gulf, 20 m, H. Guzman, 26 August 2002; STRI 417, Chiriquí Gulf, 20 m, H. Guzman, 26 August 2002; STRI 445, Jacarita Island, 10–25 m, H. Guzman, 29 August 2002; STRI 525, Bajo Bolano, Chiriquí Gulf, 25 m, H. Guzman, 16 April 2003; STRI 751, Roca Trollope, 10–20 m, H. Guzman, 6 August 2003; STRI 777, Pearl Islands, 3 m, H. Guzman, 11 August 2003; STRI 791, San José Island, 3 m, H. Guzman, 11 October 2003; STRI 810, Pearl Islands, 2 m, H. Guzman, 6 April 2004; SRTI 821, 822, Pearl Islands, 3 m, H. Guzman, 6 April 2004; STRI 834, Pearl Islands, 27 m, H. Guzman, 7 April 2004; STRI 838, Pearl Islands, 2 m, H. Guzman, 8 April 2004; STRI 845, Pearl Islands, 3 m, H. Guzman, 20 April 2004; STRI 866, Achotines, Chiriquí Gulf, 3–10 m, H. Guzman, 5 May 2004; STRI 900, 901, 930, Pearl Islands, 10 m, H. Guzman, 23 September 2004; STRI 943, Pearl Islands, 3–20 m, H. Guzman, 23 September 2004; ethanol preserved, STRI 137B,145B, 1213, Saboga Island,1–5 m, H. Guzman, 14 December 2001; STRI C5, Coiba Island, Chiriquí Gulf, H. Guzman, 3 August 2002.

#### Muricea albida

Taxon classificationAnimaliaAlcyonaceaPlexauridae

Verrill, 1868

Figures 27 , 28

Muricea robusta (pars.) Verrill, 1866: 329.
Muricea albida Verrill, 1868b: 412; Verrill 1869: 437–439; Kükenthal 1919: 752; Kükenthal 1924: 146; Hickson 1928: 363–364; Riess 1929: 393–394; Harden 1979: 142–143.

##### Material.

Lectotype. YPM 1559a, dry, Pearl Islands, Panamá, 11–14 m, F.H. Bradley, 1866. Paralectotypes. PANAMÁ: MCZ 712; MCZ 4976; MCZ 7016; dry; YPM 563, 3 specimens; YPM 563h; YPM 1559b-d, ZMUC-ANT 190, 2 specimens (part of YPM 1559); dry; YPM 1633, 2 fragments, ethanol preserved, data as in the lectotype.

##### Description.

**Figure 27.**
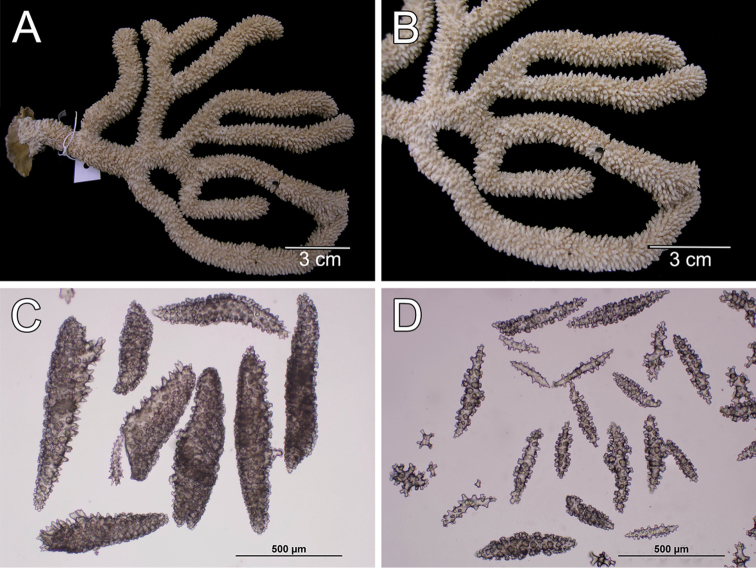
*Muricea albida* Verrill, 1868b. YPM 1559a. **A** Colony **B** Detail of branches **C–D** Sclerites, light micrographs.

**Figure 28.**
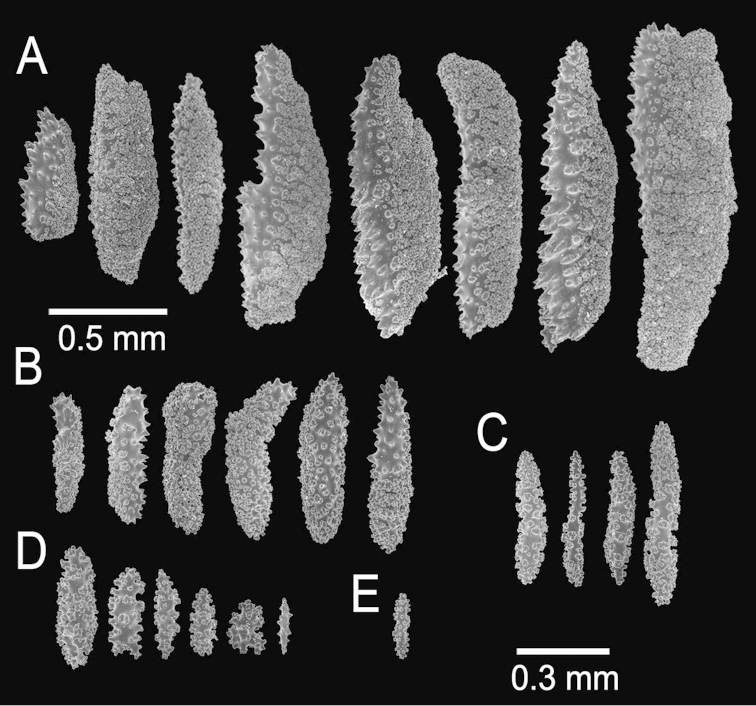
*Muricea albida* Verrill, 1868b. YPM 1559a. **A–C**. Calycular and coenenchymal sclerites **D** Axial sheath sclerites. **E** Anthocodial sclerites.

The lectotype is a 15 cm long and 12 cm wide, candelabrum-like colony, branching in one plane and mostly dichotomous (Fig. 27A). The colony arises from a conical holdfast, about 24 mm in diameter, partially devoid of coenenchyme. The stem is 10 mm in diameter slightly flattened, and 23 mm long, it bifurcates producing secondary branches that subdivide again at distances up to 1.5 cm apart, two of them remain as long simple branchlets. The branches and branchlets are stout, 7–10 mm in diameter and are tapered toward the tips, reaching 5–7 mm in diameter. The branches split at close angles, about 45°, and some branchlets project at acute or wider angles. The unbranched terminal ends are up to 11 mm long. The calyces are all around the branches, close together, slightly imbricate at the upper part of the branches (Fig. 27B). They are elongated, sub-conical, about 2 mm long, and mostly project at right angles to the branches. The coenenchyme is thick and compact. The sclerites are white, and greyish (Fig. 27C–D). The outer coenenchyme and calycular sclerites are of various types. The larger ones are unilateral spinous spindles with the inner side with small numerous warts and the outer side with short spines, they are elongated, with blunt ends, or with one end tapered and the other wide and blunt, 0.60–1.41 mm long and 0.25–0.05 mm wide, (Fig. 28A). Furthermore, spiny spindles with one spiny end and a warty body are present, 0.29–0.6 mm long and 0.09–0.14 mm wide (Fig. 28B), the sharp or spiny ends project beyond the calyx border; and warty, elongated spindles 0.44–0.60 mm long and 0.07–0.11 mm wide (Fig. 28C). The axial sheath is composed of warty, irregular spindles, and radiates 0.18–0.30 mm long and 0.05–0.14 mm wide with acute or bifurcated ends, and radiates (Fig. 28D). Anthocodial sclerites are flat warty rods, some with one bifurcated end, 0.11–0.25 mm long and 0.01–0.03 mm wide (Fig. 28E).

Colour of the colony is white.

##### Distribution.

It is known from the type locality, Pearl Islands, and reported from Taboguilla Island. However, *Muricea albida* has not been found during our recent extensive surveys along Pacific Panamá.

##### Remarks.

The species was described by Verrill (1869) with specimens from Panamá, Hickson (1928) added some not relevant details to the description. Verrill did not designate a holotype for the species, thus we chose specimen YPM 1559a, that is probably the figured specimen (Verrill 1869 Pl. 7, Fig. 9), as the lectotype of *Muricea albida* to clearly establish its taxonomic status.

##### Other material revised.

PANAMÁ: BM 1946.1.14.43, off Taboguilla, 9 m, C. Crossland, 22 November 1915.

#### Muricea crassa

Taxon classificationAnimaliaAlcyonaceaPlexauridae

Verrill, 1869

Figures 29 , 30

Muricea crassa Verrill, 1869: 425–426; Kükenthal 1919: 752; Kükenthal 1924: 144; Riess 1929: 392–393.

##### Material.

Lectotype: YPM 1798 (figured specimen), dry, Pearl Islands, Panamá, F.H. Bradley, 1866, no further data. Paralectotypes: PANAMÁ: MCZ 702; MCZ 7015; YPM 1558; ZMUC-ANT 19, same data as the lectotype.

##### Description.

**Figure 29.**
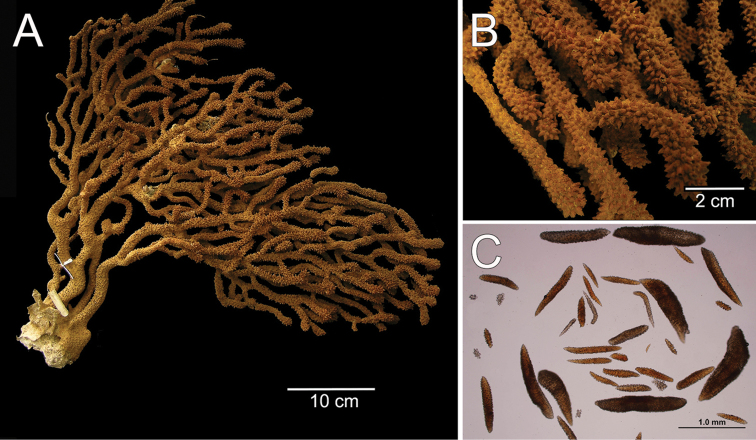
*Muricea crassa* Verrill, 1869, YPM 1798. **A** Colony **B** Detail of branches **C** Sclerites, light micrograph.

**Figure 30.**
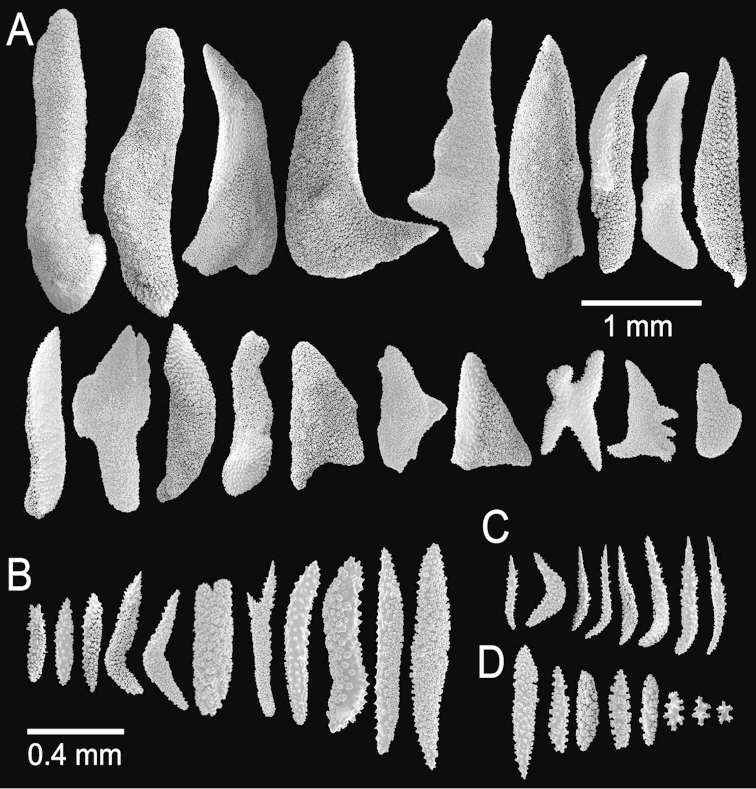
*Muricea crassa* Verrill, 1869. YPM 1798. **A–B** Calycular and coenenchymal sclerites **C** Anthocodial sclerites **D** Axial sheath sclerites.

The lectotype is a large bushy colony, 40 cm long and 50 cm wide. The holdfast is irregular, and swollen, about 7 cm wide and 4 cm high covering one side of the rock remains to which is attached. The branching is irregularly dichotomous, mostly lateral, in one plane (Fig. 29A). There is not a common stem, but 4 main branches arise directly from the holdfast. They are up to 15 mm in diameter, extending up to 7.5 cm and subdivide in secondary branches, that bifurcate up to 6 times producing subordinate branches, no more than 25 mm apart. They project at angles 45°–90°, and curve upwards. They are of the same diameter, and thicker toward the tips, which are wide and clavate. Some branch anastomoses occur, especially at the base. Unbranched terminal ends are up to 7 cm long, with expanded tips 7–10 mm in diameter, some short branchlets 1–2 cm long, are up to 12 mm in diameter. Axes are of a dark brown at the base, lighter at the tips. The calyces are all around the branches, close together, not imbricate (Fig. 29B). They are prominent, up to 3 mm long, covered with large spindles with echinulate ends. The calyces on the lower branches are more conical and blunter that the ones on the upper branches, also more distantly spaced and smaller. The coenenchyme is coarse, composed of large and irregular spindles, they are reddish-brown and of lighter and darker hues (Fig. 29C). Outer coenenchymal and calycular sclerites are large and strong. They are mostly unilateral spinous spindles with an inner surface of crowded complex small warts and an outer surface with short sparsely placed spines or prickles. They are of diverse shapes, unequal with one side truncate and the other acute, bifurcated at one side, others are triangular or with almost rectangular forms, 0.56–2.5 mm long and 0.40–0.70 mm wide (Fig. 30A). The calycular wall is composed of elongated spindles with acute ends and spindles with prickly terminal spikes or bifurcated, 0.92–0.32 mm long and 0.8–0.1 mm wide (Fig. 30B). These types of sclerites give the calyces a stout, rough appearance characteristic of this species. The axial sheath is composed of small, colourless radiates, and small spindles, 0.08–0.38 mm long and 0.07–0.09 mm wide (Fig. 30D). Anthocodial sclerites are light orange to yellowish spindles. They have long and sharp terminal spines or with spiny shafts, 0.3–0.65 mm long and 0.04–0.12 mm wide (Fig. 30C) that are at the base of the polyps. Furthermore, some irregular branched forms, 0.15–0.2 mm long and 0.03–0.06 mm wide, and small rods 0.09–0.3 mm long and 0.01–0.04 mm wide.

Colour of the colony is dark brown.

##### Habitat and variability.

The examined colonies are up to 50 cm tall and up to 40 cm wide, but smaller specimens, around 30 cm tall are the majority. The branch tips could reach up to 15 mm in diameter, and the unbranched terminal ends up to 13 cm long. This species is very conspicuous because of the dark colour and strong and prickly branches with wide terminal tips. We have observed *Muricea crassa* at various localities in Panamá, from 3–12 m deep around Pearl Islands, Gulf of Panamá and the deepest records are around Coiba Island in the Gulf of Chiriquí, 20–30 m deep. It is not abundant and is sparsely distributed in patches dominated by other species.

##### Distribution.

It has a widespread distribution from México to Perú. Type locality, Pearl Islands, Panamá.

##### Remarks.

Verrill (1868b) described this species with specimens from Pearl Islands, Panamá; he registered larger sizes of sclerites, up to 3.2 mm long and 0.875 mm wide, however the other characteristics are very consistent with the examined specimens. Verrill did not designate a holotype for the species. We chose the specimen YPM 1798 as the lectotype of the species with the purpose of clearly establishing the taxonomic status of *Muricea crassa*. On the label of specimen YPM 1798 it was written down this as Verrill’s (1868b) figured specimen.

##### Other material revised.

ECUADOR: IIN 94, 121, dry, Los Ahorcados, Machalilla National Park, 10–12 m, F. Rivera, P. Martínez, 25 July 2010. PANAMÁ: STRI 28, dry, Santa Cruz Island, Chiriquí Gulf, 5–10 m, H. Guzman, 10 December 2001; STRI 104, dry, Isla Barca, 5–10 m, H. Guzman, 18 April 2002; STRI 292, dry, Piedra Hacha, 5–10 m, H. Guzman, 22 April 2002; STRI 354, dry, Almohada Island, 5–15 m, H. Guzman, 29 April 2002; STRI 374, 375, 376, dry, Toboguilla Island, 5–10 m, H. Guzman, 9 May 2002; STRI 553, etanol preserved, Bolanito Island, 6 m, H. Guzman, 16 April 2003; STRI 768, San Telmo Island, 3–8 m, H. Guzman, 7 August 2003; STRI 779, 781, 782, Pedro Gonzales Island, 3 m, H. Guzman, 11 August 2003; STRI 787, San Jose Island, 4 m, H. Guzman, 10 October 2003; STRI 907, 908, Pedro Gonzales Island, 10 m, H. Guzman, 23 September 2004.

#### Muricea retusa

Taxon classificationAnimaliaAlcyonaceaPlexauridae

Verrill, 1869

Figures 31 , 32

Muricea retusa Verrill, 1869: 432–434; Kükenthal 1919: 752; Kükenthal 1924: 148; Harden 1979: 158.

##### Material.

Holotype. YPM 3068, dry, attached to a colony of *Muricea fruticosa*, Pearl Islands, Panamá, 11–14 m, F.H. Bradley, 1866–1867. Schizotype. USNM 1013283, same data as in the holotype.

##### Description.

**Figure 31.**
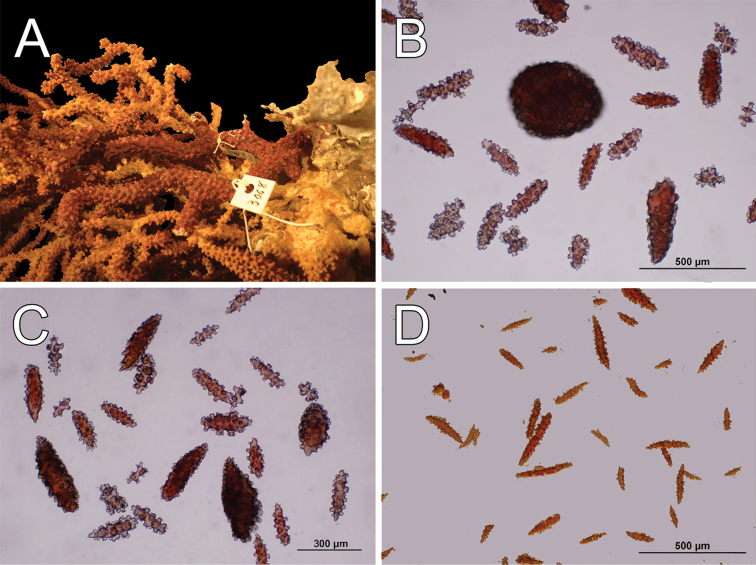
*Muricea retusa* Verrill, 1869, YPM 3068. **A** Colony **B–D** Sclerites, light micrographs.

**Figure 32.**
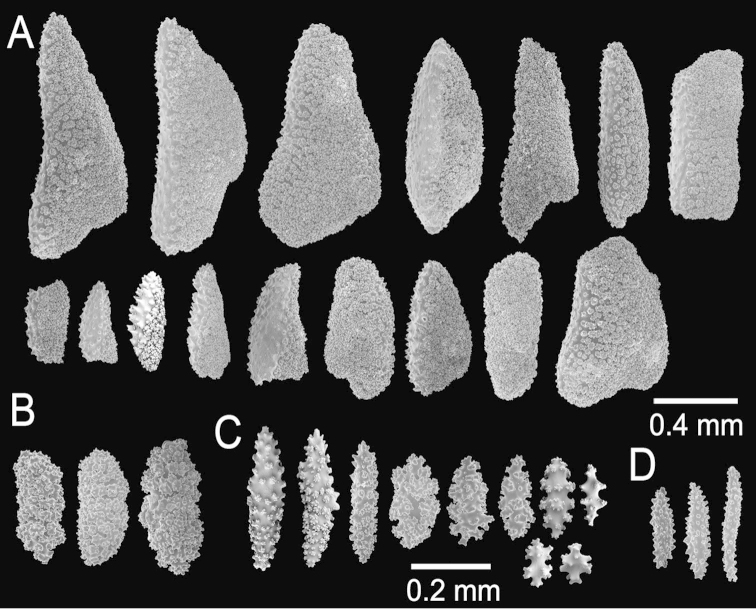
*Muricea retusa* Verrill, 1869, YPM 3068. **A–B** Calycular and coenenchymal sclerites **C** Axial sheath sclerites **D** Anthocodial sclerites.

The holotype is a 7 cm long and about 6 cm wide colony. It is attached to the holdfast of a large *Muricea fruticosa* colony (Fig. 31A). The holotype has a conical holdfast about 14 mm diameter. The branching is dichotomous, in one plane (Fig. 31A). Two main branches arise from a short stem, and bifurcate producing secondary branches, that subdivide again at a distance 25–50 mm apart. The branches split at angles of about 45°, and the only one that is complete is slightly curved, and clavate at the end. The branches are 7–8 mm in diameter and are of the same diameter along their length, and little enlarged toward the tips. Unbranched terminal ends are up to 50 mm long, but most are stumps. The calyces are all around the branches, close together, not imbricated but little overlapped and composed of two horizontal rows of sclerites. They are sub-conical, as wide as long, 1–1.5 mm in height, directed upwards at angles of 30°–45° at the upper branches and at greater angles at the lower branches, also more distantly spaced and smaller (Fig. 31A). The coenenchyme is coarse, composed of large and irregular-shaped spindles giving a granulose aspect to the colony. The sclerites are reddish purple, purple varying to light orange (Fig. 31B–D). The outer coenenchymal and calycular sclerites are mostly unilateral spinous spindles, 0.35–1.2 mm long and 0.15–0.6 mm wide, one side crowded by small warts and the other spinulate, with short or large spines. They are of diverse shapes, unequal with one side truncate and the other acute, with triangular or with almost rectangular forms (Fig. 32A), and warty cylindrical spindles 0.30–0.45 mm long and 0.12–0.20 mm wide (Fig. 32B). The axial sheath is composed of light orange irregular spindles and radiates, 0.10–0.30 mm long and 0.06–0.14 mm wide (Fig. 32C). Anthocodial sclerites are orange and light yellow irregular spindles and warty rods 0.06–0.40 mm long and 0.02–0.10 mm wide (Figs 31D, 32D).

Colour of the colony is reddish purple.

##### Distribution.

It is only known from the type locality, Pearl Islands, Panamá.

##### Remarks.

Verrill (1869) described this species from just one specimen on the holdfast of a 36 cm by 29 cm *Muricea fruticosa* colony. Except for Harden (1979), who mentioned this species, no other author has referred to it, and no other specimens are identified as *Muricea retusa* in the museums visited.

#### Muricea purpurea

Taxon classificationAnimaliaAlcyonaceaPlexauridae

Verrill, 1864

**Figure 33.**
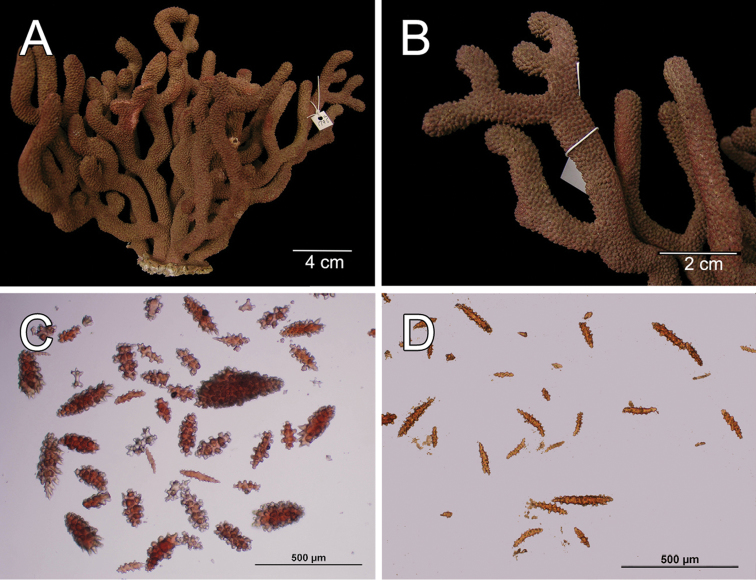
*Muricea purpurea* Verrill, 1864, YPM 1795a. **A** Colony **B** Detail of branches **C–D** Sclerites, light micrographs.

**Figure 34.**
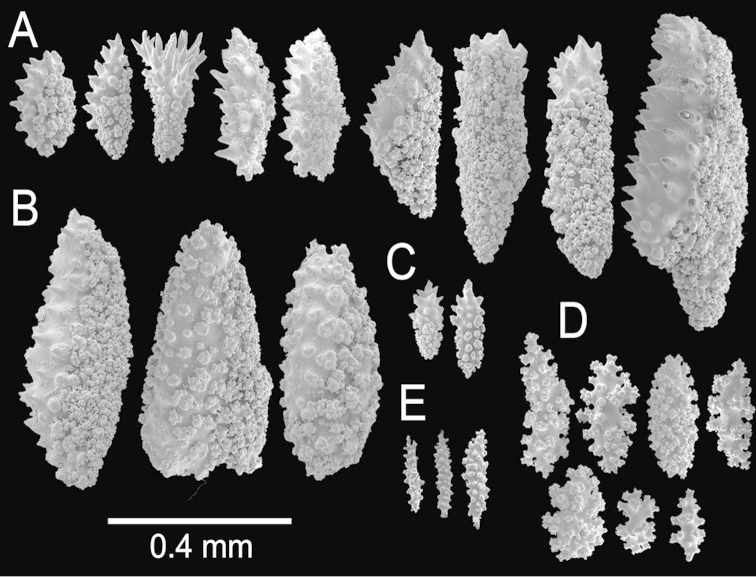
*Muricea purpurea* Verrill, 1864, YPM 1795a. **A–C** Calycular and coenenchymal sclerites **D** Axial sheath sclerites **E** Anthocodial sclerites.

**Figure 35.**
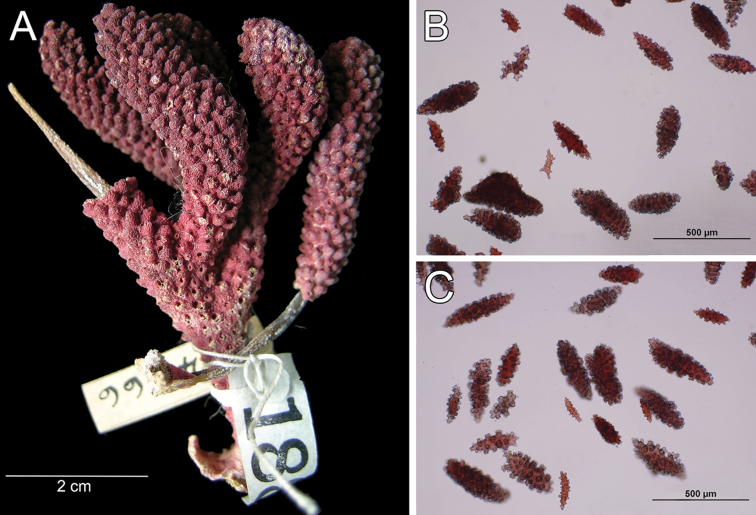
*Muricea purpurea* Verrill, 1864, MCZ 4066. **A** Colony **B–C** Sclerites, light micrographs.

**Figure 36.**
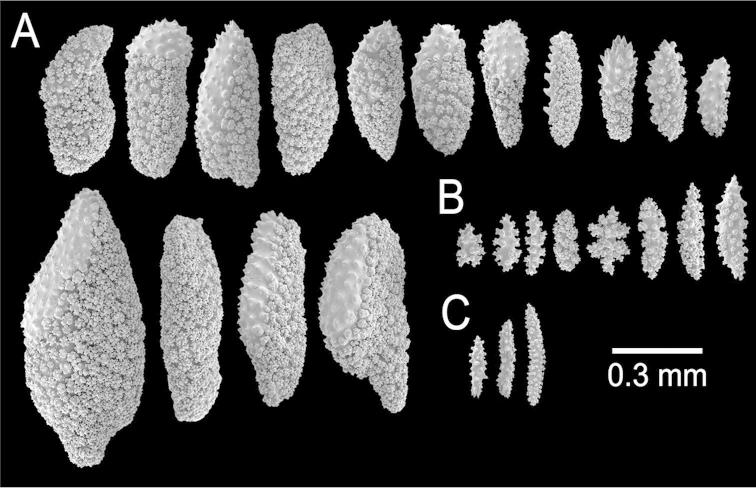
*Muricea purpurea* Verrill, 1864, MCZ 4066. **A** Calycular and coenenchymal sclerites **B** Axial sheath sclerites **C** Anthocodial sclerites.

**Figure 37.**
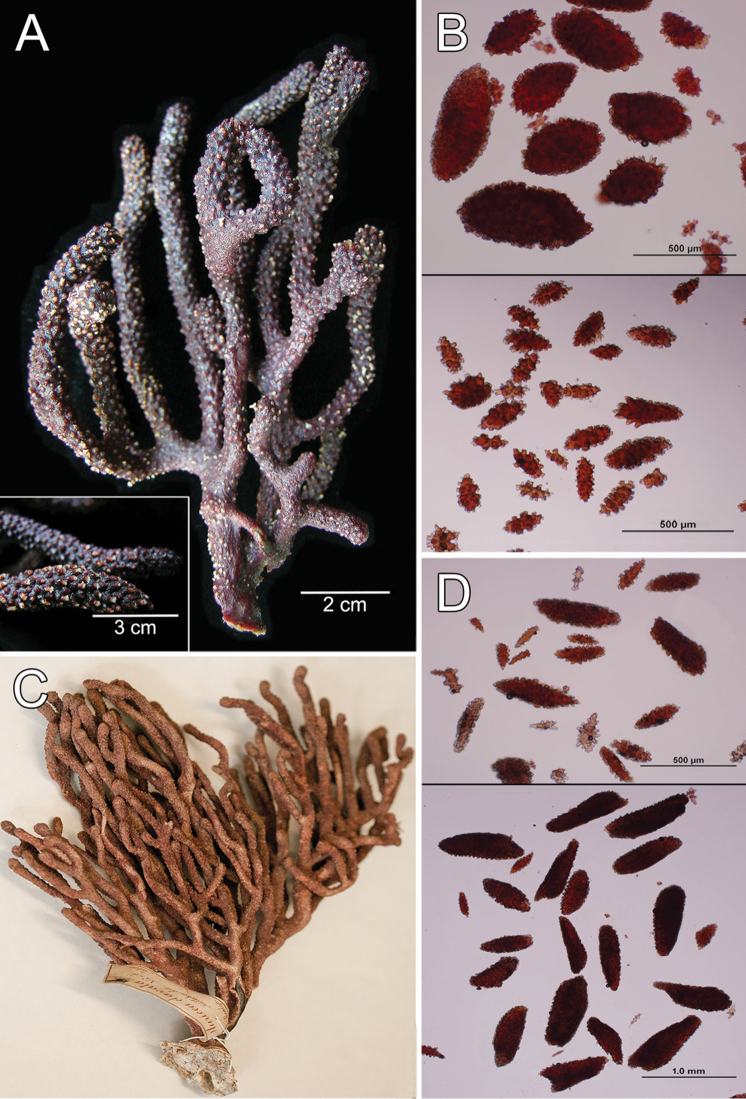
*Muricea purpurea* Verrill, 1864. **A** ZMUC ANT-142 (Muricea purpurea var. nigra Hickson, 1928, syn. n.) colony **B** Sclerites, light micrographs **C** SMNH 1693 (*Muricea rubra* Aurivillius, 1931, syn. n.) colony, photograph: Elin Sigvaldadottir **D** Sclerites, light micrographs.

**Figure 38.**
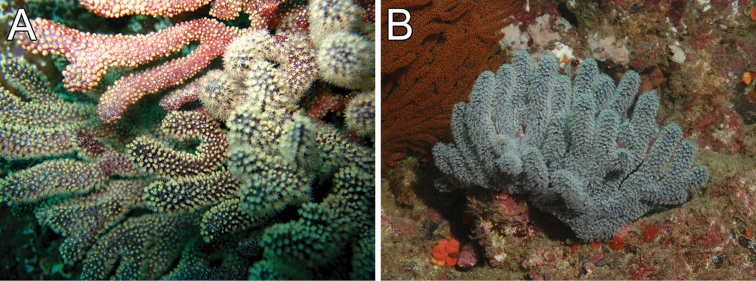
*Muricea purpurea* Verrill, 1864, colonies *in situ*, submarine pictures. **A** Oxaca, México, photograph: Rosalinda Abeytia **B** Ahorcados Islet, Machalilla National Park, photograph: Graham Edgar.

Figures 33 , 34 , 35 , 36 , 37 , 38

Muricea hebes (pars.) Verrill, 1864: 36.
Muricea purpurea Verrill 1868b: 412; Verrill 1869: 441–444; Kükenthal 1919: 752; Kükenthal 1924: 146; Hickson 1928: 366–367; Riess 1929: 394–395; Stiasny 1943: 66–68; Harden 1979: 157–158.
Muricea purpurea var. nigra Hickson, 1928: 367 syn. n.
Muricea rubra Aurivillius, 1931: 108–109 syn. n.

##### Material.

Lectotype. YPM 1795A, dry, Pearl Islands, Panamá, F.H. Bradley, 1866–1867, no more data. Paralectotypes. PANAMÁ: MCZ 7018 (707, fragment); YPM 808; YPM 1560A-G; YPM 1795B; ZMUC ANT-194 (YPM 1560), dry, Pearl Islands, F.H. Bradley, 1866–1867, no more data. YPM 1637 (fragment), alcohol preserved; Pearl Islands, F.H. Bradley, 1866–1867, no more. MÉXICO: MCZ 55, ethanol preserved; Acapulco, A. Agassiz, 1859–1860, no more data. MCZ 4066 (188); MCZ 4067 (188); YPM 391, dry, Acapulco, A. Agassiz, 1859–1860, no more data.

##### Other type material.

Panamá: BM 1946.1.14.44, dry, off Taboguilla Island, 9 m, T. Mortensen, 27 November 1915. USNM 34062, dry, Gulf of Panamá, Panamá, no more data. ZMUC ANT-142 (Hickson’s holotype of Muricea purpurea var. nigra, ethanol preserved, Taboga Island, Panamá, 9 m, T. Mortensen, 25 November 1915. NICARAGUA: SMNH 1693 (Aurivillius’s holotype of *Muricea rubra*); USNM 44190 (fragment of SMNH 1693), dry, off Realejo, Leg. Palme, no more data.

##### Description.

The lectotype is a 22 cm long and 21 cm wide colony with branching in one plane and mostly dichotomous (Fig. 33A). The colony is composed of four stems that arise from a spreading holdfast about 5 mm in diameter and devoid of coenenchyme at the base. The four stems, 6–11 mm in diameter, are slightly flattened, and 12–45 mm long. They bifurcate producing secondary branches, that subdivide again at distances of 12–75 mm apart, 2–3 branches do not subdivide, reaching up to 12 cm long, but most of them do, some of them up to 5 times. The branches are stout, 12–14 mm in diameter and are little tapered toward the tips, 9–11 mm in diameter. The branches are wider and flattened at the branching points, 12–14 mm in diameter. They are mostly crooked, split at close angles 45°–60° at the upper branches, and at wider angles close to the base; these branches curve and some of them bend upwards (Fig. 33A). The unbranched terminal ends are 50–80 mm long. Axes are brownish and with darker hues at the thicker branches. The calyces are all around the branches, close together and slightly imbricate (Fig. 33B). They are small, up to 1.8 mm long, sub-conical, with a granulose appearance. The calyces extend upwards with the tips pointed and often incurved; they are smaller and truncated at the lower parts of the branches. The coenenchyme is thick, sclerites are dark red and reddish orange (Fig. 33C–D), and those from the axial sheath are pink. The calycular and the outer coenenchymal sclerites are leaf-like spindles, 0.3–0.70 mm long and 0.10–0.30 mm wide, with spiny lateral process and a warty surface (Fig. 34A). Spinous club-like spindles are abundant especially toward the calyces and slightly project beyond the calyx border. They are stout and rough, 0.15–0.20 mm long and 0.07–0.08 mm wide, with a warty base, and a thorny head (Fig. 34C). Unilateral spinous spindles are smaller, 0.23–0.62 mm long and 0.13–0.30 mm wide, with one side warty and the other spinulose (Fig. 34B). The axial sheath is composed of irregular spindles up to 0.24–0.40 mm long and 0.10–0.14 mm wide with acute or bifurcated ends and tuberculate radiates 0.13–0.21 mm long and 0.09–0.14 mm wide (Fig. 34D). Anthocodial sclerites are reddish orange, composed of warty rods, 0.09–0.30 mm long and 0.03–0.055 mm wide, (Fig. 34E).

Colour of the colony is reddish purple.

##### Habitat and variability.

Verrill’s type collection is composed of specimens from Panamá and Acapulco, México. The description of *Muricea purpurea* was mostly based on the Panamá specimens (Verrill 1869), YPM 1795 was the figured specimen (Verrill 1869 plate VII, 6). However we found that there are two different morphologies among the specimens. The ones from Mexico are finger-like colonies, composed of one or more single branches, with a more intense red colour (reddish purple) and with larger calyces (Fig. 35A) than the specimens from Panamá. The largest sclerites in the Mexican specimens reach up to 1.0 mm long (Fig. 36A), larger than 0.625 mm as stated by Verrill for *Muricea purpurea*. The larger sclerite sizes were not found in the typical series from Panamá, where the maximum size was 0.70 mm. The sizes and types of the rest of sclerites are mostly consistent with the typical specimens (Fig. 36B–C). The sclerites of the specimens from México in the YPM type series are morphologically consistent among them, but in the Panamá specimens we found both sclerite morphologies (e.g., YPM 7018 from Panamá matches the sclerites of the Mexican morphotype). There is a series of intermediate types of sclerites among the examined specimens and the lectotypes (Figs 35B–C; 37B, D). In some specimens there is a dominance of spindles with terminal spiny processes, others with lateral spiny processes. In some cases the spines of the leaf-like spindles are shorter than in others, e.g., paralectotype MCZ 4066 (Fig. 35A–C) and MCZ 4067. The sclerite colours are mostly as in the lectotype, but some variation toward darker hues was observed. The colour of the colonies is from reddish purple to dark purple (Figs 35A; 37A, C; 38A–B). The extremes can be observed in the former *Muricea rubra* and Muricea purpurea var. nigra (syns. n. in this paper). The lighter colours are in the former and the darker hues in the latter (Fig. 37A, C). We have found all these morphologies in our recent collections from Costa Rica, Panamá, Ecuador, Nicaragua and México. Perhaps population biology research of these communities could reveal affinities among the morphotypes that could justify further species separation.

The colonies are found on rocky substrates, mostly vertically placed or upside down in caves. They also occur in crevices at rocky bottoms and grow straight up. The colonies can extend along the substrate by spreading holdfast up to 30 cm long producing separate branches forming large colonies. When alive, polyps look, or greenish yellow (Fig. 38A), or whitish on a dark purple colony (Fig. 38B). When polyps retract colonies look darker, more blackish (Fig. 38A).

##### Distribution.

It is a widespread species distributed from México to Perú. The species has been reported for Acapulco, México; Corinto, Nicaragua; Ecuador (Kükenthal 1924), from Santa Clara Island to Esmeraldas (Rivera and Martínez 2011) and Panamá. We have observed it at several sites along the Pacific coast from México to Perú. The depth range is from 3 to 25 m, but mostly it occurs at 8 to 15 m.

Type locality, Pearl Islands, Panamá.

##### Remarks.

The species was first mentioned by Verrill (1864) together with other species that he separated and properly described later (1869). *Muricea purpurea* was described with specimens from Panamá and México, Verrill did not designate a holotype. The specimen YPM 1795A is herein designated as the lectotype of this species in order to clearly establish its taxonomic status.

There are two other related species *Muricea rubra* and Muricea purpurea var. nigra. Hickson (1928) proposed a variety of *Muricea purpurea* (var. *nigra*) based on the colour (very dark purple) and the ramification (bushier). However, according to Hickson the sclerites of this species were that close to *Muricea purpurea* that he could not consider them as separate species. We conclude that Hickson´s ZMUC ANT-142 specimen is in the variation range of *Muricea purpurea*, for this reason it is considered herein as a synonym. *Muricea rubra* was described by Aurivillius (1931) with a specimen from Nicaragua. The author stated that he never had the opportunity to revise any material previously established and that he could not identify the species from the existing descriptions at that time (Aurivillius 1931, pag.104). We analysed Aurivillius’ holotype (SMNH 1693) and, as in the case of Muricea purpurea var. nigra, we did not find *Muricea rubra* out of the variation range of *Muricea purpurea*. Herein it is also considered as a synonym of *Muricea purpurea*.

##### Other material revised.

COSTA RICA. UCR 479a, dry, Herradura Beach, 10 m, J Cortés, 2 September 1983; UCR 510, 632a, dry, Sámara Beach, Guanacaste, 12 m, H. Guzman, 18 March 1984; UCR 800, dry, Olocuita Islet, Manuel Antonio National Park, Puntarenas, 8 m, J Cortés, 2 July 1995; UCR 1620, ethanol preserved, Carrillo Beach, Guanacaste, 10 m, J Cortés, 2006; UCR 1693, ethanol preserved, Salinas Bay, Guanacaste, O Breedy, 7 December 2006. ECUADOR: IIN 20, dry, Tambip, Salinas, 12–14 m, F. Rivera, P. Martínez, 20 July 2010; IIN 43, 48, dry, Gigima, Salinas, 12–14 m, F. Rivera, P. Martínez, 22 July 2010; IIN 99, 117, 119, 120, dry, Los Ahorcados, Machalilla National Park, 10–12 m, F. Rivera, P. Martínez, 25 July 2010; IIN 129, dry, Salango Island, Machalilla National Park, 12–25 m, F. Rivera, P. Martínez, 25 July 2010. PANAMÁ: STRI 360, dry, Otoque Island, Chiriquí Gulf, 5–10 m, H. Guzman, 9 May 2002; STRI 361, dry, Otoque Island, 5–10 m, H. Guzman, 9 May 2002; STRI 378, dry, Taboguilla Island Chiriquí Gulf, 5–10 m, H. Guzman, 9 May 2002; STRI 716, H Station, 45 m, H. Guzman, 6 August 2003; STRI 766, 767, San Telmo Island, 3–8 m, H. Guzman, 7 August 2003; STRI 784, San Telmo Island, 3 m, H. Guzman, 10 October 2003; STRI 809, 813, Del Rey SE Island, 4 m, 6 April 2004 ; STRI 823, Puerco Island, 3 m, H. Guzman, 6 April 2004; STRI 827, Elefante Island, 4 m, H. Guzman, 7 April 2004 ; STRI 847, Sur Pacheca, 2 m, H. Guzman, 20 April 2004 ; STRI 854, Pearl Island, 3 m, H. Guzman, 21 April 2004 ; STRI 855, Pearl Island, 4 m, H. Guzman, 21 April 2004 ; STRI 860, 861, Pearl Island, 2–4 m, H. Guzman, 23 April 2004 ; STRI 894, San Telmo Island, 3–8 m, H. Guzman, 18 August 2004 ; STRI 905, Pedro Gonzales Island, 10 m, H. Guzman, 23 September 2004; STRI 931, 932, 933, Pedro Gonzales Island, 10 m, H. Guzman, 23 September 2004.

#### Muricea hebes

Taxon classificationAnimaliaAlcyonaceaPlexauridae

Verrill, 1864

Figures 39 , 40

Muricea hebes (pars.) Verrill, 1864: 36.
Muricea hebes Verrill 1866: 328; Verrill 1868b: 411–412; Verrill 1869: 439–441; Kükenthal 1919: 752; Kükenthal 1924: 146; Hickson 1928: 365–366; Riess 1929: 395–396; Harden 1979: 151.

##### Material.

Lectotype. YPM 564a, dry, Pearl Islands, Panamá, F.H. Bradley, 1866. Paralectotypes. YPM 564 b-f, dry, Pearl Islands, Panamá, F.H. Bradley, 1866.

##### Description.

**Figure 39.**
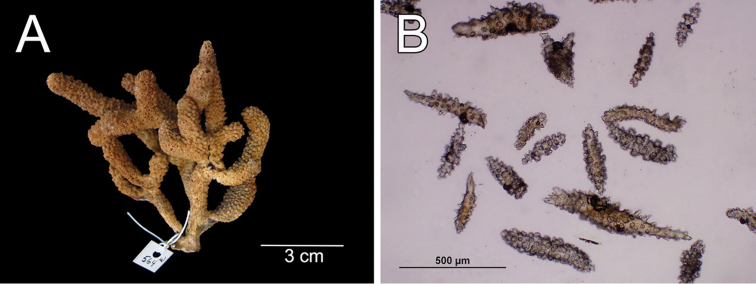
*Muricea hebes* Verrill, 1864. YPM 564a. **A** Colony **B** Sclerites, light micrographs.

**Figure 40.**
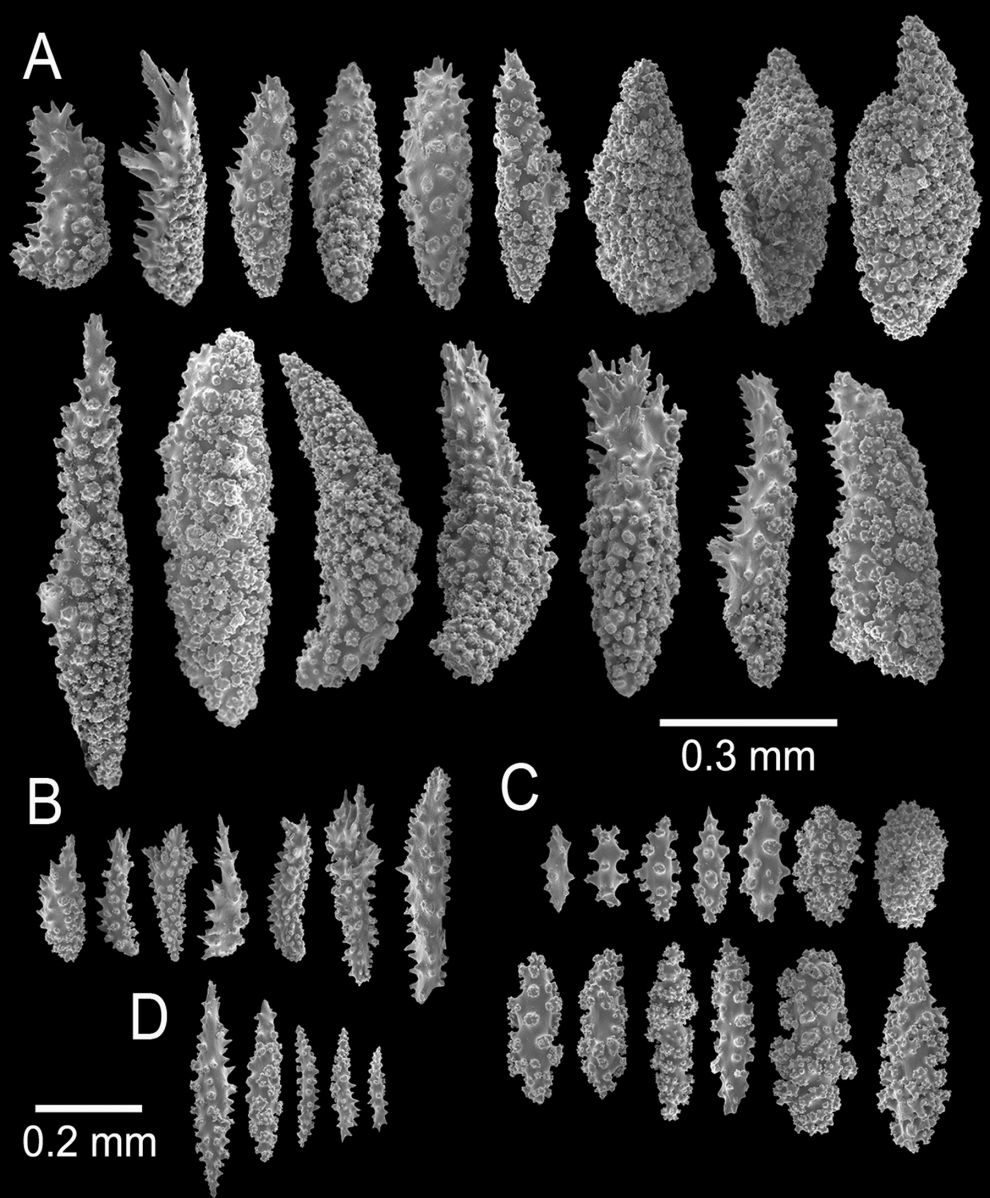
*Muricea hebes* Verrill, 1864. YPM 564a. **A–B** Calycular and coenenchymal sclerites **C** Axial sheath sclerites **D** Anthocodial sclerites.

The lectotype is 6 cm long and 9 cm wide (Fig. 39A); it has a worm tube in one of the lower lateral branches. Two slightly flattened stems, 5–6 mm in diameter, arise from an oval holdfast, about 1 cm diameter, almost devoid of coenenchyme (Fig. 39A). The branching is irregularly dichotomous and multiplanar (Fig. 39A). The stems extend 12–15 mm and subdivide in secondary branches that bifurcate up to 4 times at close angles 30°–40°, and curve upwards. Branches are 10–12 mm apart. They are flattened, 5–8 mm in diameter, little wider at the tips, 6–9 mm in diameter. Unbranched terminal ends are up to 3.2 cm long. Axes are brownish. Calyces are all around the branches, curved upwards and close together, slightly imbricate (Fig. 39A), up to 1.8 mm long, and around 1 mm wide. Polyps are on the upper and inner part of the calyces, nearly covered by the incurved outer border of the calyces. The adaxial border of the calyx is imperceptible. The coenenchyme is thick, composed of pale yellow and yellowish sclerites (Fig. 39B). The outer coenenchymal and calycular spindles are of various types (Fig. 40A–B), unilateral spinous with a warty inner side and a small outer portion with projecting spines, and with the inner side warty, and the outer side with large sharp spines, prickly spindles and spinous club-like spindles. These sclerites are 0.32–0.83 mm long and 0.14–0.2 mm wide. They vary from elongated to shorter forms, with round ends, or with one end tapered and the other wide and blunt, or with one end acute or bifurcated, or tapered at both ends (Fig. 40A). Furthermore, smaller forms are present, 0.24–0.28 mm long and 0.07–0.1 mm wide (Fig. 40B), that concentrate around the calyx border (Fig. 40B). The axial sheath is composed of warty radiates and spindles, 0.15–0.40 mm long and 0.053–0.15 mm wide (Fig. 40C). Anthocodial sclerites are warty rods, 0.054–0.45 mm long and 0.015–0.1 mm wide, some with distal spines (Fig. 40D).

Colour of the colony is yellowish brown.

##### Distribution.

Found in México in Pájaros Island and reported for Cabo Pulmo, Gulf of California (according to Covarrubias et al. 1996). Type locality, Pearl Islands, Panamá.

##### Remarks.

The species was created by Verrill in 1864 with specimens from Acapulco, México collected by Agassiz. Later Verrill (1869) made a more detailed description and included specimens from Nicaragua and Panamá. However, the type series in the YPM only includes specimens from Panamá collected by Bradley. The specimens from México were included in *Muricea purpurea*. However *Muricea hebes* was found in Mexican islands by J.L. Carballo. The specimen YPM 564a is herein designated as the lectotype of *Muricea hebes* to clearly establish its taxonomic status.

##### Other material revised.

MÉXICO: M 61, dry, Pájaros Island, Mazatlan Bay, Sinaloa, 5–25 m, J.L., 3 February 1999.

#### Muricea nariformis

Taxon classificationAnimaliaAlcyonaceaPlexauridae

Aurivillius, 1931

Figures 41 , 42

Muricea nariformis Aurivillius, 1931: 109–111.

##### Material.

Holotype. SMNH 1121, alcohol preserved, locality unknown, Leg. Salmin 1873, Rijksmuseum, No. 64.

##### Description.

**Figure 41.**
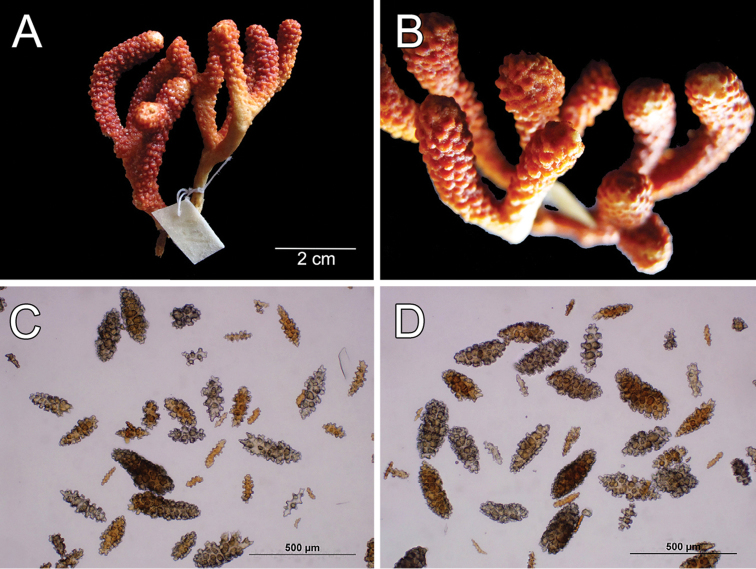
*Muricea nariformis* Aurivillius, 1931. SMNH 1121. **A** Colony **B** Detail of branches **C–D** Sclerites, light micrographs.

**Figure 42.**
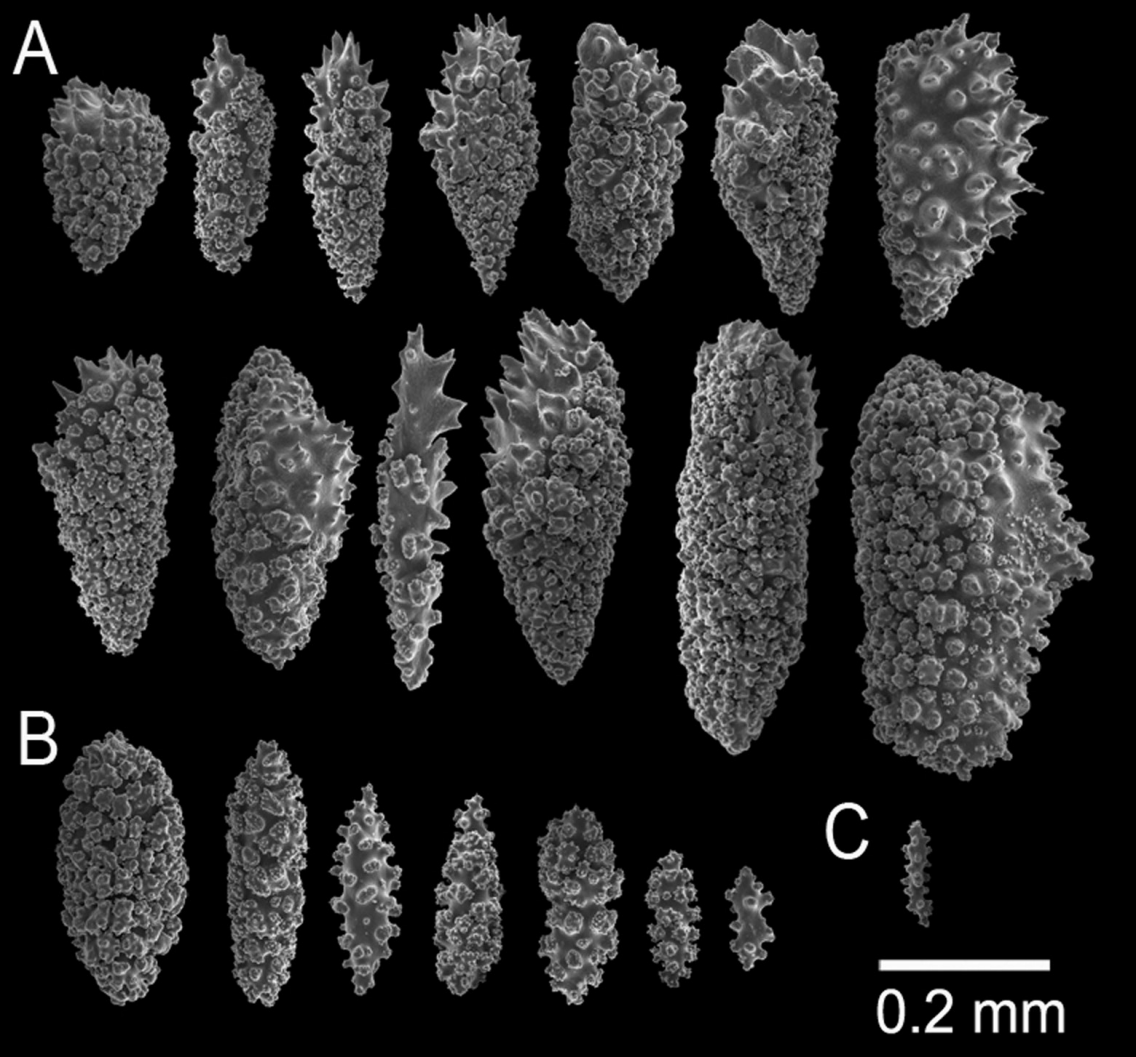
*Muricea nariformis* Aurivillius, 1931. SMNH 1121. **A** Calycular and coenenchymal sclerites **B** Axial sheath sclerites **C** Anthocodial sclerite.

The holotype consists of two branches, about 6 cm long and 3 cm wide each (Fig. 41A). The branching is dichotomous and bifurcate up to 3 times. The branches bifurcate at angles 45°–50°, 7.5–10 mm apart. Branches and branchlets are all of similar thickness, 56 mm in diameter, with rounded tips slightly expanded. Unbranched terminal ends are up to 24 mm long. Axes are brownish. The calyces are all around the branches and close together. They are mostly low cones with a slightly elevated margin around the polyps, 0.8–1.2 mm in height (Fig. 41B), and the abaxial border slightly more prominent and curved upwards (Fig. 41B). The coenenchyme is thick, composed of brownish orange and light brown to whitish sclerites (Fig. 41C–D). As in many other species in this genus, a division of sclerite layers is not clear and the coenenchyme is formed of a combination of several types of sclerites intermingled. The coenenchymal and calycular sclerites are mostly of the same type. Few unilateral spinous spindles are present; the larger ones are rather leaf-like spindles with a thorny lateral quill and wide cylindrical forms (Fig. 42A). The larger ones are covered with warts almost up to the thorny end, and the others have a pronounced upper or lateral ridge of large spines. The spindles are 0.2–0.52 mm long and 0.04–0.28 mm wide (Fig. 42A). Warty spindles and some cylinder-like, 0.25–0.32 mm long and 0.06–0.15 mm wide (Fig. 42B), compose the axial sheath and the inner coenenchyme. Anthocodial sclerites are orange warty rods and irregular forms, 0.05–0.17 mm long and 0.01–0.035 mm wide (Figs 41C, small orange sclerites, 42C). Colour of the colony is brownish orange; the lower part of the branches is of a lighter hue.

##### Distribution.

Unknown.

##### Remarks.

The species was described by Aurivillius (1931) based on specimens from an unknown locality in the southern Pacific. He described the species as new without comparing with any type material and remarked that the new species was similar to *Muricea robusta* (Aurivillius 1931). The two species indeed have some similarity discussed below. Presently we do not have specimens matching this species.

#### Muricea robusta

Taxon classificationAnimaliaAlcyonaceaPlexauridae

Verrill, 1864

Figures 43 , 44

Muricea robusta Verrill, 1864: 36; Verrill 1869: 436–437; Kükenthal 1919: 752; Kükenthal 1924: 144; Riess 1929: 396–397; Harden 1979: 159.
Muricea robusta (pars.) Verrill 1866: 329.

##### Material.

Lectotype. YPM 1189a, dry, Acapulco, Mexico, A.E. Agassiz, 1859–1860, no more data. Paralectotypes. MÉXICO: MCZ 189; MZUC-ANT 195 (part of MCZ 189); YPM 1712 (figured fragment, Verrill 1869); YPM 1189b, dry, Acapulco, A.E. Agassiz, 1859–1860, no more data.

##### Description.

**Figure 43.**
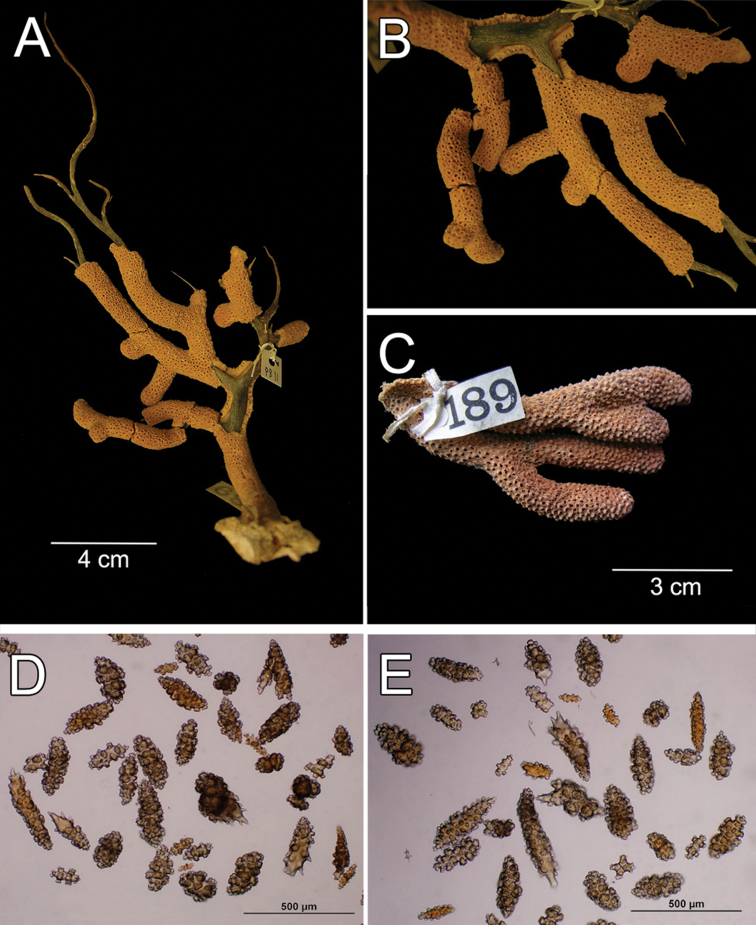
*Muricea robusta* Verrill, 1864. **A** Colony, YPM 1189a **B** Detail of branches, YPM 1189a **C** Detail of calyces, ZMUC ANT 195 (YPM 1189a. (189) fragment) **D–E** Sclerites, light micrographs, YPM 1189a.

**Figure 44.**
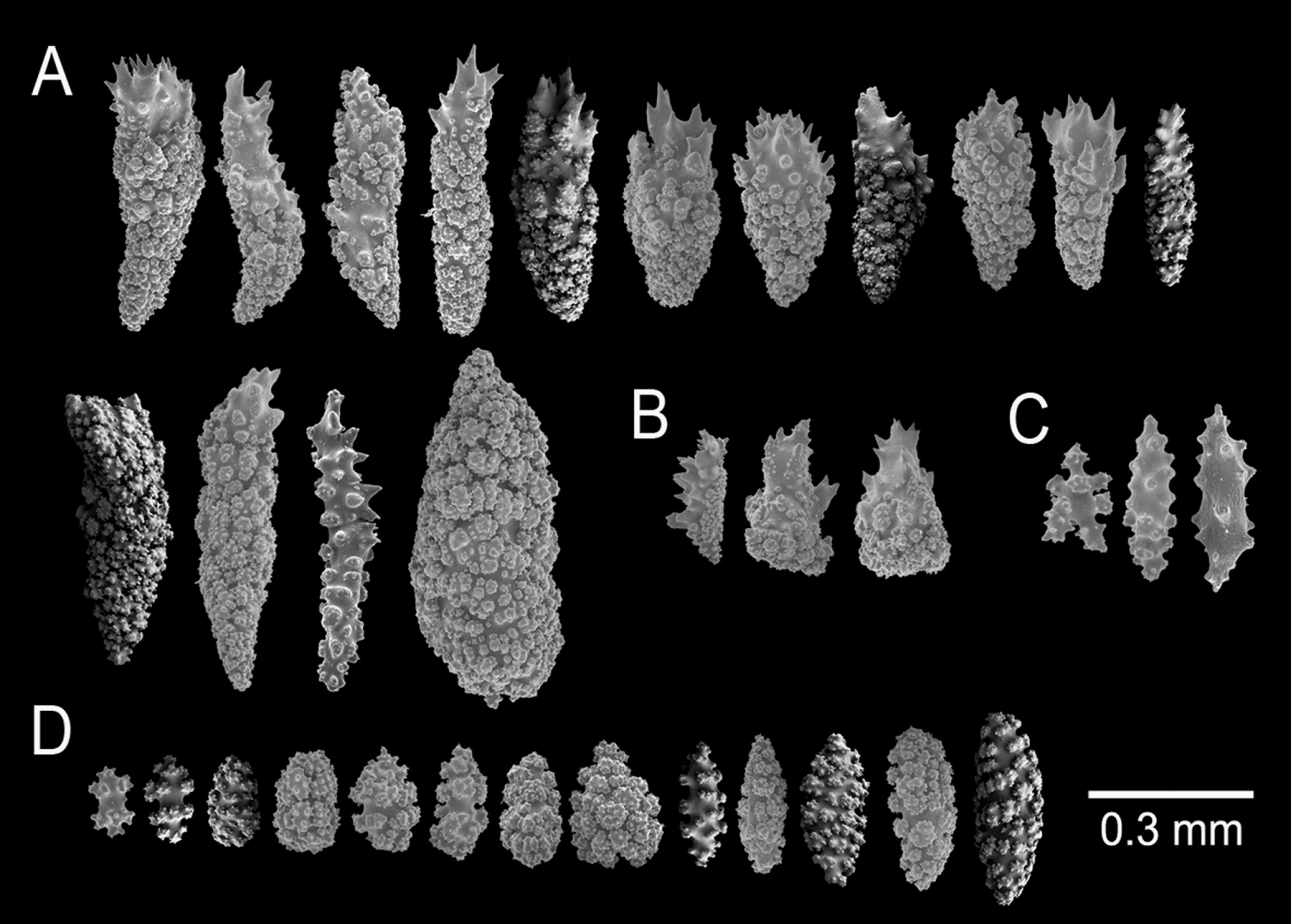
*Muricea robusta* Verrill, 1864. YPM 1189a. **A–B** Calycular and coenenchymal sclerites **C** Anthocodial sclerites **D** Axial sheath sclerites.

The lectotype is a 20 cm long and 10 cm wide colony with partially broken coenenchyme on some branches and with three naked distal axes (Fig. 43A). A slightly flattened stem, 11 mm in diameter, arises from an oval holdfast, about 4 cm in diameter (Fig. 43A). The branching is mostly dichotomous in one plane (Fig. 43A). The stem extends up to 36 mm and subdivides in two main branches, which bifurcate up to 5 times producing secondary branches and branchlets of less than 10 mm in diameter, all of similar diameter. The branches bifurcate at angles 45°–60°, 3.5–5 mm apart; the branchlets, that are short, are almost at right angles. The upper branches are curved inferring from the naked axes (Fig. 43A). Unbranched terminal ends are up to 16 mm long, with rounded tips, up to 8.5 mm in diameter. The naked unbranched terminal ends reach up to 70 mm long. No anastomosing branches are present. Axes are black and brownish at the tips. The calyces are all around the branches and close together. They are mostly low cones, 0.7–1.2 mm in height, with a slightly elevated margin around the polyps (Fig. 43B) that are more prominent towards the end of the branches (Fig. 43C). At the upper branches, the lower margin of the calyx curves upwards. The polyp apertures are large, and conspicuous. The coenenchyme is thick and granulose, composed of orange, brownish orange and light brown to whitish sclerites (Fig. 43D–E). As in many other species in this genus, a division of sclerite layers is not clear and the coenenchyme is formed of a combination of several types of sclerite types intermingled. The coenenchymal and calycular sclerites are mostly the same type of spindles. The unilateral spinous spindles were rare in the samples, the larger sclerites are rather irregular spindles, bent or almost straight; the larger ones are covered with warts, almost up to the thorny end, and the others have a pronounced upper ridge of large spines (prickly spindles). The term cristate, suggested by Hickson (1928), could be applied to these sclerites (Fig. 44A–B). They are 0.24–0.64 mm long and 0.08–0.26 mm wide. There are some small leaf-like spindles and irregular cristate forms (Fig. 44B). The axial sheath and the inner coenenchyme have some conspicuous sub-spheroidal sclerites densely covered with warty tubercles, warty spindles, and some cylinder-like sclerites, 0.30–0.40 mm long and 0.13–0.19 mm wide, and tuberculate capstans, 0.16–0.25 mm long and 0.08–0.12 mm wide (Fig. 44D). Anthocodial sclerites are orange warty rods and irregular forms, 0.043–0.15 mm long and 0.04–0.05 mm wide (Fig. 44C). Colour of the colony is brownish orange.

##### Variability.

The revised specimens are consistent with the lectotype.

##### Distribution.

Found in México in Venado Island by J.L. Carballo and reported for Cape San Lucas (Harden 1979). Also found in Isla del Gallo, Colombia by Prahl et al. (1986) Type locality Acapulco, México.

##### Remarks.

The species was first mentioned by Verrill in 1864 and formerly described in 1869 with a specimen from Acapulco. We believe that Verrill’s type series are all fragments of the same colony; however, because this is not clear, herein we designated the specimen YPM 1189a as the lectotype of the species with the purpose of clearly establishing its taxonomic status.

##### Other material revised.

COLOMBIA: USNM 79466, dry, Isla del Gallo, near Tumacao, 0.5 m, H. von Prahl, 4 February 1982. MÉXICO: M12, dry, Venados Island, Mazatlan Bay, Sinaloa, 5–25 m, J.L. Carballo, 2 February 1999.

### Species-group comparison summary

The *Muricea austera*-group comprises eight species: *Muricea austera*, *Muricea albida*, *Muricea crassa*, *Muricea retusa*, *Muricea purpurea*, *Muricea nariformis*, *Muricea hebes* and *Muricea robusta*. The group is characterised by thick coenenchyme, thick branches and stout colonies that can be bushy, finger-like or candelabrum-like. Species comparisons are in Tables 1–2. The colour of the colony in this genus is remarkably constant (Verrill 1869, Hickson 1928). Therefore, it could be used as a primary character to separate species in the *Muricea austera*-group. First, colonies from deep brown to yellowish brown including *Muricea crassa*, *Muricea robusta*, *Muricea nariformis*, *Muricea austera* and *Muricea hebes* (ordered from darker to lighter colour). *Muricea crassa* is different from all others, especially in the larger size, thickness of the branches and the large rough calyces. The larger sizes of the sclerites are also found in *Muricea fruticosa* and *Muricea echinata* (in the *Muricea fruticosa* group), up to 2.4 mm long, but the species are different in many aspects, as discussed under the *Muricea fruticosa* group. *Muricea nariformis* and *Muricea robusta* differ from the others in the group by the leaf-like spindle as the dominant type of sclerites and the lowest calyces (Tables 1–2). Aurivillius (1931) noticed that *Muricea nariformis* was similar to *Muricea robusta*, with respect to the calyx shape and height, the colours of the colony and sclerites. Considering that Aurivillius never saw a specimen of *Muricea robusta*, he was rather correct. It is likely that *Muricea nariformis* is just a morphotype of *Muricea robusta*, showing just a different growing pattern and a lighter colour, but more specimens and field observation will be needed to synonymise them in the future. *Muricea austera* and *Muricea hebes* are similar in the colour of colony and sclerites, but differ in other characteristics (Table 2), especially in the sclerite sizes that are much smaller in *Muricea hebes*. According to Verrill *Muricea austera* is comparable with *Muricea echinata* (in the *Muricea fruticosa* group), especially in the colour of the colony and external appearance. However, *Muricea austera* differs from *Muricea echinata* in the thicker coenenchyme, smaller sclerites and shorter, broader and conical calyces (Tables 1–2).

The second group contains white colonies, including only *Muricea albida*. In this group (Table 2), the sclerites are similar in shapes and sizes to *Muricea austera* but they are not white or colourless as in *Muricea albida*. It could be compared with *Muricea formosa* but differs in many other aspects as discussed under *Muricea fruticosa* group.

The third group contains reddish purple colonies, including *Muricea retusa* and *Muricea purpurea*. These species are similar also in the dichotomous branching, the arrangement of the calyces and the calyx sclerites (in rows) (Tables 1–2). The main difference is the lack of large unilateral spinous spindles in *Muricea purpurea*. *Muricea purpurea* as established here differs from most of the other species in the *Muricea austera*-group in the relatively small size of the sclerites and the abundant and characteristic leaf-like spindles.

## Conclusion

**Figure 45.**
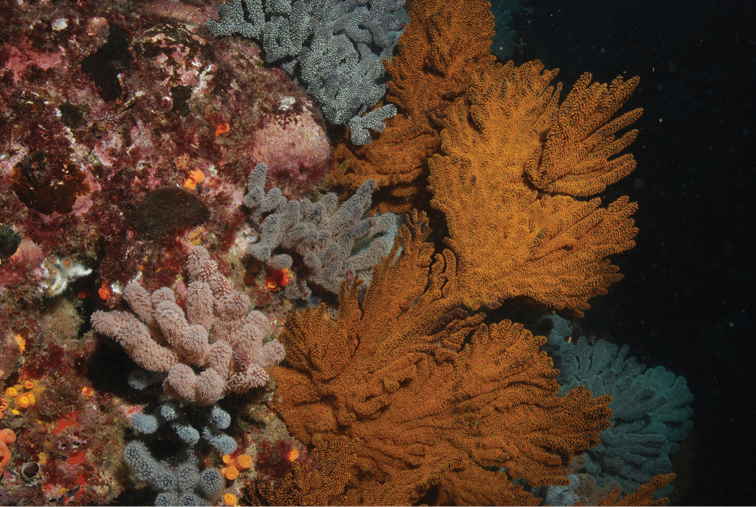
*Muricea* species, *Muricea purpurea* (left side) and *Muricea plantaginea* (right side). Ahorcados Islet, Machalilla National Park, Ecuador. Photograph: Graham Edgar.

We conclude that the genus *Muricea* in the eastern Pacific comprises 20 valid species that could be separated by their morphological characters into four groups: the *Muricea squarrosa*-group represented by four species with tubular calyces (Breedy and Guzman 2015), and 16 species with shelf-like calyces, *Muricea fruticosa*-group, *Muricea plantaginea*-group and *Muricea austera*-group herein presented. There is a range of variation among group-species, but a larger number of specimens might be examined in order to decide about close species relationships. Some species are abundant while others are known only from type material. Presently, the most common species found were *Muricea austera*, *Muricea purpurea*, *Muricea plantaginea* (see Fig. 45) and *Muricea fruticosa*. They were found in clusters scattered over rocky outcrops, platforms or on muddy and sandy bottoms attached to debris, shells or any solid object. The deepest record is *Muricea fruticosa*, down to 100 m deep at Cocos Island seamounts (Fig. 4B) and Hannibal Bank seamount, Panamá. Future exploration could render more species with a wide distribution range.

## Supplementary Material

XML Treatment for Muricea

XML Treatment for Muricea fruticosa group

XML Treatment for Muricea formosa

XML Treatment for Muricea aspera

XML Treatment for Muricea echinata

XML Treatment for Muricea galapagensis

XML Treatment for Muricea plantaginea

XML Treatment for Muricea californica

XML Treatment for Muricea mortensenii

XML Treatment for Muricea austera

XML Treatment for Muricea albida

XML Treatment for Muricea crassa

XML Treatment for Muricea retusa

XML Treatment for Muricea purpurea

XML Treatment for Muricea hebes

XML Treatment for Muricea nariformis

XML Treatment for Muricea robusta

## References

[B1] Aurivillius M (1931) The gorgonians from Dr. Sixten Bock’s expedition to Japan and the Bonin Islands, 1914. Kungliga Svenska Vetenskapsakademiens Handlingar (ser. 3) 9: 1–337.

[B2] Bayer FM (1956) Octocorallia, Part F. Coelenterata. In: Moore RC (Ed.) Treatise on Invertebrate Paleontology. Geological Society of America and University of Kansas Press, Lawrence-Kansas, F166–F231.

[B3] Bayer FM (1959) Octocorals from Surinam and the adjacent coasts of South America. Studies on the Fauna of Suriname and other Guyanas, 6: 1–43.

[B4] Bayer FM (1961) The shallow-water Octocorallia of the West Indian Region: (A manual for marine biologists). In: Hummelinck W (Ed.) Studies on the Fauna of Curacao and other Caribbean Islands 12(55): 1–373.

[B5] Bayer FM (1981) Key to the genera of Octocorallia exclusive of Pennatulacea (Coelenterata: Anthozoa) with diagnoses of new taxa. Proceedings of the Biological Society of Washington 94(3): 902–947.

[B6] Bayer FM (1994) A new species of the gorgonacean genus *Muricea* (Coelenterata: Octocorallia) from the Caribbean Sea. Precious Corals & Octocoral Research 3: 23–27.

[B7] Bayer FM Grasshoff M Verseveldt J (1983) Illustrated Trilingual Glossary of Morphological and Anatomical Terms Applied to Octocorallia. E.J. Brill/Dr. W. Backhuys, Leiden-the Netherlands, 75 pp.

[B8] Blainville HMD de (1834) Manuel d’Actinologie ou de Zoophytologie. FG Levrault, Paris, i-viii + 1–644, 633–694 pp.

[B9] Breedy O Guzman HM (2002) A revision of the genus *Pacifigorgia* (Coelenterata: Octocorallia: Gorgoniidae). Proceedings of the Biological Society of Washington 115(4): 782–839.

[B10] Breedy O Guzman HM (2007) A revision of the genus *Leptogorgia* Milne Edwards & Haime, 1857 (Coelenterata: Octocorallia: Gorgoniidae) in the eastern Pacific. Zootaxa 1419: 1–90.

[B11] Breedy O Guzman HM (2011) A revision of the genus *Heterogorgia* Verrill, 1868 (Anthozoa: Octocorallia: Plexauridae). Zootaxa 2995: 27–44.

[B12] Breedy O Guzman HM (2015) A revision of the genus *Muricea* Lamouroux, 1821 (Anthozoa, Octocorallia) in the eastern Pacific. Part I: *Eumuricea* Verrill, 1869 revisited. Zookeys 537: 1–32. doi: 10.3897/zookeys.537.6025 26798234 10.3897/zookeys.537.6025 PMC4714044

[B13] Breedy O Guzman HM Vargas S (2009) A revision of the genus *Eugorgia* Verrill, 1868 (Coelenterata: Octocorallia: Gorgoniidae). Zootaxa 2151: 1–46.

[B14] Breedy O Hickman CP Williams G Jr. (2009) Octocorals in the Galapagos Islands. Galapagos Research 66: 27–31.

[B15] Castro CB Medeiros MS Loyola LL (2010) Octocorallia (Cnidaria: Anthozoa) from Brazilian reefs. Journal of Natural History 44: 763–827. doi: 10.1080/00222930903441160

[B16] Covarrubias OA Duarte FS Reyes-Bonilla HR (1996) Range extension of Muricea hebes (Gorgonacea: Plexauridae) to the Gulf of California. Revista de Biología Tropical 44(2): 941–950.

[B17] Dana JD (1846) Zoophytes. United States Exploring Expedition during the years 1838, 1839, 1840, 1841, 1842, under the command of Charles Wilkes, U.S.N. Vol. 7 Lea and Blanchard, Philadelphia, i-vi + 1–740 pp. PMC10420750 38209897

[B18] Deichmann E (1936) XLIX. The Alcyonaria of the western part of the Atlantic Ocean. Memoirs of the Museum of Comparative Zoology at Harvard College, Vol. LIII Cambridge, Massachsetts, 317 pp.

[B19] Deichmann E (1941) Coelenterates collected on the Presidential Cruise of 1938. Smithsonian Miscellaneous Collections 99, 10, 1–17.

[B20] Duchassaing P Michelotti G (1864) Supplement au mémoire sur les Coralliaires des Antilles. Memorie della Reale Accademia delle Scienze di Torino (ser. 2) 23: 97–206.

[B21] Ehrenberg CG (1834) Beitrage zur physiologischen Kenntniss der Corallenthiere im allgemeinen, und besonders des rothen Meeres, nebst einem Versuche zur physiologischen Systematik derselben. Abhandlungen der Königlichen preussischen Akademie der Wissenschaften zu Berlin. Aus dem Jahre 1832. Erster Theil, 1–380.

[B22] Gorzawsky H (1908) Die Gorgonaceenfamilien der Primnoiden und Muriceiden. Inaugural-Dissertation zur Erlangung der philosophischen Doktorwurde der hohen philosophischen Fakultat der Kongelige Universität Breslau, Buchdruckerei H. Fleischmann, Breslau.

[B23] Grigg RW (1972) Orientation and growth form of the sea fans. Limnology and Oceanography 17: 185–192. doi: 10.4319/lo.1972.17.2.0185

[B24] Grigg RW (1977) Population dynamics of two gorgonian corals. Ecology 58: 278–290. doi: 10.2307/1935603

[B25] Gray JE (1859) On the arrangement of zoophytes with pinnated tentacles. Annals and Magazine of Natural History 4(3): 439–444.

[B26] Haeckel E (1866) Generelle Morphologie der Organismen. Berlin, 1036 pp. doi: 10.1515/9783110848281

[B27] Hardee M Wicksten MK (1996) Redescription and taxonomic comparison of three eastern Pacific species of *Muricea* (Cnidaria: Anthozoa). Bulletin of the Southern California Academy of Sciences 95(3): 127–140.

[B28] Harden DG (1979) Intuitive and Numerical Classification of East Pacific Gorgonacea (Octocorallia). PhD thesis, Illinois State University, Illinois, USA.

[B29] Hickman CP (2008) A field guide to corals and other radiates of Galápagos. Sugar Spring Press, Lexington Virginia-USA, 162 pp.

[B30] Hickson SJ (1928) Papers from Dr. Th. Mortensen’s Pacific Expedition 1914–16. XLVII. The Gorgonacea of Panama Bay together with a description of one species from the Galápagos Islands and one from Trinidad. Videnskabelige Meddelelser Fra Dansk Naturhistorisk Forening 85: 325–422.

[B31] Kölliker RA von (1865) Icones histiologicae oder Atlas der vergleichenden Gewebelehre. Zweite Abtheilung. Der feinere Bau der hoheren Thiere. Erstes Heft. Die Bindesubstanz der Coelenteraten. Verlag von Wilhelm Engelmann, Leipzig, [i-iv] + 87–181 pp.

[B32] Kükenthal W (1919) Gorgonaria. Wissenschaftliche Ergebnisse der deutsche Tiefsee-Expeditionen “*Valdivia*” 1898–99 13(2): 1–946.

[B33] Lamarck JB (1815) Sur les polypiers corticiferes. Mémoires du Muséum Histoire Naturelle. Paris, 2: 157–164.

[B34] Kükenthal W (1924) Gorgonaria. Das Tierreich, Vol. 47 Walter de Gruyter and Company, Berlin, und Leipzig, 478 pp.

[B35] Lamouroux JVF (1812) Extrait d’un mémoire sur la classification des Polypiers coralligènes non entièrement pierreux. Nouveau Bulletin des Sciences, par la Société Philomathique, Paris 3: 181–188.

[B36] Lamouroux JVF (1821) Exposition méthodique des genres de l’ordre des polypiers, avec leur description et celles des principales espèces, figurées dans 84 planches; les 63 premières appartenant a l’Histoire Naturelle des Zoophytes d’Ellis et Solander. Paris, chez Mme Veuve Agasse, Paris, i-viii + 1–115, pls. 1–84. doi: 10.5962/bhl.title.11328

[B37] Marques ACSJ Castro CB (1995) *Muricea* (Cnidaria, Octocorallia) from Brazil, with description of a new species. Bulletin of Marine Science 567(1): 161–172.

[B38] Milne Edwards H Haime J (1857) Histoire naturelle des coralliaires ou polypes proprement dits, Vol. 1. à la Libraire Encyclopédique de Roret, Paris, 326 pp.

[B39] Möbius K (1861) Neue Gorgoniden des naturhistorischen Museums zu Hamburg. Nova Acta Acad. Caesareae Leopoldino-Carolinae Germanicae Nat Curiosorum 29: 1–12.

[B40] Nutting CC (1910) The Gorgonacea of the Siboga Expedition. III. The Muriceidae Siboga Expedition Monograph 13b: 108 pp.

[B41] Prahl H von Escobar D Molina G (1986) Octocorales (Octocorallia: Gorgoniidae y Plexauridae) de aguas someras del Pacifico colombiano. Revista de Biología Tropical 34(1): 13–33.

[B42] Riess M (1929) Die Gorgonarien Westindiens. Kapitel 8. Die Familie Muriceidae. Zoologische Jahrbuecher Systematik Supplement 16(2): 377–420.

[B43] Rivera F Martínez P (2011) Guía fotográfica de corales y octocorales, Parque Nacional Machalilla y Reserva de Producción Faunística Marino Costera Puntilla de Santa Elena. Fundación NAZCA, Conservación Internacional, Ecuador, 86 pp.

[B44] Stiasny G (1941) Studien uber Alcyonaria und Gorgonaria I-V. (Parerga und Paralipomena). Zoologische Anzeiger 133: 268–271.

[B45] Stiasny G (1943) Gorgonaria von Panamá. Aus der Sammlung Dr. Th. Mortensen, Zoologisk Museum, Kopenhagen Videnskavelige Meddelelser fra den naturhistoriske Forening i Kovenhavn for Aarene 107: 59–103.

[B46] Studer T (1887) Versuch eines Systemes der Alcyonaria. Archiv für Naturgeschichte 53(1): 1–74.

[B47] Tixier-Durivault A (1970) Octocoralliaires. Campagne de la “*Calypso*” au large des côtes atlantiques de l’Amérique du Sud (1961–1962). Annales de l’Institut Oceanographique 47: 145–169.

[B48] Valenciennes A (1846) Zoophytes. In: Abel Dupetit-Thouars, Voyage autour du monde sur la frégate la Vénus, pendant les années 1836–1839. Atlas de Zoologie, pls. 1–15.

[B49] Valenciennes A (1855) Extrait d’une monographie de la famille des Gorgonidees de la classe des polypes. Comptes Rendus Académie des Sciences, Paris 41: 7–15. doi: 10.5962/bhl.part.28683

[B50] Verrill AE (1864) List of the polyps and corals sent by the Museum of Comparative Zoology to other institutions in exchange, with annotations. Bulletin of the Museum of Comparative Zoology at Harvard College 1: 29–60.

[B51] Verrill AE (1866) On the polyps and corals from Panama with descriptions of new species. Proceedings of the Boston Society of Natural History 10: 323–357.

[B52] Verrill AE (1868a) Notes on Radiata in the Museum of Yale College. No. 6. Review of the corals and polyps of the West Coast of America. Transactions of the Connecticut Academy of Arts and Sciences, (Second Edition) 1(2): 377–422.

[B53] Verrill AE (1868b) Critical remarks on halcyonoid polyps in the museum of Yale College, with descriptions of new genera. American Journal of Science and Arts 45: 411–415.

[B54] Verrill AE (1869) Notes on Radiata in the Museum of Yale College, Number 6: Review of the corals and polyps of the West Coast of America. Transactions of the Connecticut Academy of Arts and Sciences (Second Edition) 1: 418–518.

[B55] Wright EP Studer T (1889) Report of the Alcyonaria collected by H.M.S. “Challenger” during the years 1873–91876. Challenger Reports: Zoology 31(64): 1–314.

